# NSC348884 cytotoxicity is not mediated by inhibition of nucleophosmin oligomerization

**DOI:** 10.1038/s41598-020-80224-1

**Published:** 2021-01-13

**Authors:** Markéta Šašinková, Petr Heřman, Aleš Holoubek, Dita Strachotová, Petra Otevřelová, Dana Grebeňová, Kateřina Kuželová, Barbora Brodská

**Affiliations:** 1 Department of Proteomics, Institute of Hematology and Blood Transfusion, U Nemocnice 1, 128 20 Prague 2, Czech Republic; 2 Faculty of Mathematics and Physics, Institute of Physics, Charles University, Ke Karlovu 5, 121 16 Prague 2, Czech Republic

**Keywords:** Biochemistry, Biophysics, Cancer, Cell biology

## Abstract

Nucleophosmin (NPM) mutations causing its export from the nucleoli to the cytoplasm are frequent in acute myeloid leukemia (AML). Due to heterooligomerization of wild type NPM with the AML-related mutant, the wild-type becomes misplaced from the nucleoli and its functions are significantly altered. Dissociation of NPM heterooligomers may thus restore the proper localization and function of wild-type NPM. NSC348884 is supposed to act as a potent inhibitor of NPM oligomerization. The effect of NSC348884 on the NPM oligomerization was thoroughly examined by fluorescence lifetime imaging with utilization of FRET and by a set of immunoprecipitation and electrophoretic methods. Leukemia-derived cell lines and primary AML cells as well as cells transfected with fluorescently labeled NPM forms were investigated. Our results clearly demonstrate that NSC348884 does not inhibit formation of NPM oligomers neither in vivo nor in vitro*.* Instead, we document that NSC348884 cytotoxicity is rather associated with modified cell adhesion signaling. The cytotoxic mechanism of NSC348884 has therefore to be reconsidered.

## Introduction

The human *NPM1* gene is located on the chromosome 5q35 and encodes a 32.6 kDa polypeptide. Nucleophosmin (NPM), encoded by the *NPM1* gene, is a ubiquitously expressed phosphoprotein residing predominantly in the granular component of the nucleolus and shuttling dynamically among nucleoli, the nucleoplasm and the cytoplasm^1–4^. It functions as a chaperone^5^ and is engaged in regulation of various cellular processes including the ribosome biogenesis^6^, DNA-damage repair^7^, centrosome duplication^8^, and DNA replication^9^. Furthermore, NPM is involved in apoptosis and can modulate p53 stability and activity^1,10^.

NPM overexpression, fusion, or mutation have oncogenic potential and are associated with cancer progression in many types of solid tumors^11^ and in hematopoietic malignancies^12–17^. Acute myeloid leukemia (AML) with mutated *NPM1* accounts for about 1/3 of de novo adult AML, and *NPM1* is the most frequently mutated gene in AML with normal karyotype (50–60% incidence)^12^. To date, more than 100 *NPM1* mutation types have been identified in AML, occurring almost exclusively in the last exon (exon 12) of the gene^18^. A nucleotide insertion and/or deletion (indel mutations) lead to the frame shift in the region encoding the C-terminus of NPM. All these *NPM1* mutations result in loss of tryptophan residues at positions 288 and/or 290, which form the main part of the nucleolar localization signal (NoLS) ensuring nucleolar localization of the wild-type NPM (NPMwt). Moreover, the indel mutations frequently generate an additional nuclear export sequence (NES), which labels the mutated protein for the nuclear exporter XPO1 and targets NPM into the cytoplasm^19^. Both the loss of NoLS and the occurrence of the new NES lead to the accumulation of the NPM mutant (NPMmut) in the cytoplasm^12,20,21^. Cytoplasmic NPM serves as an immunohistochemical marker with prognostic relevance^22–25^, and is also associated with reduced incidence of some HLA class I alleles, possibly due to anti-leukemia immune response^26,27^. In the absence of an additional genetic aberration, AML patients with *NPM1* mutation have better response to intensive chemotherapy.

NPM forms pentamers, which may assemble into decamers, through a conserved N-terminal domain^28–31^. This domain plays a crucial role in NPM interactions with many of its partners, e.g. with p14Arf or c-myc^1,32,33^. NPM with C-terminal mutation has been reported to retain the ability to form oligomers^34,35^. Therefore heterooligomers consisting of both NPMmut and NPMwt are frequent and the localization of individual variants is mutually affecting each other^20,36^. This results in a decrease of NPMwt concentration in the nucleolus and may thus cause a loss of function of the fraction of NPMwt delocalized into the cytoplasm^37^. Interaction of NPMmut with tumor suppressors also leads to aberrant transfer of these proteins into the cytoplasm and, presumably, to the loss or restriction of their biological function^38,39^. However, the role of the NPM mutation and of protein delocalization in AML initiation and the treatment response has not been elucidated yet.

In general, two approaches are tested to prevent delocalization of NPMwt and of its interaction partners together with the NPMmut into the cytoplasm. First, an inhibition of NPM nuclear exporter XPO1, and, the second, an impairment of the NPM oligomerization. Both approaches aim to reestablish the correct NPMwt localization^40,41^. A known XPO1 inhibitor Leptomycin B can block NPM transport into the cytoplasm, but it cannot be used for AML treatment owing to a high toxicity^25^. Alternative second-generation XPO1 inhibitors, selinexor and eltanexor, are drugs with promising anticancer effect. Selinexor is currently being tested in a phase I clinical trial^42^. Recently, its effect on complex formation between NPMmut and the transcription factor PU.1 with a key role in monocyte lineage differentiation has been demonstrated^43^.

In the present study, we focus on the second option, i.e. on the manipulation of NPM localization by an interference with NPM oligomerization. NPM oligomerization is mediated by its N-terminal domain. The equilibrium between the pentameric and monomeric forms is ruled mainly by posttranslational modifications, namely phosphorylation of numerous phosphosites^44^, and by interactions with proteins affecting NPM folding and assembly, in particular with p14Arf^45^. Recent findings suggest a role of the N-terminal region in nucleolar NPM localization linked to its interaction with proteins containing arginine-rich linear motifs and with ribosomal RNA^46^. This complements the original concept that NPM molecules are directed to the nucleolus by the nucleolar localization signal (NoLS) located in the very C-terminus of NPM^35^. In NPM mutants associated with AML, the alterations of the C-terminus result in loss of the NoLS. Consequently, AML-associated NPM mutations cause changes in the tertiary structure of the C-terminus^47^ that is responsible for significant aggregation tendency^48^. Although the C-terminal mutation was previously documented not to abrogate the NPM oligomerization ability^35^, we revealed that oligomers formed by NPMmut tend to dissociate into monomers more likely than oligomers formed by NPMwt^49^. Therefore, therapy based on the interference with NPM oligomerization might be beneficial for AML patients with *NPM1* mutation, as only NPMmut-consisting oligomers would be inhibited under optimal conditions and NPMwt could retain its function.

The small molecule NSC348884 was reported to prevent formation of NPM oligomers^50^. However, the action of NSC348884 was found to be rather complex. NSC348884 activates p53, inhibits cell growth, and triggers apoptosis^41,50^. In a number of recent works the effects of NSC348884 treatment are being ascribed in particular to “NPM inhibition”^51–53^ and authors by default assume that NSC348884 inhibits NPM oligomerization, as declared^41,50^. Nonetheless, in a set of cancer cell lines our experiments targeting NPM oligomerization by NSC348884 systematically exhibited surprisingly low effectiveness of the drug in this respect. Therefore we decided to focus on this single aspect of NSC348884 and to rigorously investigate its potential to inhibit NPM oligomerization both in vivo and in vitro.

To study the effect of NSC348884, it is crucial to validate a set of reliable methods for detection of NPM oligomers in cell lysates and living cells as well as to find trustable controls. The FLIM-FRET, native electrophoresis and immunoprecipitation, were therefore methods of choice for complex evaluation of the NSC348884 action. A set of NPM-mutants served as a control for a coherent validation of these methods and for assessment of their sensitivity to follow NPM oligomerization.

Having established a robust experimental and control system, we analyzed the effect of NSC348884 on NPM oligomerization in cell lysates and living cells. The effect of NSC348884 on proliferation and apoptosis is presented as well. In addition, unexpected significant NSC348884-induced decrease of cell adhesivity is described and a putative mechanism of NSC348884 action is proposed.

## Results

### Interaction and stability of NPM with C21 point mutation

Evaluation of NSC348884 ability to affect NPM oligomerization in living cells and cell lysates requires sensitive methods for oligomerization monitoring as well as reliable positive and negative controls. To closely simulate the native conditions, we preferentially searched for point mutations that were reported to inhibit NPM oligomerization.

**Figure 1:**
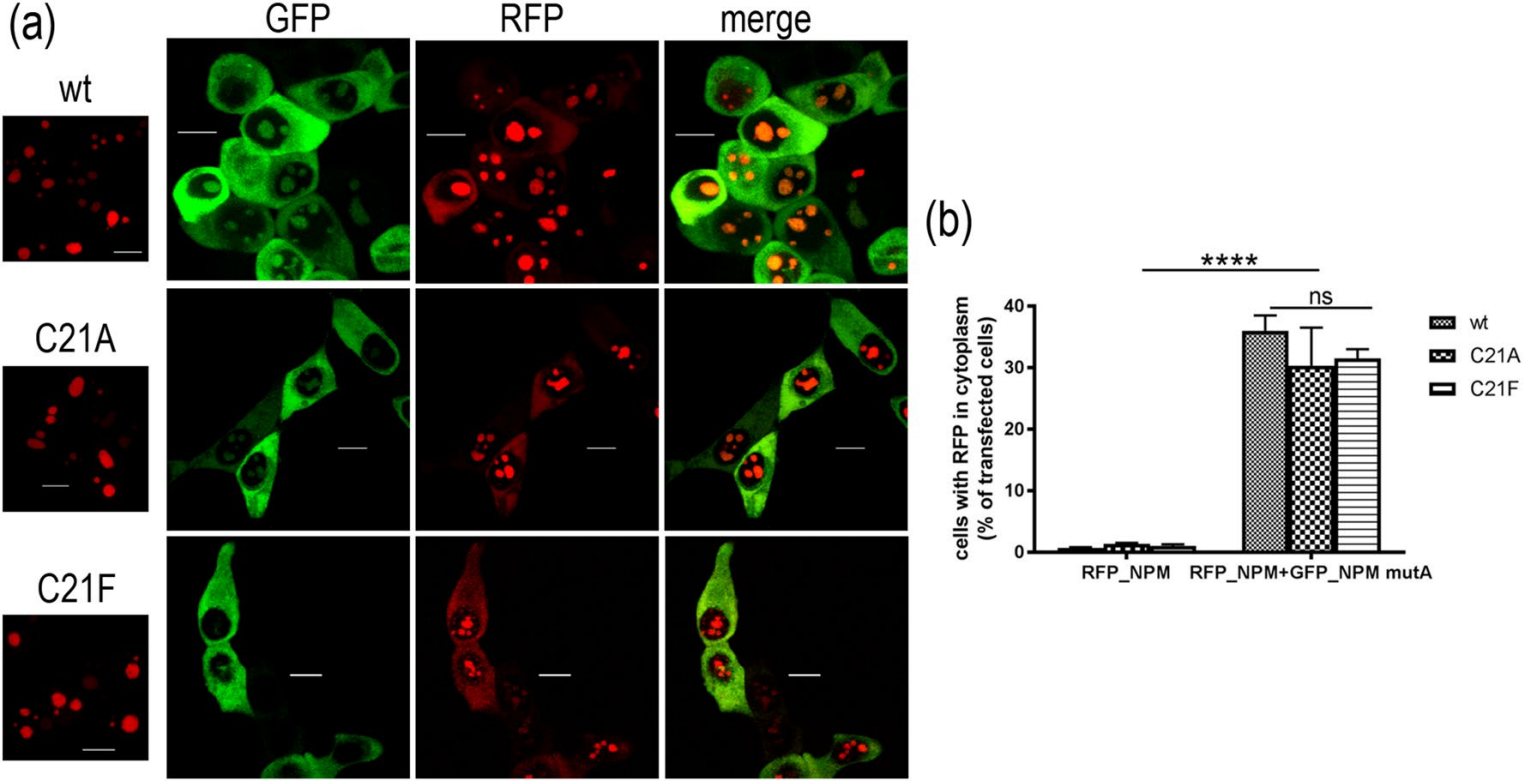
Interaction between NPMmut and C21 point mutants. (**a**) Left: localization of R_NPMwt/C21A/C21F in single transfected 293T cells. Right: 293T cells co-transfected with G_NPMmut (green) and R_NPMwt/C21A/C21F variants (red). Red signal in the cytoplasm and green signal in nucleoli witness for an interaction between NPMmut and NPMwt/C21A/C21F. (**b**) Statistical evaluation of the subcellular localization of NPMwt and C21 mutants. Fraction of transfected 293T cells displaying red signal from the cytoplasm in single transfected cells (left group) and in cells co-transfected with G_NPMmut (right group). Error bars represent ± SD of at least 3 independent experiments, *p* < 0.0001 (****) for the differences between the co-transfected and the single-transfected cells.

Point mutation in C21 was shown to be important for NPM oligomerization^54^. Recently, we have shown that conclusions about oligomerization of these mutants obtained under rather harsh conditions of the SDS-PAGE^54^ might strongly differ from results obtained in living cells by fluorescence lifetime imaging (FLIM-FRET) and by immunoprecipitation in lysates^55^. We demonstrated that some C21 NPM-mutants, originally expected not to oligomerize^34,54^, are actually able to form complexes with the endogenous NPM in living cells. In addition, immunoprecipitation revealed interaction of the endogenous NPM with eGFP-labeled NPM bearing point mutation at C21 (G_C21), i.e. where C21 was substituted either to Ala (G_C21A), or to Phe (G_C21F). In the present work, we used fluorescence microscopy and native electrophoresis to characterize in detail the impact of these C21 mutations. Both G_C21 mutants exhibited nucleolar localization, identical to that of NPMwt (Fig. 1). Furthermore, the mRFP1-labeled NPM_C21 (R_C21) was found in the cytoplasm of HEK-293T (293T) cells co-transfected with eGFP-labeled NPMmut (G_NPMmut). The fraction of cells exhibiting mRFP1 signal in the cytoplasm was comparable for all R_NPM variants (Fig. 1). Both C21 mutants therefore seem to form heterooligomers with NPMmut, alike NPMwt in living cells.

**Figure 2:**
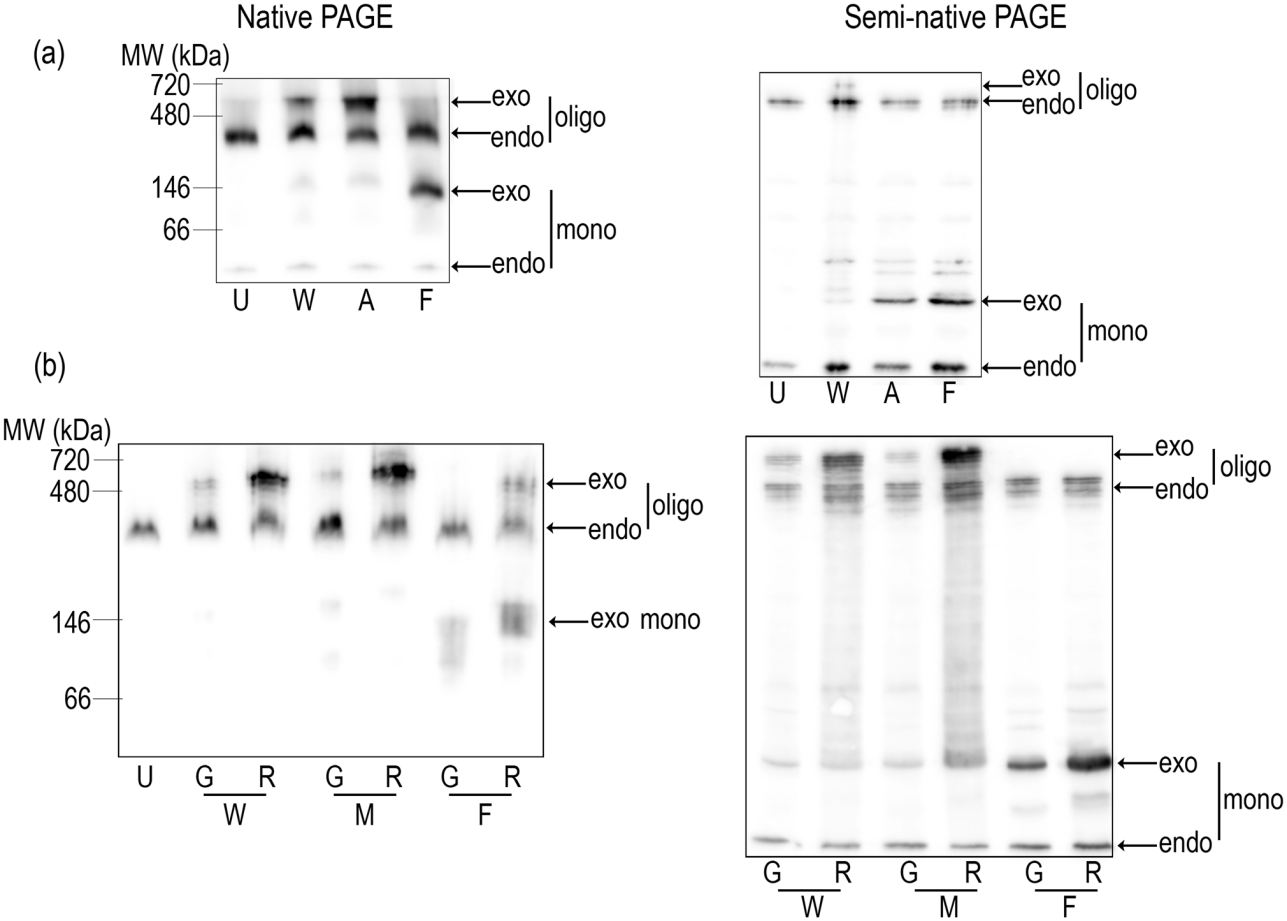
Effect of C21 mutations on the stability of NPM oligomers. Native (left) and semi-native (right) PAGE analysis of lysates from untransfected (U) 293T cells or cells transfected with NPMwt (W), C21A (A), C21F (F) or NPMmut (M). (**a**) Stability of oligomers formed by various G_C21 constructs is affected by the reducing PAGE conditions. (**b**) Position of bands and oligomer stability depend on the fluorescent label used (G or R: eGFP- or mRFP1-labeled proteins). The figures show representative examples from repeated experiments.

To independently verify these findings, we examined native lysates from cells expressing G_C21A or G_C21F by semi-native and native electrophoresis^49^. Briefly, the lysates from cells harvested into non-reducing, non-denaturing buffer were directly subjected without boiling to acrylamide gel with and without SDS, respectively (see Material and Methods). These relatively gentle separation methods allowed for detection of both NPM monomers and oligomers and for estimation of their electrophoretic mobility. The monomer/oligomer band intensity ratio in semi-native conditions reflects the propensity of oligomers to dissociate into the monomers. Results are shown in Fig. 2a. Whereas C21A exhibited a high-MW band (presumably oligomers) identical to that of NPMwt under native conditions, the band from C21F was located at the position corresponding to the weak lower-MW fraction of NPMwt or NPMmut (presumably monomers). In these experiments, the band from endogenous NPM oligomers served as a loading and position control. The results from semi-native electrophoresis in Fig. 2b show markedly increased monomer/oligomer ratio of C21A compared to NPMwt and absence of C21F oligomers. These results suggest that although NPM oligomerization seems to be unaffected by the C21 point mutation in living cells, the stability of the oligomers is considerably attenuated. Similar results were obtained with mRFP1-labeled variants. Interestingly, mRFP1-labeled proteins displayed slightly lower mobility in the native conditions. Moreover, R_NPM oligomers were found to be somewhat more stable compared to G_NPM ones (Fig. 2b).

**Figure 3:**
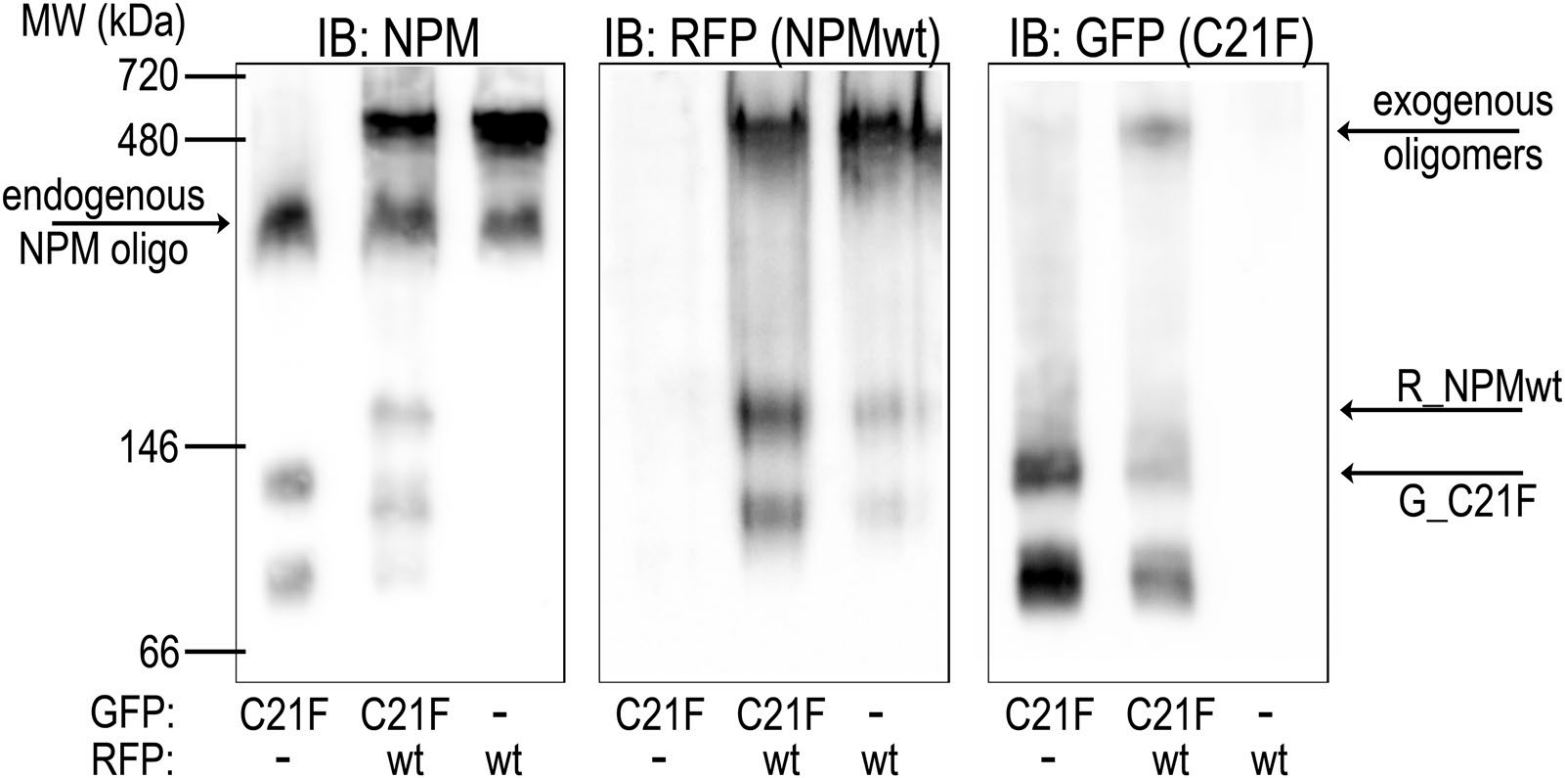
Formation of heterooligomers containing NPM with C21F substitution and NPMwt. Western blots of native PAGE of samples from 293T cells transfected with R_NPMwt (wt), G_C21F (C21F), and with their combination. Similar results were obtained with the inverse tagging, i.e. with G_NPMwt and R_C21F.

We have not observed any changes in expression and in oligomerization state of the endogenous NPMwt in cells containing C21 mutants. Therefore, we further investigated the effect of C21F substitution on the stability of exogenous heterooligomers formed by a mixture of fluorescently labeled C21F and NPMwt. 293T cells were alternatively transfected with single fluorescent variants of C21F and NPMwt, or co-transfected with the combination of both. As shown in Fig. 3 and Supplementary Fig. S1, the exogenous NPMwt monomer/oligomer ratio is strongly affected by the presence of C21F. Band intensities attributed to C21F oligomers and NPMwt monomers are both considerably higher in traits corresponding to the co-transfected sample than the ones in traits from single transfected cells. Distribution of NPM molecules between oligomer (high-MW) and monomer (low-MW) bands likely depends on the NPMwt/C21F participation in the heterooligomers. Interestingly, endogenous NPM monomer/oligomer ratio seems to be unaffected by the presence of any exogenous NPM. Altogether, despite the C21F mutation does not completely abrogate NPM oligomerization, it clearly attenuates interaction affinities underlying the mixed oligomer formation in living cells.

### Localization and oligomerization properties of NPM N-terminal deletion mutants

**Figure 4:**
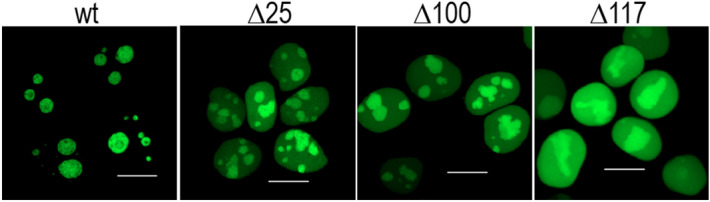
Significance of the N-terminus for NPM localization. Diminished nucleolar accumulation and increased amount in the nucleoplasm of Δ25, Δ100 and Δ117 compared to WT.

In view of the fact that the C21-point mutations do not cause any detectable changes of the NPM oligomerization in living cells, we searched for other modifications of the NPM oligomerization domain in order to validate detection methods for the oligomerization disruption. Numerous NPM N-terminal deletion mutants were reported to lose the oligomerization ability and the nucleolar localization depending on the extent and specificity of the deleted region^56^. Enomoto et al*.*^56^ determined residues and regions accountable for NPM oligomerization, for its nucleolar localization, and for p14Arf binding. Their data disclosed that NPM lacking a part of the N-terminal domain was localized in the nucleoplasm and exhibited inability to interact with other NPM molecules. For negative controls in living cells, we therefore created fluorescently labeled N-terminal NPM mutants with deletions of the first 25, 100 and 117 amino acids (Δ25, Δ100, Δ117) and we analyzed their subcellular localization and oligomerization characteristics. Our confocal imaging experiments have revealed that all the truncated NPM forms reside both in the nucleoli and in the nucleoplasm (Fig. 4). As expected, the largest deletion resulted in an increased accumulation of the mutant in the nucleoplasm.

**Figure 5:**
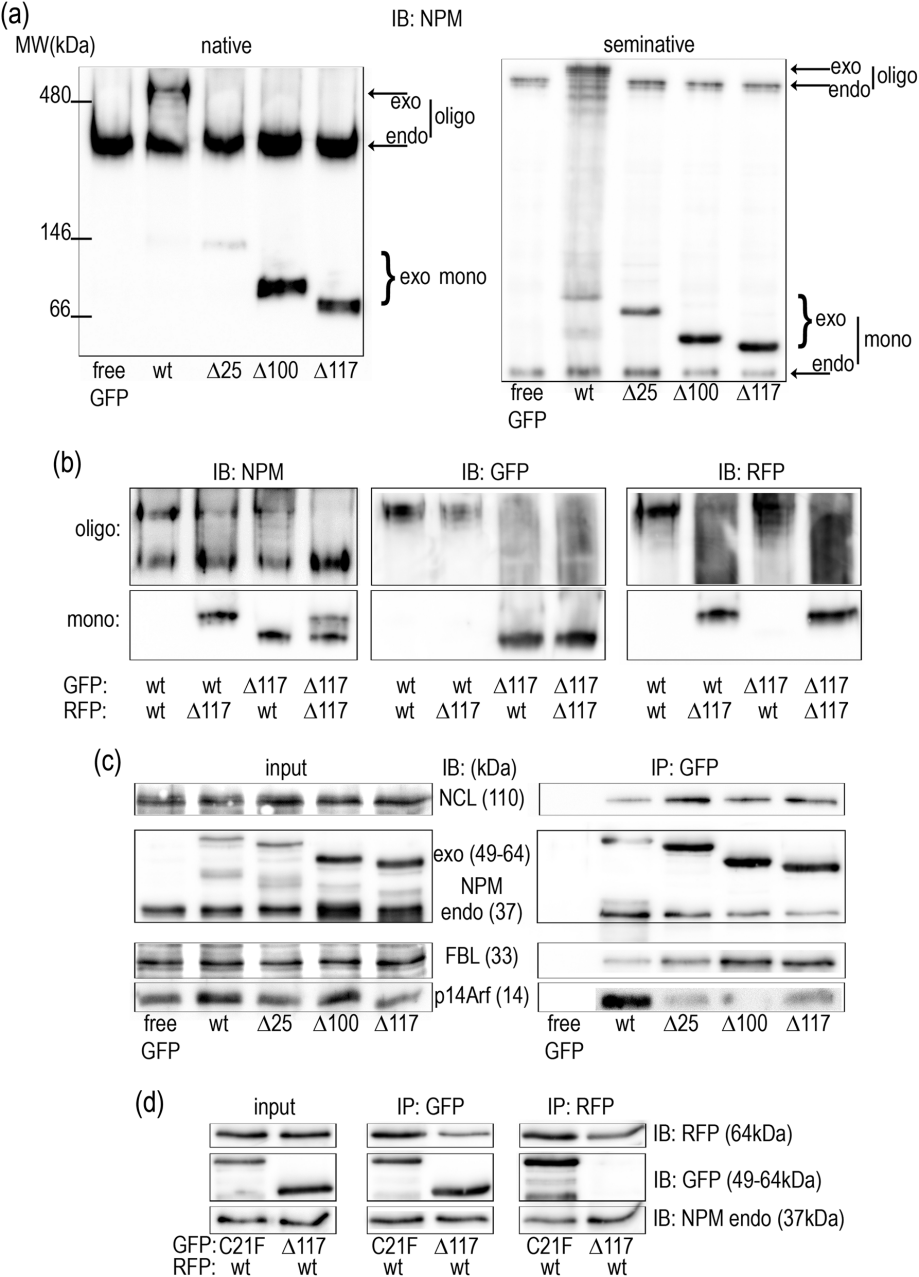
Significance of the N-terminus for NPM oligomerization. (**a**) NPM expression in native and semi-native PAGE of 293T cells transfected with free eGFP and eGFP-tagged truncated NPM variants. (**b**) Native PAGE of 293T cells co-transfected with combinations of Δ117 and NPMwt illustrating absence of interaction between Δ117 and NPMwt. (**c**) Interaction of truncated NPM forms with endogenous proteins. Lysates from 293T cells expressing GFP-labeled NPMwt, Δ25, Δ100 and Δ117 were subjected to immunoprecipitation and the levels of co-precipitated interaction partners were investigated. (**d**) eGFP/mRFP1-immunoprecipitation from 293T cells co-transfected with R_NPMwt and G_C21F or G_Δ117: asymmetric results of precipitation from the sample containing the truncated NPM form.

To monitor NPM complex formation, lysates from 293T cells transfected with fluorescently labeled deletion mutants were subjected to electrophoresis. As expected, inability of the truncated proteins to form oligomers was reflected by an absence of the high-MW bands under native and semi-native conditions (Fig. 5a). Further we searched for presence of NPM heterooligomers containing exogenous NPMwt and selected deletion mutants. 293T cells were co-transfected with plasmids ensuring expression of Δ117 and NPMwt and the lysates were analyzed (Fig. 5b and Supplementary Fig. S2). It can be seen that bands from oligomeric NPMwt complexes are unaffected by the presence of Δ117 in the native as well as in the semi-native immunoblots. The result suggests that, in contrast to the C21 mutants, the presence of the deletion mutants does not affect the oligomerization of NPMwt. Again, expression of the endogenous NPMwt remained unchanged. To analyze the interaction potential of the N-terminal deletion mutants, 293T cells were transfected with plasmids encoding for GFP-labeled NPMwt, Δ25, Δ100 or Δ117. Then immunoprecipitation using GFP-Trap was performed and the precipitates were analyzed for the presence of endogenous NPM as well as for presence of other nucleolar proteins known to interact with NPM. Surprisingly, although the GFP-tagged deletion mutants displayed no ability to oligomerize, interaction of the endogenous NPM with any deletion mutant (except the plasmid encoding for free eGFP) was detected. The amount of co-precipitated endo-NPM was only slightly lower compared to the G_NPMwt precipitates (Fig. 5c). Simultaneously, level of co-precipitated nucleolin (NCL), which does not interact with the AML-related NPMmut^49^, was higher in precipitates of the truncated NPM forms. The level of another co-precipitated nucleolar protein, fibrillarin (FBL), also positively correlated with the extent of N-terminus deletion. On the other hand, the tumor suppressor p14Arf, which is known to interact with the N-terminal NPM domain^56^, clearly co-precipitated only with the G_NPMwt.

To evaluate the utility of the method for detection of the complexes containing both NPMwt and the deletion mutants, we performed GFP/RFP-immunoprecipitation from cells co-transfected with R_NPMwt and G_∆117. The co-transfection of R_NPMwt and G_C21F served as an interacting control. Whereas NPMwt was clearly detected in samples obtained by precipitation of the deletion mutant, the vice versa co-precipitation failed (Fig. 5d). Identical results were obtained for combination NPMwt+Δ100. No interaction was found between two color variants of the N-terminal mutant (Supplementary Fig. S3).

**Figure 6:**
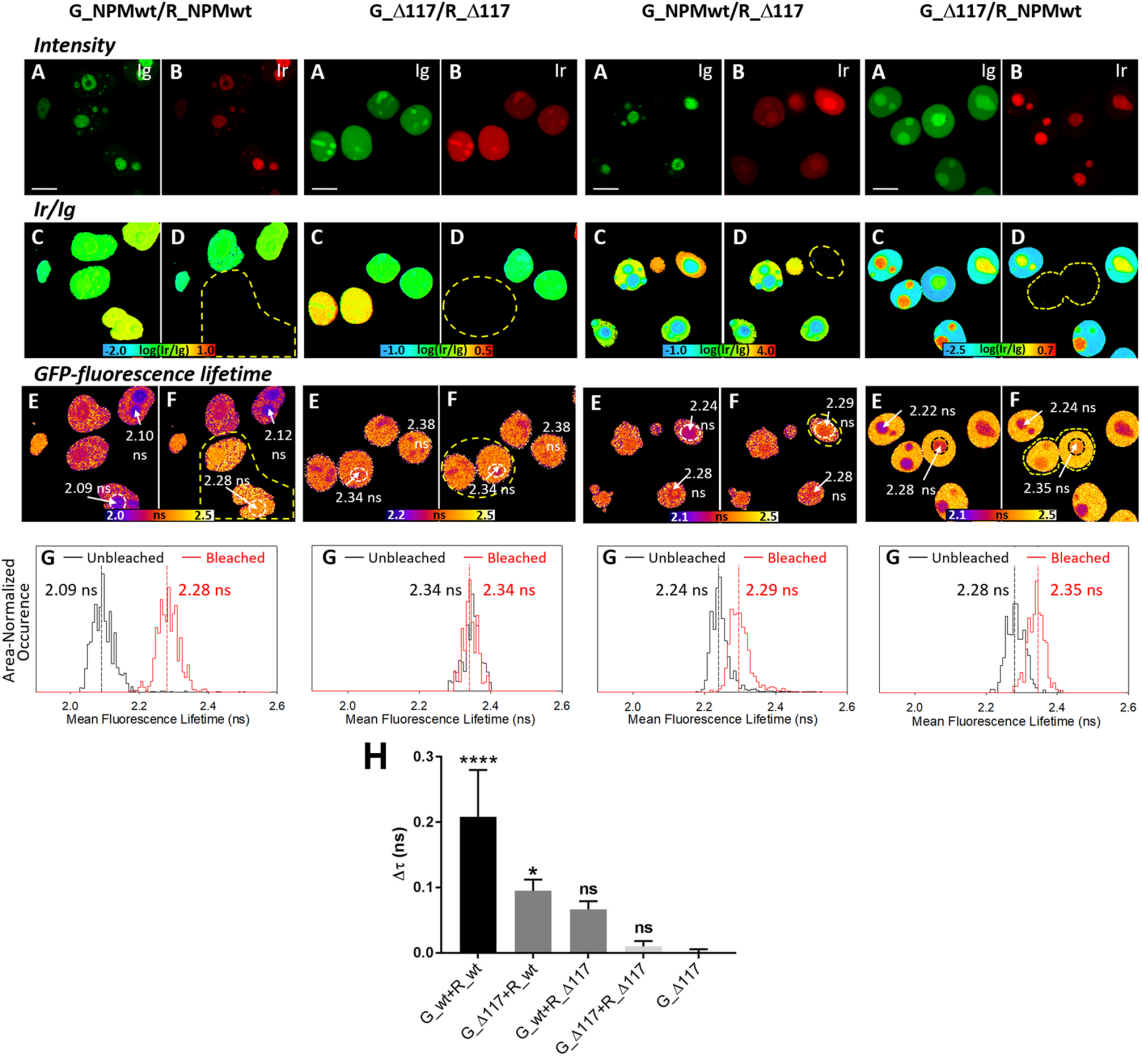
Effects of deletion in the N-terminal domain of NPM on its oligomerization in living 293T cells studied by FLIM-FRET. Interaction of eGFP- and mRFP1-labeled NPMwt causes shortening of the eGFP-fluorescence lifetime (*τ*) in co-transfected cells. After mRFP1-photobleaching, *τ* becomes prolonged, which confirms the G_NPMwt/R_NPMwt interaction. No lifetime change after mRFP1 photobleaching suggests absence of interaction between G_Δ117 and R_Δ117. (**A**,**B**) Initial localization of the green and red signal within the cells; (**C**,**D**) The intensity ratio of I_red_/I_green_ before and after the photobleaching, respectively. (**E**,**F**) Fluorescence lifetime distribution of eGFP before and after the mRFP1-photobleaching; (**G**) lifetime histograms from the analyzed nucleolar area; (**H**) One-way ANOVA analysis of the eGFP fluorescence lifetime change after acceptor photobleaching. Changes are compared to the negative control G_Δ117. Error bars represent ± SD of at least 3 independent experiments (****p < 0.0001; *p < 0.05).

The Förster resonance energy transfer (FRET) is a robust spectroscopic method for evaluation of a donor–acceptor proximity and works as a “ruler” on the nanometer scale. Due to the inverse 6th-power dependence of the transfer efficiency on the donor–acceptor distance^57^, the energy transfer occurs only between closely separated donor–acceptor pairs. This occurs e.g. within NPM oligomers comprising eGFP- and mRFP1-tagged subunits, where eGFP is a donor and mRFP1 acceptor. Complex formation therefore results in more efficient FRET, which is reflected in decreased fluorescence lifetime of eGFP. FRET-induced changes of the donor fluorescence lifetime can be mapped across the microscopic samples by fluorescence lifetime imaging (FLIM) ^58^. FRET-FLIM is perfectly suited for live-cell imaging and we previously successfully used it to detect complexes formed by NPMwt and C21 mutants in living cells^55^. In the present work, the FRET between eGFP as a donor and mRFP1 as an acceptor attached to NPMwt and Δ117 (Δ100) was examined. Presence of FRET within the complex was detected by the mRFP1 photobleaching, since an increase in the eGFP lifetime upon the mRFP1 photodestruction is a strong positive indicator of the mixed-complex formation. Results are shown in Fig. 6. Panels A and B show the initial localization of the green and red signal within the cells, panels C and D the intensity ratio I_red_/I_green_ before and after the photobleaching, respectively. Corresponding FLIM images and lifetime histograms from the analyzed nucleolar area are shown in the panels E, F and G, respectively. As expected, a significant lifetime increase resulting from the FRET cancellation was detected after photobleaching in cells co-transfected with G_NPMwt and R_NPMwt. On the contrary, virtually no effect was observed in cells transfected with the G_Δ117 + R_Δ117 mutants. In accord with the literature^56^ and with our precipitation data, this result indicates inability of the Δ117 mutants to interact with each other and to form multimers. This protein pair can therefore serve as a negative control for the multimer formation in the NSC348884 experiments. As seen from Fig. 6, in samples containing combination of the Δ117 deletion mutants with NPMwt, the GFP-lifetime slightly, but still visibly, increases upon the acceptor photobleaching. The change corresponds with the results of our electrophoretic experiments and suggests some amount of the mixed multimer to be present in the cell. The statistical analysis from multiple experiments (n = 3–5) is presented in Fig. 6H, where cells transfected with G_Δ117 (the donor only transfection) served as a negative control. The figure clearly proves oligomerization of NPMwt. Oligomerization of Δ117 deletion mutants is clearly undetected and the presence of mixed NPMwt + Δ117 multimers is at the significance limit. Interestingly, the statistical evaluation also suggests the asymmetric character of interaction between NPMwt and Δ variants. These results are in a nice agreement with the immunoprecipitation.

### Effect of NSC348884 on cell viability, apoptosis and NPM oligomerization

The small molecule NSC348884 was reported to interfere with NPM oligomerization in solid tumor cell lines^50^ as well as in leukemia cells^41^. Furthermore, it was proved to inhibit proliferation, to upregulate p53 and to trigger apoptosis^41,50^. We thus investigated the influence of NSC348884 treatment in a panel of leukemia cell lines complemented with HeLa and 293T cells.

**Figure 7:**
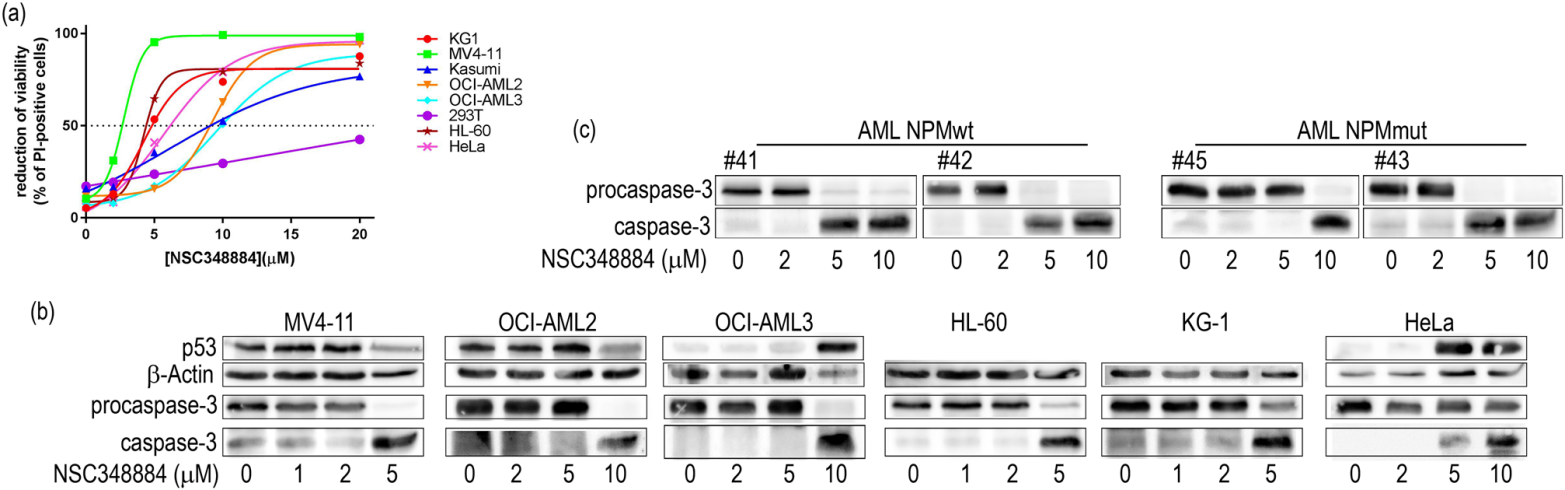
The effect of 24 h NSC348884-treatment on cell viability and apoptosis. (**a**) Cell viability monitored by propidium iodide exclusion: each point represents the mean value of 3–10 independent experiments. (**b**,**c**) Representative blots of caspase-3 fragmentation and p53 expression in cell lines (**b**) and in primary AML cells (**c**). β-Actin levels serve as a loading control.

**Figure 8:**
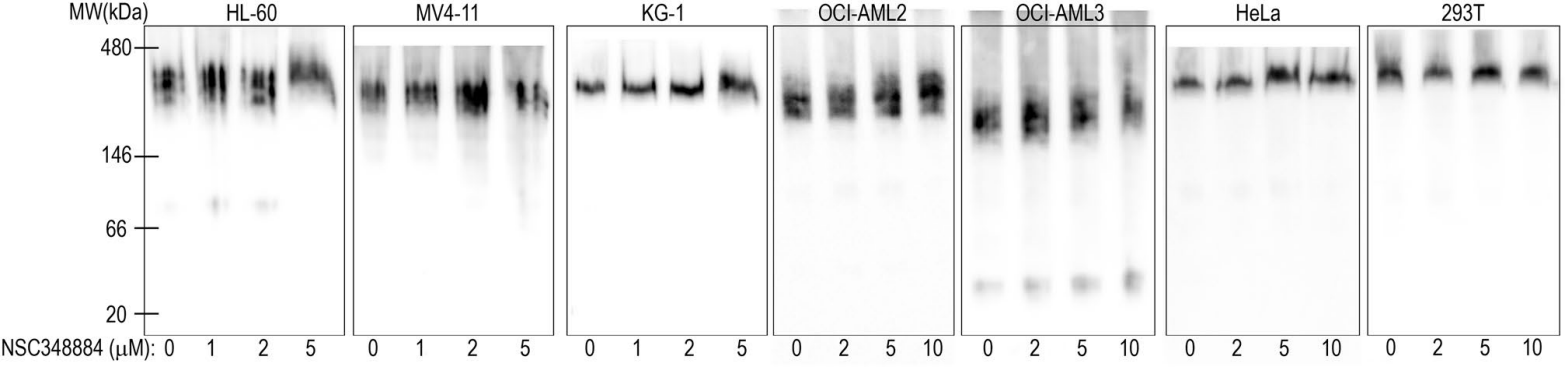
Effect of NSC348884 treatment on NPM oligomerization in leukemia cells. Native PAGE, representative blots: effect of 24 h NSC348884 treatment on NPM oligomerization in various leukemic cell lines as well as in adherent HeLa and 293T cells.

Prior to characterization of the NSC348884 effect on NPM oligomerization, we performed the analysis of cell viability and apoptotic markers. The cell viability in the presence of NSC348884 was monitored by propidium iodide (PI) exclusion test (Fig. 7a). Caspase-3 fragmentation as well as changes of p53 expression were investigated by immunoblotting to assess the extent of apoptosis in NSC348884-treated cells (Fig. 7b). For the majority of the cell lines, the EC50 value was within the interval from 2 to 10 µM. The viability drop correlated with increased caspase-3 fragmentation indicating the onset of apoptosis (Fig. 7b). Simultaneously, NSC348884-induced increase in the p53 level was detected in some of the cell lines possessing wild-type p53. Contrarily to previously reported results^41^, the majority of cell lines with NPMwt was more sensitive than the cell line with NPMmut (OCI-AML3). Comparable sensitivity (from caspase-3 fragmentation) to NSC348884 treatment was found also for the primary cells of AML patients regardless of their NPM mutational status (Fig. 7c). Unexpectedly, native PAGE experiments revealed no influence of NSC348884 on NPM oligomerization (Fig. 8). Endogenous NPM oligomers were found to be stable in KG-1, HL-60, MV4-11, and HeLa cell lines, which exhibited extensive apoptosis after NSC348884 treatment, as well as in OCI-AML2, OCI-AML3 and 293T, which were substantially more resistant to the treatment.

**Figure 9:**
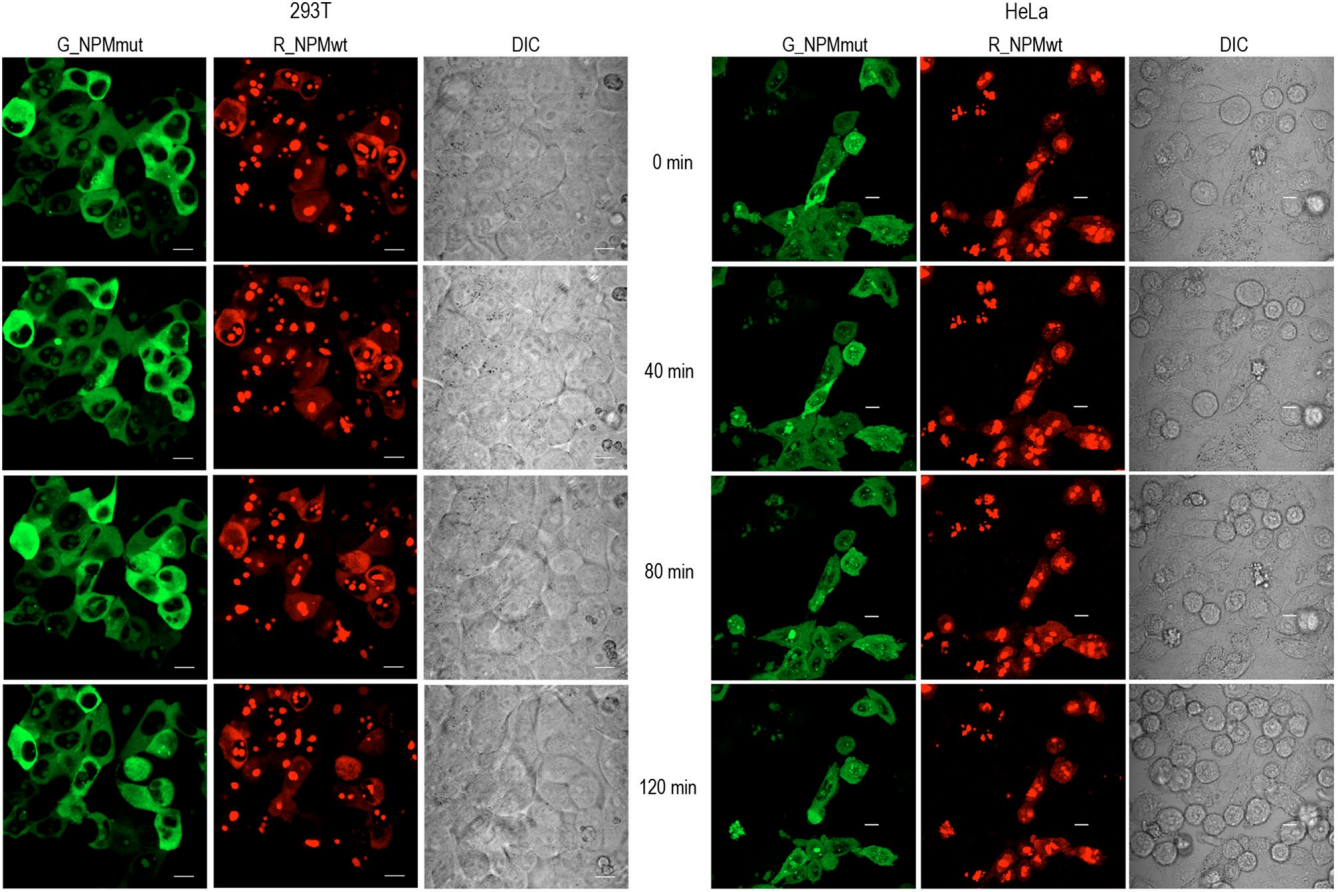
Time course of NSC348884-induced effects on the cell morphology. Effect of 10 µM NSC348884 was monitored in cells co-transfected with R_NPMwt (red) and G_NPMmut (green). The presence of the red signal in the cytoplasm and the cell morphology (DIC) were analyzed under confocal microscope. Left: 293T cells; right: HeLa cells. Scale bar represents 10 µm.

To further investigate effect of NSC348884 in vivo, we co-transfected 293T and HeLa cells with R_NPMwt and G_NPMmut. Then the cytoplasmic localization of R_NPMwt was monitored for 2 h after addition of 10 µM NSC348884 (Fig. 9). In agreement with our previous results^36,59^, detectable fraction of R_NPMwt was found in the cytoplasm of both cell lines at the starting time point. Lower cytoplasmic fraction of R_NPMwt in HeLa cells (compared to 293T) likely results from a higher endogenous NPM level^36,60^. Importantly, the cytoplasmic localization of R_NPMwt remained unchanged for at least 2 h after the treatment suggesting independence of NPM oligomerization on the presence of NSC348884 in vivo (Fig. 9). Simultaneously, there was an obvious effect of NSC348884 on cell-surface adhesivity. The effect is clearly visible in transmitted light images (DIC). Whereas the 293T cells progressively rounded and finally lost their contact with the glass surface of the culture dish, the HeLa cells detached from the surface individually. In any case, mitotic cells that detached from the surface for cell division have never re-adhered.

**Figure 10:**
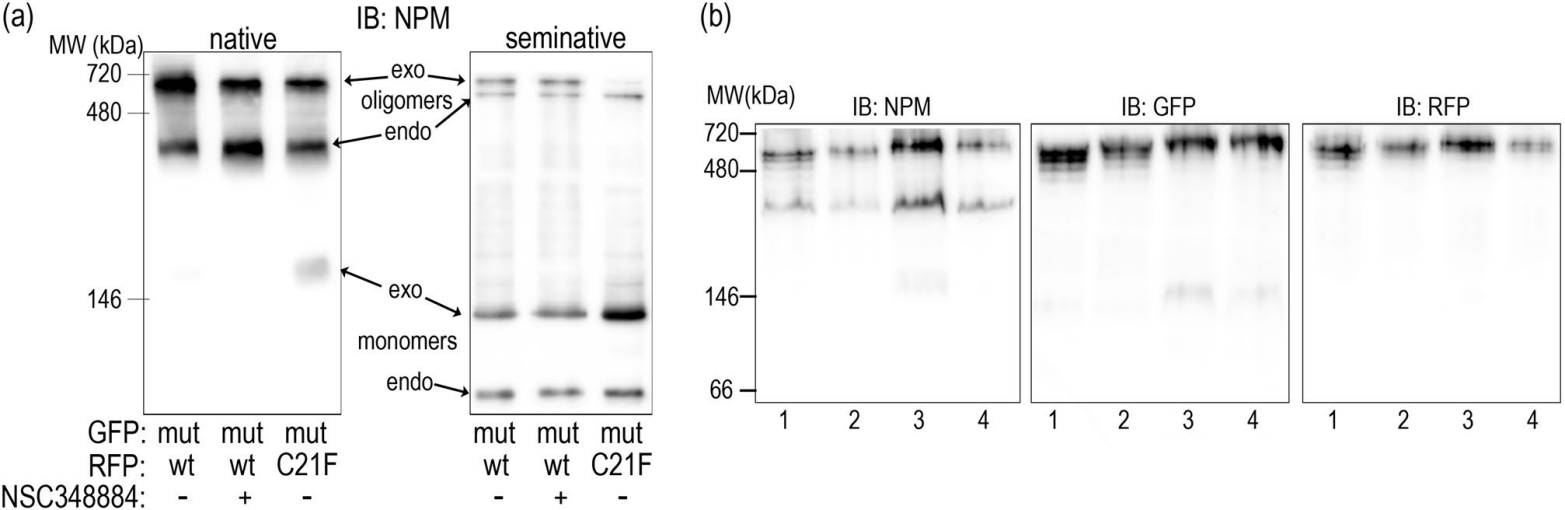
Native and semi-native PAGE from 293T cells transfected with various fluorescent variants of NPM and treated with 10 µM NSC348884 for 24 h. (**a**) Cells co-transfected with G_NPMmut and R_NPMwt (lanes 1 and 2) or R_C21F (lane 3). Endogenous NPM, R_NPMwt and G_NPMmut oligomers detected in untreated (lanes 1 and 3) and NSC348884-treated (lane 2) cells. (**b**) Native PAGE, G_NPMwt + R_NPMwt (1, 2) and G_NPMmut + R_NPMmut (3, 4) in control (1, 3) and NSC348884-treated (2, 4) cells.

**Figure 11:**
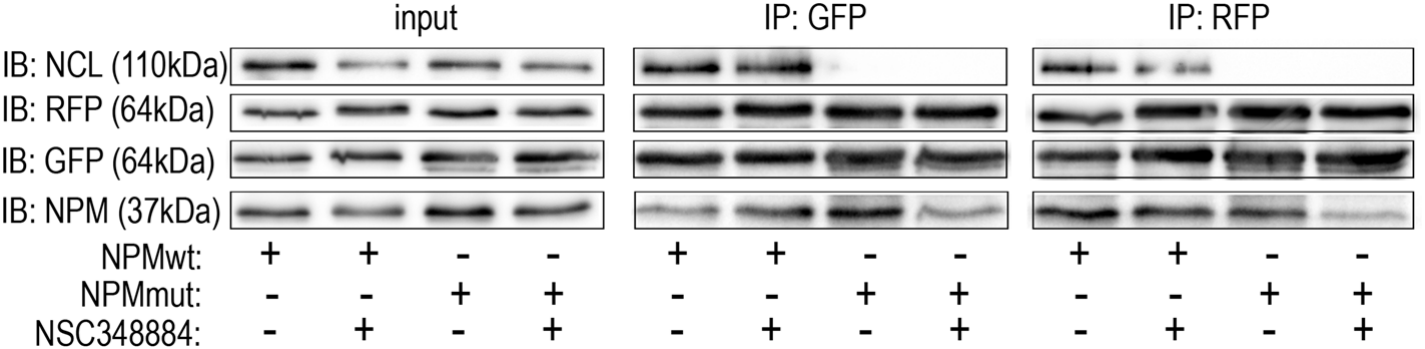
Interaction potential of NPM in NSC348884-treated cells. Immunoprecipitation from 293T cells co-transfected with G_NPMwt + R_NPMwt (NPMwt) or G_NPMmut + R_NPMmut (NPMmut) in control and NSC348884-treated sample (10 µM NSC348884 for 24 h). All exogenous forms as well as the endogenous NPM were detected in all eGFP- and mRFP1-precipitates regardless the NSC348884 addition. Nucleolin (NCL) was detected in NPMwt-precipitates only, independently of the NSC348884 treatment.

The oligomerization of fluorescently labeled NPM was finally tested by the native PAGE and by immunoprecipitation in cell lysates and by FRET in living cells. First, we tested whether the low-MW band attributed to NPM monomers appears in native lysates of 293T cells expressing a combination of G_NPMmut and R_NPMwt after the NSC348884 treatment. Lysate from cells co-expressing G_NPMmut and weakly oligomerizing R_C21F was used to mark the position of the low-MW band (Fig. 10a and Supplementary Fig. S4). No difference between the control and the NSC348884-treated sample was found either under native or semi-native conditions. Similar results were obtained from cells co-transfected with alternative combinations, i.e. with G_NPMwt + R_NPMwt or with G_NPMmut + R_NPMmut (Fig. 10b). Identical samples were afterwards subjected to immunoprecipitation (GFP- and RFP-Trap). All the exogenous NPM forms as well as the endogenous NPM were detected in all GFP- and RFP-precipitates regardless the NSC348884 addition (Fig. 11). In agreement with our previous work^49^, control experiment revealed that NCL co-precipitated with NPMwt and it did not co-precipitate with any form of NPMmut. Again, the NPM-NCL interaction was not affected by the presence of NSC348884 in any experiment.

**Figure 12:**
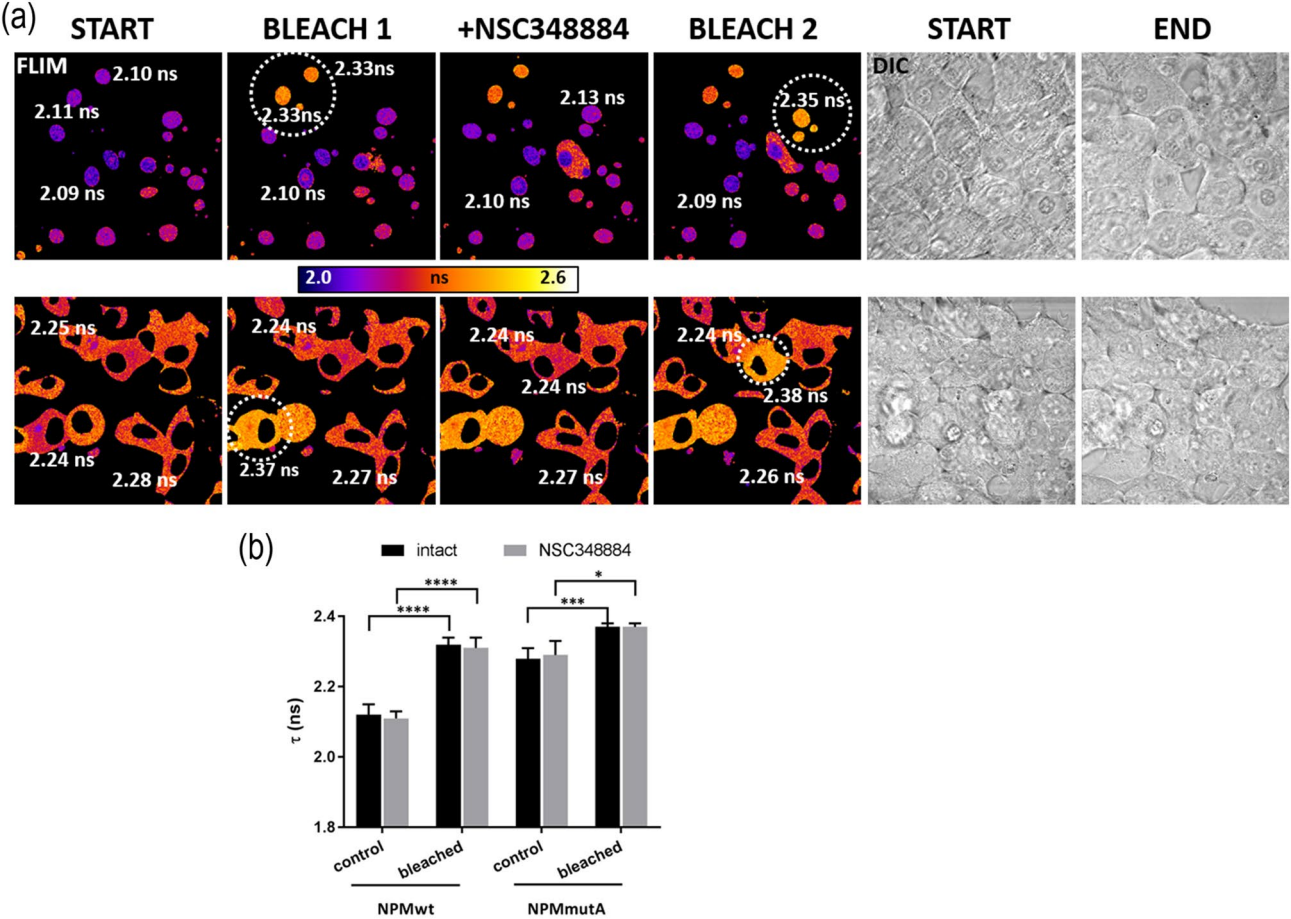
NPM oligomerization after NSC348884 treatment in living cells. (**a**) FLIM-FRET analysis of the eGFP-fluorescence lifetime (*τ*) after 2 h of 10 µM NSC348884 action on cells co-transfected with red and green variants of NPMwt (upper row) or NPMmut (lower row). White numbers: fluorescence lifetime measured in individual cells. Dashed circles: region of mRFP1-photobleaching. Simultaneous cell morphology screening by DIC documents cell rounding induced by NSC348884. (**b**) Statistical evaluation of *τ* values before (control) and after (bleached) the mRFP1 photobleaching in intact (black bar) and NSC348884-treated (grey bar) cells. Student´s t-test of “control” versus “bleached” values: ****p < 0.0001, ***p < 0.001,*p < 0.05.

Oligomerization in living cells was independently tested by the resonance energy transfer. As seen from Fig. 12, FLIM-FRET experiments reveal unchanged eGFP fluorescence lifetime upon NSC348884 treatment of cells co-transfected with donor- and acceptor-labeled NPMwt and NPMmut. Prolonged eGFP-fluorescence lifetime after mRFP1-photobleaching confirmed the complex formation in control cells without NSC348884 (column 1 and 2). The NSC348884-treatment did not affect the lifetime pattern (column 3) and the second round of the acceptor bleaching confirmed persistence of heterooligomers despite the presence of NSC348884 (column 4). Lower FRET extent in the NPMmut co-transfected cells (the second row) is likely a result of lower cytoplasmic NPMmut concentrations and consequent lower complex formation. Nevertheless, presence of FRET is still detected. NSC348884 activity resulting in cell rounding and loss of their contact with the glass surface is visibly documented by the morphology screening during the FLIM experiments (columns 5, 6), similarly to Fig. 9. No lifetime change following mRFP1-photobleaching or NSC348884 treatment was detected in the control sample (Supplementary Fig. S5), i.e. in cells expressing two color variants of NCL, where FRET should not be detected^55^. Altogether, FLIM-FRET experiments confirmed that NSC348884 does not affect NPM oligomerization, although it influences apoptosis and cell adhesion.

**Figure 13:**
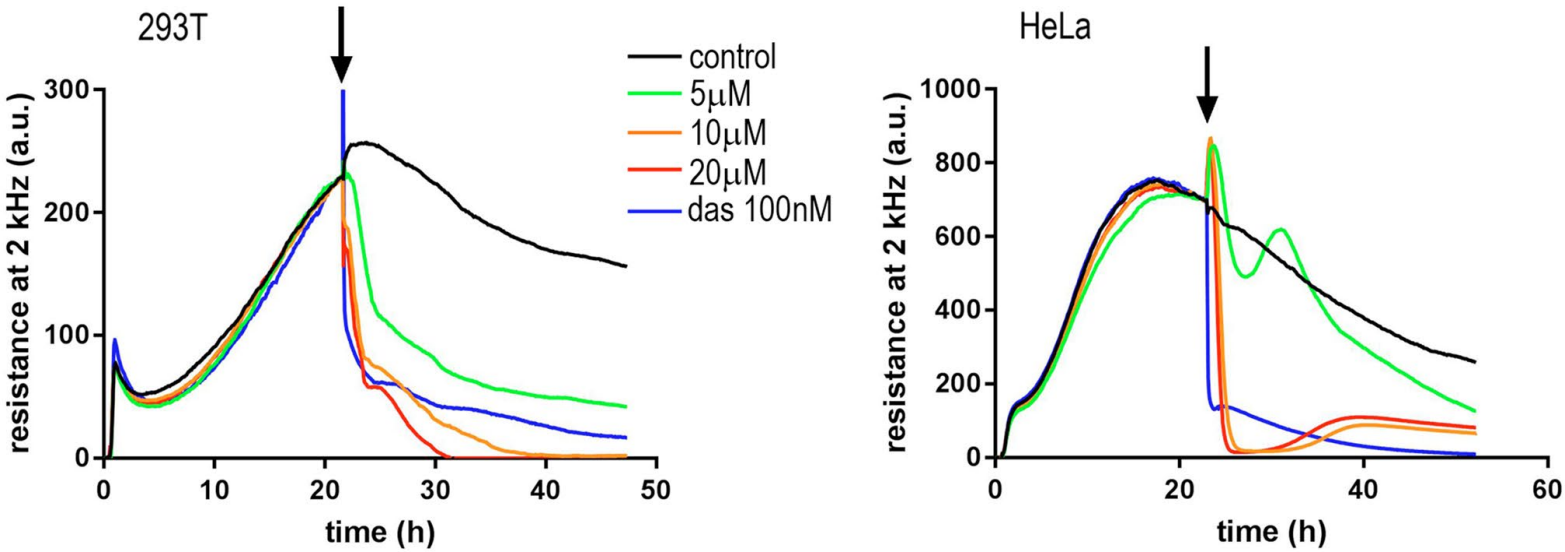
Decrease of the cell-surface contact area after NSC348884 addition. Resistance at 2 kHz was tracked during 293T (left) or HeLa (right) adhesion to the well bottom and after NSC348884 or dasatinib (das) addition. Times of the drug addition are marked by arrows. The curves represent mean values of triplicate/duplicate wells for NSC348884/das, respectively.

**Figure 14:**
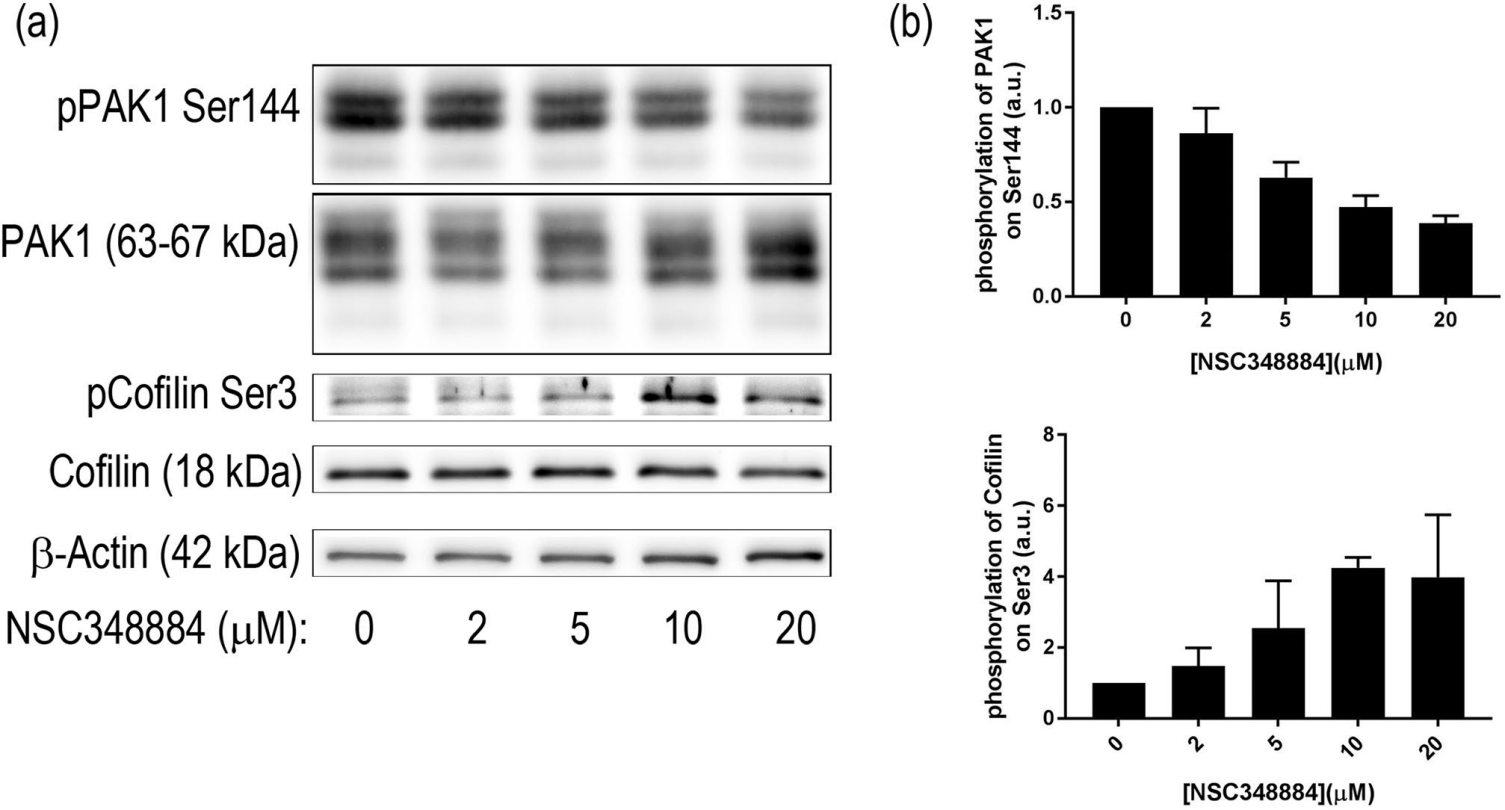
Expression and phosphorylation of adhesion-related protein kinase PAK1 and of the actin regulator Cofilin in 293T cells after 2 h NSC348884 treatment. (**a**) Representative blots, (**b**) summary from 2 experiments. Error bars: ± SD.

To further investigate the mechanism of NSC348884 action, we analyzed changes in the cell-surface contact after NSC348884 addition with help of Electric Cell-Substrate Impedance Sensing (ECIS) technique. ECIS allows for non-invasive real-time monitoring of cell interaction with the surface of the sample well. Small microelectrodes embedded in the bottom of the ECIS plate serve to measure the impedance in a range of frequencies of the sensing electric current, and the signal is then automatically decomposed into resistance and capacitance. In our experiments, the capacitance component at a high frequency (64 kHz) mirrored that of the resistance at 2 kHz and reflected mainly the area of the cell-surface contact. Before the drug addition, the progressive increase of the signal reflects cell attachment and proliferation. With this device, we were able to follow the time course of the adhesivity decrease after NSC348884 addition (Fig. 13). We have reported previously, that inhibition of SRC family kinases by dasatinib resulted in a rapid drop of ECIS signal, which corresponded to cell shrinkage, and we thus used dasatinib as a reference compound^61^. In both adherent cell lines, 293T and HeLa, NSC348884 induced large, dose-dependent changes in the resistance signal, which were similar to those produced by IPA-3^62^, an inhibitor of p21-activated kinases (PAK). PAK are key regulators of adhesion signaling, which have been proposed as therapeutic targets in different kinds of cancer including leukemias^63,64^. We thus analyzed possible effect of NSC348884 on expression and activity of PAK1, as well as of Cofilin, which governs actin remodeling during changes of cell shape. Indeed, Ser144 phosphorylation of PAK1 reporting on its kinase activity was reduced after 2 h of NSC348884 treatment whereas total PAK1 expression remained unchanged (Fig. 14). Simultaneously, inactivating phosphorylation at Ser3 of Cofilin was detected.

## Discussion

The N-terminal region of NPM is essential for its oligomerization as well as for its chaperone function as numerous proteins interact with NPM through this domain. Since AML-related NPM mutation does not substantially affect its ability to form oligomers, NPM-interacting proteins become frequently mislocalized together with aberrantly localized NPMmut. Targeting the NPM oligomerization offers a possibility to manipulate localization of the interacting partners. Simultaneously, profiting from slightly different oligomerization properties of NPMmut and NPMwt, a fine control of NPM oligomerization by appropriate concentrations of oligomerization-inhibiting drugs might have a therapeutic effect in the AML with *NPM1* mutation. Although several alterations of NPM N-terminal domain were reported to disrupt NPM oligomerization in vitro, results thoroughly describing NPMwt and NPMmut oligomerization in vivo are missing. We have previously documented that C21 point mutations do not disrupt NPM oligomerization in living cells^55^. Here we demonstrate that the cytoplasmic mislocalization of R_C21 and R_NPMwt in cells co-expressing G_NPMmut is very similar (Fig. 1). This strongly indicates an existence of interaction between natural NPM forms and the C21 mutants. Our results from native and semi-native electrophoreses allowed us to evaluate the aggregation potential of C21 mutants depending on the substituting aminoacid (Fig. 2) in vitro, in agreement with the results of Prinos et al.^54^ obtained mainly in experiments with recombinant proteins. Whereas C21F substitution resulted in disruption of NPM oligomers under native conditions (Fig. 3 and Supplementary Fig. S1), the effect of the C21A substitution was only detectable under reducing conditions. NPM oligomers were reported to consist of five NPM molecules^30^, and formation of heterooligomers containing NPMwt and NPM mutants were found to be highly frequent^35,36^. We therefore suggest that the in vivo stability of NPM heterooligomers is permitted by a sufficient number of NPMwt molecules in the heterooligomeric complex. Thus, in living cells, the C21 point mutations do not have potential to fully disrupt these complexes, although their ability to retain in oligomers is compromised under in vitro conditions.

In agreement with results of Enomoto et al.^56^, partial or complete deletion of NPM oligomerization domain (aa1-117) led to delocalization of the truncated protein from the nucleoli to the nucleoplasm (Fig. 4). However, even the NPM with completely deleted N-domain (Δ117) exhibited higher concentration in the nucleoli compared to the nucleoplasm. This is likely due to the fact, that nucleolar localization signal as well as nucleic acid binding domains remain unaffected by the deletion. We have found that oligomerization of the deletion mutants was completely abrogated and no interaction between two truncated NPM forms was detected (Fig. 5 and Supplementary Figs. S2, S3). Nevertheless, immunoprecipitation revealed presence of both exo- and endogenous NPMwt in the G_Δ117 precipitates indicating that the truncated NPM yet participates in complexes that are possibly too large to enter the native gel. The existence of mixed Δ117/NPMwt complexes is also supported by the FRET results monitoring the Δ117-NPMwt interaction in living cells (Fig. 6). Enhanced level of nucleolar proteins NCL and FBL co-precipitated with the deletion mutants suggests better accessibility of the NPM region responsible for binding of these proteins. Both NCL and FBL were previously found to reside in the nucleoli of cells with NPM mutation^46,49,59^ and they may therefore mediate the nucleolar localization of the deletion mutants. Complexes containing NCL and/or FBL together with NPMwt and Δ117mutants thus represent a potential pool of proteins that can co-precipitate with the deletion mutants.

NSC348884 is declared to inhibit NPM oligomerization^50^. Its structure was obtained by in silico screening using a small molecular library. As the crystal structure of the human NPM was not available, *Xenopus* NO38-core chaperone structure (residues 1–107) was used for the screening. The sequence identity of this structure with equivalent part of human NPM oligomerization domain is 77%. NSC348884 was identified as the best candidate for inhibition of the dimerization interface of this polypeptide. Authors of the original paper^50^ used native electrophoresis to demonstrate NSC348884-induced oligomer disruption in LNCaP and HCT116 cell lines. They detected diminished bands near 121 kDa, which they attributed to NPM oligomers. Surprisingly, the intensity of monomer bands remained unchanged. Other complex phenomena like induction of apoptosis and p53 upregulation were also found in NSC348884-treated cells^41,50^. From the available data it seems that function of NSC348884 as an inhibitor of the full-length human NPM oligomerization was not unequivocally proven. We therefore analyzed effect of NSC348884 on various leukemia cell lines and on cells expressing fluorescently labeled NPM constructs. First, we tested cell viability and apoptotic signatures in order to determine the range of proper NSC348884 concentrations for the live-cell experiments. Concentrations required for a substantial viability decrease and caspase-3 fragmentation fell into the interval of 2–10 µM for the majority of the cell lines (Fig. 7). Neither the OCI-AML3 cell line nor the primary cells of AML patients with NPM mutation displayed enhanced sensitivity to NSC348884 treatment. Consistently with previous results^41,50^, NSC348884 induced p53 upregulation in some cell lines. We also noticed NSC34884-induced downregulation of p14Arf in 293T and HeLa cells (data not shown). In contrast to results of Balusu et al^41^, none of the tested cell lines nor primary cells from AML patients displayed any change in the NPM oligomerization upon treatment with efficient NSC348884 concentrations when investigated by the native and semi-native electrophoreses (Fig. 8). Similarly, oligomers containing fluorescently labeled NPMwt and NPMmut were not affected by the NSC348884 treatment (Fig. 10 and Supplementary Fig. S4). These results were further verified by immunoprecipitation where both exogenous and endogenous NPM co-precipitated with both GFP- and RFP-labeled NPMwt and NPMmut, despite the presence of NSC348884 (Fig. 11). Also the fluorescence microscopy revealed sustained fraction of NPMwt in the cytoplasm of NSC348884 treated cells co-expressing NPMwt and NPMmut, which witnesses for their interacton. Accordingly, the FLIM-FRET proved persisting interaction between fluorescently labeled NPM molecules upon the NSC348884 treatment (Fig. 12). Cells expressing two fluorescent variants of likely noninteracting NCL were used as a control (Supplementary Fig. S5). As expected, NCL molecules labeled with the eGFP donor and the mRFP1 acceptor on their N-termini did not exhibit any FRET, which was proved by a zero lifetime change upon the acceptor photobleaching. The result was independent of the NSC348884 treatment. Compared to NCL, the presence of FRET in the cells with fluorescently labeled NPM is clearly detectable both before and after the NSC348884 treatment. We conclude that contrary to the published data^50^, NSC348884 does not act as an oligomerization inhibitor and does not affect formation of NPM oligomers under physiological conditions. This finding is extremely important in view of the fact that this drug has been recently reported to cause numerous cellular effects, which were ascribed, in accordance with its declared function, to disruption of NPM oligomerization^51–53^.

During the live-cell experiments, we noticed apparent changes in cell adhesivity. The cell-surface contact area during the NSC348884 treatment was therefore monitored by Electrical Cell-Substrate Impedance Sensing (ECIS) (Fig. 13). The rapid onset of changes in the ECIS signal indicated that the cell shrinkage and detachment was not a secondary effect accompanying apoptosis. As the course of the ECIS signal was similar to that induced by the inhibitor of p21-activated kinases, IPA-3^62^, we investigated also activity and expression of PAK1 and Cofilin, a known actin regulator^63^ (Fig. 14). The observed changes of both PAK1 and cofilin phosphorylation indicate that NSC348884 interferes with adhesion signaling. Further research is required to elucidate mechanistic role of NSC348884 in this process and its potential for anticancer therapy.

## Conclusion

We have shown that a proposed inhibitor of NPM oligomerization, NSC348884, does not affect NPM oligomer formation in any of the examined leukemia cells. Moreover, the cell sensitivity to NSC348884 treatment is not potentiated by AML-associated NPM mutation. On the other hand, we have uncovered so far unknown effect of NSC48884 on the cell-surface adhesion, which could play a key role in the complex cellular response to the NSC48884 treatment.

In addition, our findings prove that point mutations in Cysteine 21 slightly potentiate oligomer dissociation but the overall NPM interaction potential with other NPM molecules remains conserved in living cells. Deletion mutants lacking part of the NPM N-terminal domain completely lose their oligomerization ability, but they partially retain the interaction with NPMwt, possibly through enhanced interaction with other nucleolar proteins in complexes with NPMwt.

## Material and methods

All methods were carried according to Declaration of Helsinki.

### Cell culture and chemicals

Leukemia cell lines MV4-11, OCI-AML2, OCI-AML3, KG-1, and KASUMI-1 were purchased from DSMZ (Germany), HL-60 were from ECACC (GB), adherent cell lines HEK-293T and HeLa were kindly provided by dr. Š. Němečková (Department of Immunology, Institute of Hematology and Blood Transfusion) and dr. J. Malínský (Institute of Experimental Medicine, Czech Academy of Science), respectively. The cells were cultivated in growth media with fetal bovine serum (FBS), glutamine and antibiotics (all from Sigma-Aldrich) according to manufacturers recommendation: MV4-11, KG-1, HL-60, and HeLa in RPMI-1640/10% FBS, OCI-AML2 and OCI-AML3 in alpha-MEM/20% FBS, KASUMI-1 in RPMI-1640/20% FBS and HEK-293T in DMEM/10% FBS. Peripheral blood mononuclear cells (PBMC) originated from leukapheresis of hyperleukocytic AML patients. PBMC were separated using Histopaque 1077 (Sigma-Aldrich), washed with PBS and resuspended in RPMI-1640 with 10% FBS. The presence of C-terminal NPM mutation was detected by PCR and the mutation type was determined by sequencing^26^ and confirmed by Western blotting and immunofluorescence using specific anti-NPMmut antibody^49^. All patients signed informed consent to the use of their biological material for research purposes in agreement with the Declaration of Helsinki. The study has been approved by the Ethics Committee of the Institute of Hematology and Blood Transfusion of the Czech Republic. All cells were cultivated in 5% CO_2_ atmosphere at 37 °C. Stock solution of 10 mM NSC34884 was added to cell suspensions to final concentrations and times as indicated in the Results section.

### Plasmid construction and transfection

As we described in detail previously^36,59^, the gene for NPM was amplified from cDNA library (Jurkat cells, Origene) by PCR and inserted to vectors peGFP-C2 and pmRFP1-C2 (originally Clontech), designed for expression of protein chimeras with a fluorescent protein connected to the N-terminus of the target protein, by standard methods of molecular cloning. NPM mutants were constructed by PCR using extended primers targeting NPM1 sequence neighboring regions cut from the N-terminus or containing the mutated part of the exon 12 of the *NPM1* gene complemented with appropriate restriction sites^36^. After amplification in *E. coli*, the plasmids with subcloned genes were purified with PureYield Plasmid Miniprep System (Promega) and transfected into adherent cell lines using jetPRIME transfection reagent (Polyplus Transfection).

### Cell lysis and western blotting

*Cell lysis* As described previously^49^, cells were washed with PBS and lysed depending on the intended application. For direct use in SDS-PAGE, the cells were lysed in Laemmli sample buffer (SB, 50 mM Tris pH 6.8, 2% SDS, 100 mM DTT, 10% glycerol), boiled at 95 °C for 5 min, centrifuged at 200.000 g/4 °C for 4 h and the supernatant was stored at − 20 °C. For other applications, the cells were lysed in Lysis buffer (LB, 10 mM Tris/Cl pH7.5, 150 mM NaCl, 0.5 mM EDTA, 0.5% NP-40, protease and phosphatase inhibitors) for 30 min/4 °C, centrifuged at 20.000 g/4 °C for 10 min and supernatant was mixed 1:1 with the appropriate buffer.

*Native and semi-native PAGE* Lysates were mixed with 2xnative buffer (NB, 50 mM Tris pH6.8, 10 mM DTT, 10% glycerol) and subjected without boiling to 7.5% AA Tris-glycin gel without SDS for native electrophoresis, or to the gel with SDS (2%) for semi-native electrophoresis.

*Western blotting* Five to ten microliters of each sample were subjected to native or SDS-PAGE and transferred into PVDF or nitrocelulose membrane (BioRad). Mouse monoclonal antibodies against β-actin, GFP, dsRed, NCL, FBL, NPM (clone 3F291 for NPMwt + mut detection, clone E3 for NPMwt detection), and p14Arf were from Santa Cruz Biotechnology. All mouse primary antibodies were used at a dilution 1:100–1:500. Rabbit polyclonal antibody against NPMmut (pab50321, Covalab) was used at 1:2000 dilution. Rabbit monoclonal anti-PAK1 (1:2000, Abcam) and anti-PAK1-pSer144 (1:20,000, Abcam) and rabbit polyclonal antibodies and anti-Cofilin and anti-Cofilin-pSer3 (Cell Signalling Technology) were used for adhesion-related protein detection. Anti-mouse and anti-rabbit HRP-conjugated secondary antibodies were purchased from Thermo Scientific and used at concentrations 1:10.000–1:50.000. ECL Plus Western Blotting Detection System (GE Healthcare) was used for chemiluminescence visualization and evaluation by G-box iChemi XT4 digital imaging device (Syngene Europe). Alternatively, Alexa488-conjugated anti-rabbit and Alexa647-conjugated anti-mouse secondary antibodies (ThermoFisher) for simultaneous detection of NPMmut and NPMwt + mut were used.

### Immunoprecipitation

Immunoprecipitation using GFP- or RFP-Trap (Chromotek) was performed according to manufacturer´s instruction as described previously^36^. Briefly, cells were harvested and washed with PBS, lysed in LB for 30 min/4 °C and centrifuged at 20.000 g/4 °C for 10 min. The lysate was mixed with GFP/RFP-nanobeads and rotated for 1 h/4 °C. The beads were extensively washed with diluting buffer (10 mM Tris/Cl pH7.5, 150 mM NaCl, 0.5 mM EDTA), resuspended in SB, boiled at 95 °C for 10 min and centrifuged 20.000 g/4 °C for 10 min. Supernatant was stored at − 20 °C until used for SDS-PAGE.

### Live-cell imaging

The cells were seeded in the 2.5 mm culture dish with glass bottom (Cellvis) for 24 h and then transfected with plasmids containing fluorescent variants of the desired genes. After another 24 h, the transfected cells were observed under confocal laser scanning microscope FluoView FV1000 (Olympus Corporation) using 543 nm excitation for RFP fluorescence and 488 nm excitation for GFP and for differential interference contrast (DIC) observation. UPlanSAPO 60 × NA1.35 oil-immersion objective was used for imaging. For long-term monitoring, the culture dish was sealed by parafilm to prevent CO_2_ leakage and it was placed into microscopy chamber tempered to 37 °C. NSC348884 was added just before the start of the measurement. Fluorescence images were processed by the FluoView software FV10-ASW 3.1.

### Lifetime imaging and acceptor bleaching

The apparatus used for lifetime imaging is described in detail elsewhere^65^. Briefly, we used inverted IX83 microscope equipped with a FV1200 confocal scanner (Olympus, Germany), cell cultivation chamber (Okolab) and FLIM add-ons from PicoQuant. Fluorescence was excited by a pulsed diode laser (LDH-DC-485, 485 nm, PicoQuant) running at 20 MHz repetition rate. Light was coupled to the microscope by a single-mode optical fiber and reflected to the sample by 488 nm long-pass dichroic mirror (Olympus). Typically, UPLSAPO 60XW NA 1.2 water-immersion objective (Olympus) was used for imaging. Fluorescence was directed via multimode optical fiber to a cooled GaAsP hybrid PMT (PicoQuant) through the 520/35 bandpass filter (Semrock). Signal was processed by the TimeHarp 260-PICO TCSPC card and the SymPhoTime64 software (both PicoQuant). To avoid pile-up artifacts, the data collection rate at brightest pixels was kept below 5% of the excitation frequency. FLIM images were collected in a few minutes with the excitation power around 0.1 μW. Acceptor photobleaching was done by a 561 nm semiconductor CW laser with a multi-mW power at the focal point. All experiments were done at 37 °C.

### Lifetime data processing

Lifetime images were generated in the SymPhoTime64 by the “fast-FLIM” method when pixel lifetimes were calculated by a method of moments^66^. Specifically, pixel lifetimes *τ*_*avg*_ were determined as the difference between the barycenter of the fluorescence decay and the time-offset *t*_*offset*_ of the steepest growth of the decay at each pixel:

1 $$\tau_{avg} = {\sum I_{i}t_{i}}/{\sum I_{i} - t_{offset}}$$

where *I*_*i*_ is a decay intensity at time *t*_*i*_. Exported FLIM images were further processed and visualized by the Fiji software^67^. An accurate analysis of the cumulative decays from larger area of interest was done by the least-squares reconvolution also in the SymPhoTime64. Fluorescence was assumed to decay multiexponentially according to the formula:

2 $$I(t) = \sum\limits_{i}\alpha_{i}\cdot {\rm exp}(-t/\tau_{i}0), \quad \sum\limits_{i} \alpha_{i}=1$$

where *τ*_*i*_ and *α*_*i*_ are the fluorescence lifetimes and the corresponding amplitudes, respectively. Typically, 2 decay components were sufficient for acceptable fit. The intensity-weighted mean fluorescence lifetime was calculated as:

3 $$\tau_{mean} = \sum\limits_{i}f_{i}\tau_{i}=\sum\limits_{i} \alpha_{i}\tau_{i}^{2}/\sum\limits_{i} \alpha_{i}\tau_{i}$$

where *f*_*i*_ are fractional intensities of the *i*th lifetime component:

4 $$f_{i} =  \alpha_{i}\tau_{i}/\sum\limits_{i} \alpha_{i}\tau_{i}, \quad \sum\limits_{i} f_{i}=1,$$

### Electrical cell-substrate impedance sensing (ECIS)

Impedance measurements were performed using the ECIS Zθ device (Applied Biophysics). The wells of 8W10E+ plates were filled with 200 µl culture medium and the baseline was monitored for several hours before cell addition. HeLa or 293T cells were seeded at 120.000 cells/well and monitored overnight, the inhibitors were added after 20–24 h. One well from each plate was left empty (medium only), and the signal from this well was used as the baseline for the other wells of the same plate. The instrument automatically decomposes the impedance signal into resistance and capacitance. As the course of capacitance at 64 kHz mirrored that of resistance at 2 kHz, the observed evolution of the resistance signal reflects changes in the cell-surface contact area. The ECIS records were exported to Microsoft Excel and processed using the GraphPad Prism software: the background was set to zero at a time point shortly before cell seeding, and the baseline (empty well) was subtracted. The curves shown in the graphs represent the averages from replicate wells, which were run in parallel.

### Statistical analyses

As described in our previous work^36^, the majority of experiments were performed using cell lines and repeated until the observed differences between groups reached statistical significance. A *p* value of 0.05 or lower was pre-set to be indicative of a statistically significant difference between groups compared. In diagrams, arithmetic means of replicates of all experiments were plotted with SD error bars. Significance levels (*p* values of ANOVA or Student’s t-test) were determined using InStat Software (GraphPad Software).

## Supplementary Information

Supplementary information.

## Abbreviations

NPMwt: Wild-type nucleophosmin
NPMmut: Nucleophosmin with AML-associated mutation type A
C21: Cysteine 21 in nucleophosmin
C21A: Substitution of C21 to alanine
C21F: Substitution of C21 to phenylalanine
Δ25: Nucleophosmin with deletion of the first 25 amino acids
Δ100: Nucleophosmin with deletion of the first 100 amino acids
Δ117: Nucleophosmin with deletion of the first 117 amino acids
G_: EGFP-labeled protein at the N-terminus
R_: MRFP1-labeled protein at the N-terminus
