# Supplementary material for: NSC348884 cytotoxicity is not mediated by inhibition of nucleophosmin oligomerization
Source: Sci Rep. 2021 Jan 13;11:1084. doi: 10.1038/s41598-020-80224-1 (PMC7806638; doi:10.1038/s41598-020-80224-1)
Supplement: Supplementary file 1 — Supplementary information. [file 41598_2020_80224_MOESM1_ESM.docx]

**NSC348884 cytotoxicity is not mediated by inhibition of nucleophosmin oligomerization**

Markéta Šašinková^1^, Petr Heřman^2^, Aleš Holoubek^1^, Dita Strachotová^2^, Petra Otevřelová^1^, Dana Grebeňová^1^, Kateřina Kuželová^1^, Barbora Brodská^1^

**Supplementary information 1: Supporting figures**


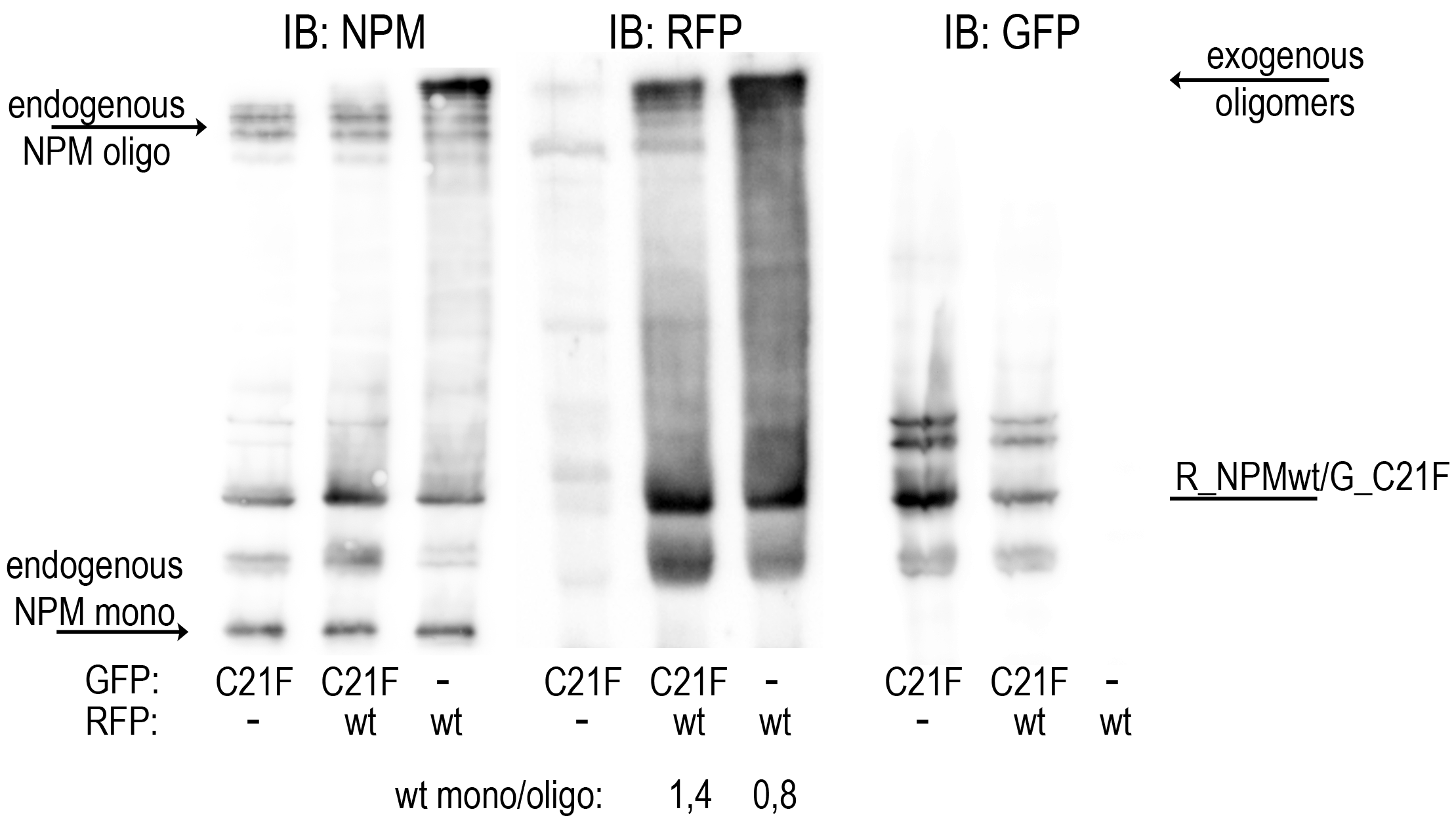


**Fig. S1: Effect of C21F substitution on the stability of heterooligomers with NPMwt.** Western blots of semi-native PAGE of samples from 293T cells transfected with R_NPMwt (wt), G_C21F (C21F) or with their combination. Similar results were obtained with the inverse labeling combination, i.e. with G_NPMwt and R_C21F.





**Fig. S2: Significance of the N-terminus for NPM oligomerization.** Semi-native PAGE of 293T cells co-transfected with various combinations of ∆117 and NPMwt illustrates absence of interaction between Δ117 and NPMwt.


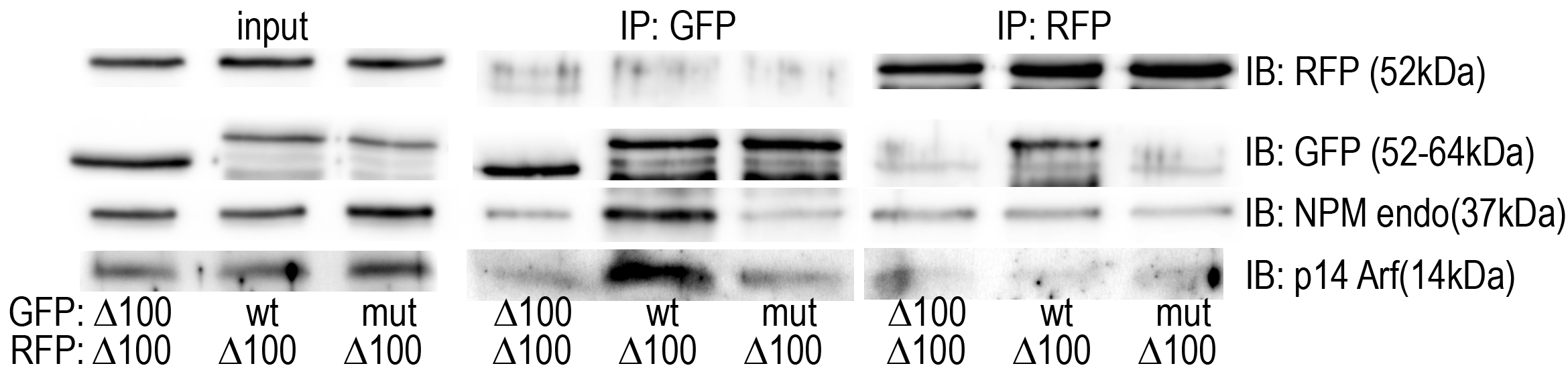


**Fig. S3:** **Significance of the N-terminus for NPM oligomerization.** Lysates from 293T cells co-expressing GFP-labeled ∆100/NPMwt/NPMmut and RFP-labeled Δ100 were subjected to GFP- and RFP-immunoprecipitation. The levels of co-precipitated interaction partners were investigated.





**Fig. S4:** **Native (upper row) and semi-native (lower row) PAGE from 293T cells transfected with various fluorescent variants of NPM and treated with 10μM NSC348884 for 24h.** Cells co-transfected with G_NPMmut and R_NPMwt (lanes 1 and 2) or R_C21F (lane 3). G_NPMmut and R_NPMwt/C21F are detected in the untreated (lanes 1 and 3) or NSC348884-treated cells (lane 2) by anti-GFP and anti-RFP antibodies.


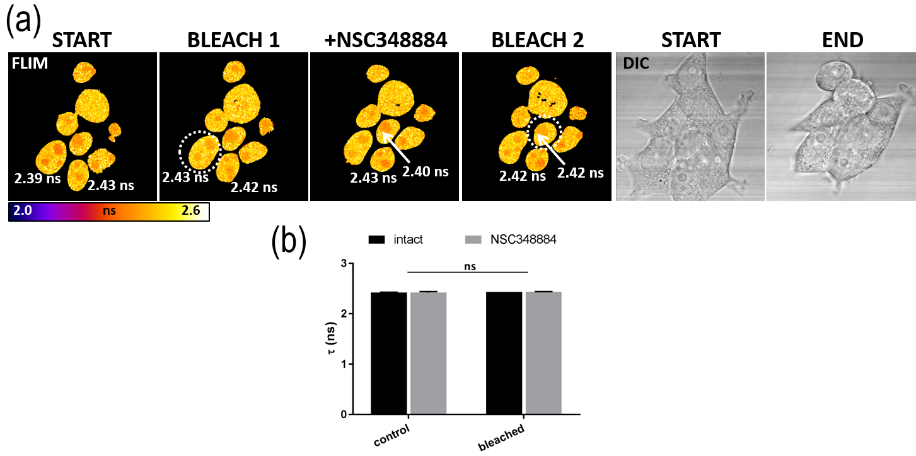


**Fig. S5: NCL expression in live cells after NSC348884 treatment.** (a) FLIM-FRET analysis of eGFP-fluorescence lifetime (τ) during 2h of 10μM NSC348884 action on cells co-transfected with donor- and acceptor-labeled variants of NCL. White numbers: τ (ns) measured in individual cells. Dashed circles: region of the acceptor (mRFP1) photobleaching. Simultaneous cell morphology screening by DIC documented cell rounding induced by NSC348884. (b) Statistical evaluation of τ before (control) and after (bleached) mRFP1-photobleaching in intact (black bars) and NSC348884-treated (grey bars) cells. Student´s t-test of the “control” vs. “bleached” values: not significant.

**Supplementary information 2: full-length blots**

**Fig. 5:**

(b):

NPM oligo&mono:
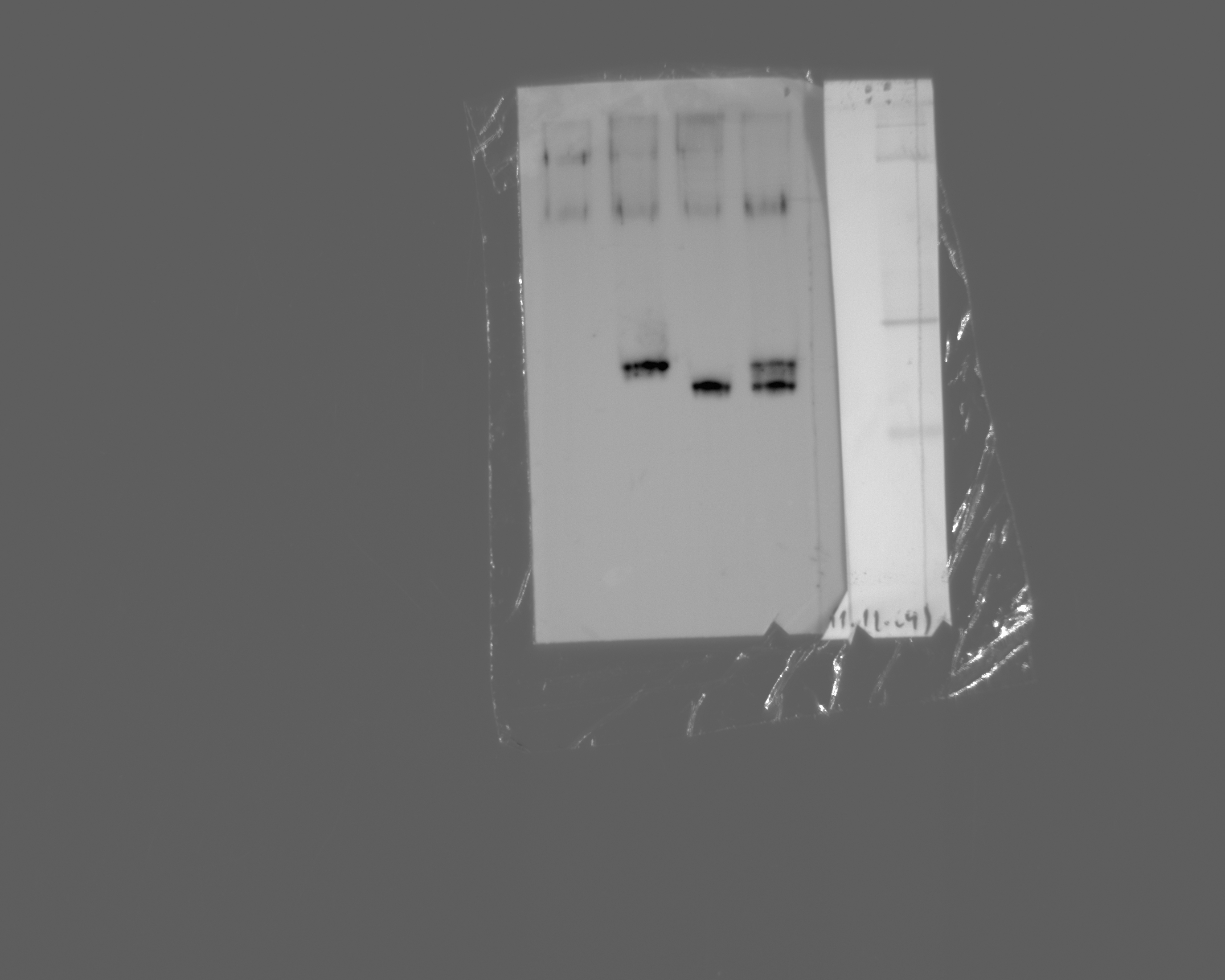
 GFP mono:
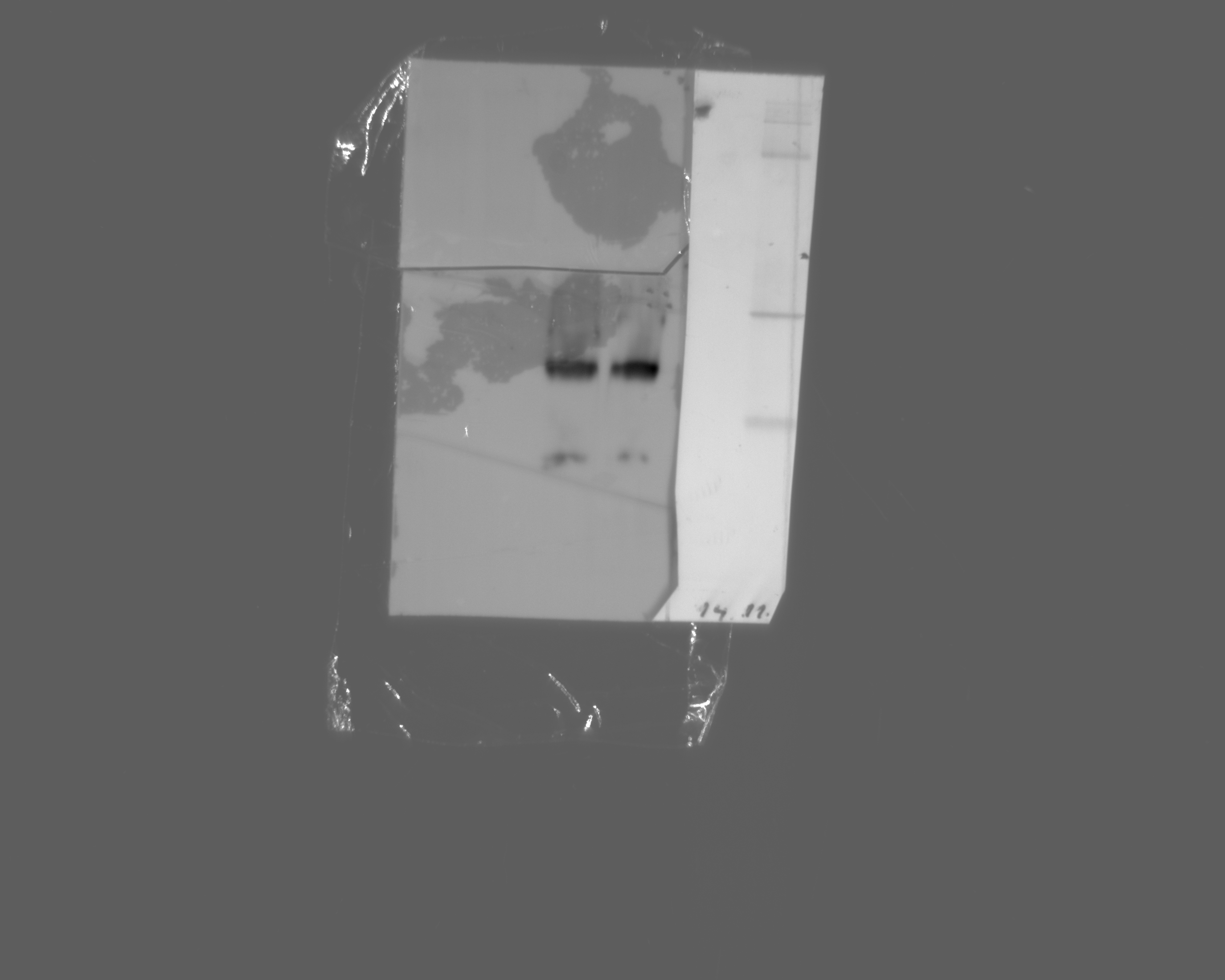


wt

wt

wt

Δ117

Δ117

wt

Δ117

Δ117

Δ117

Δ117

Δ117

wt

wt

Δ117

wt

wt

GFP_Δ117 mono

GFP mono

RFP/GFP_wt oligo

RFP_Δ117 mono

endo oligo

GFP oligo&RFP mono:
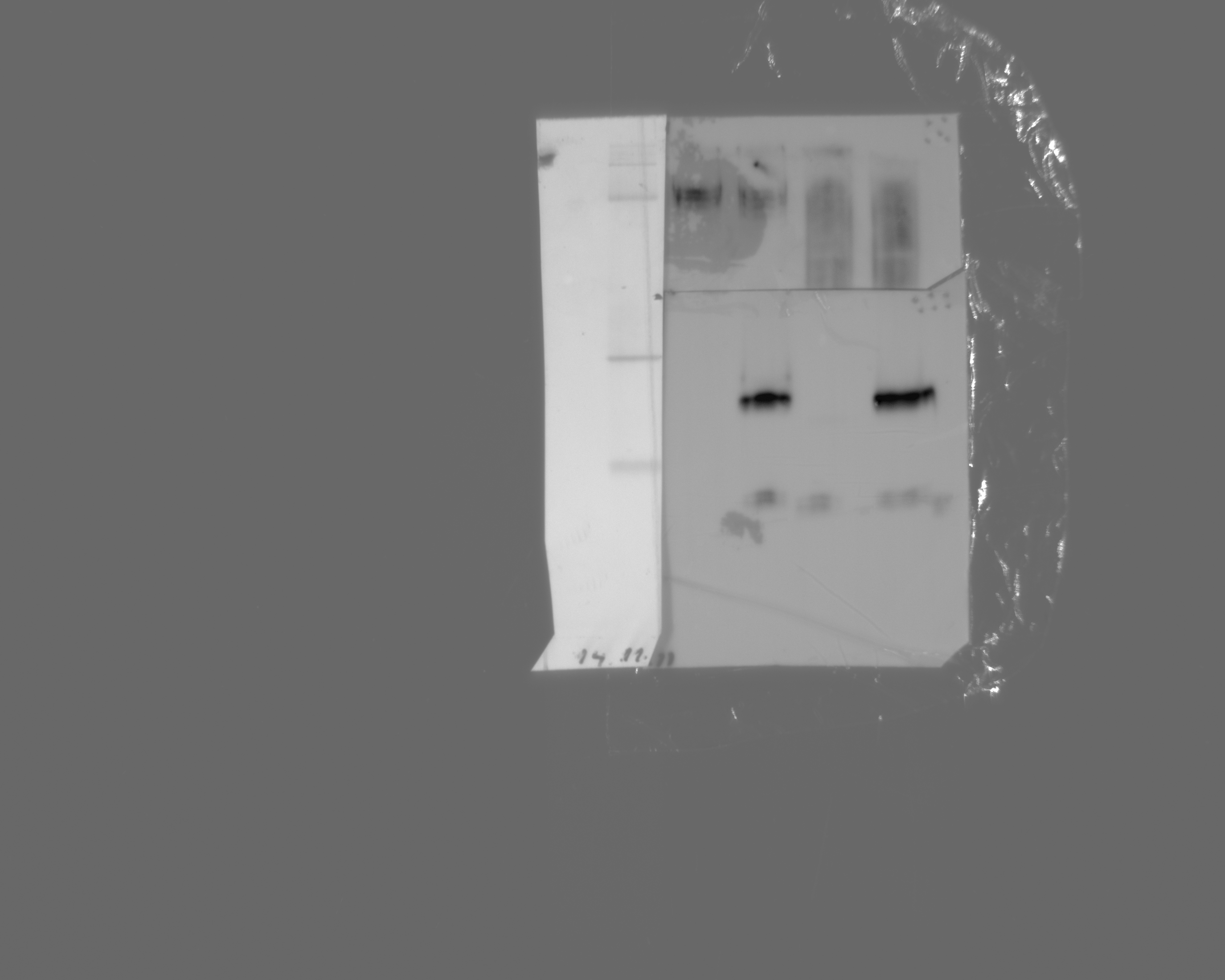
 RFP oligo:
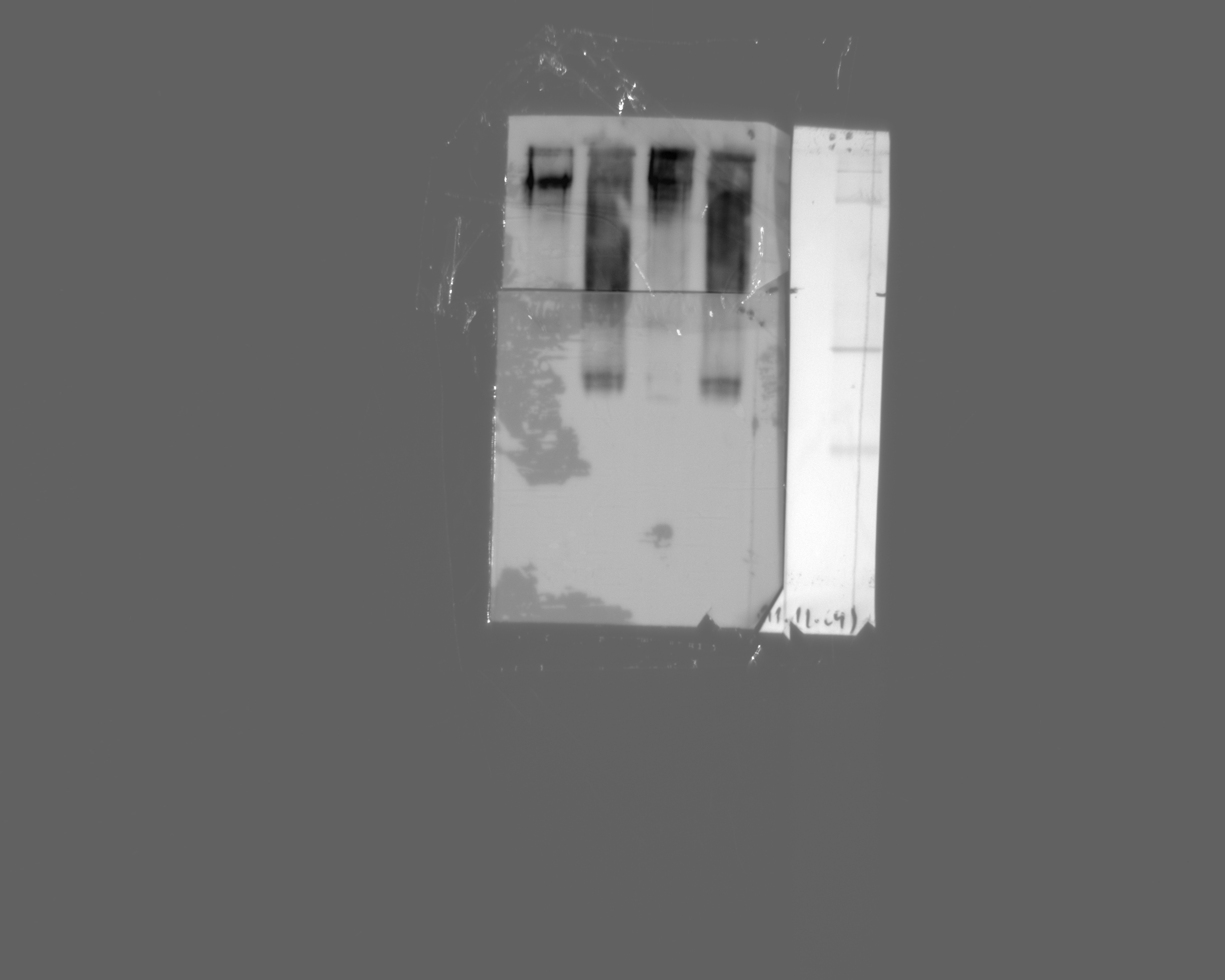


Δ117

Δ117

Δ117

wt

wt

Δ117

wt

wt

wt

wt

wt

Δ117

Δ117

wt

Δ117

Δ117

RFP mono

RFP oligo

RFP mono

GFP oligo

(c):

NCL&FBL Input:
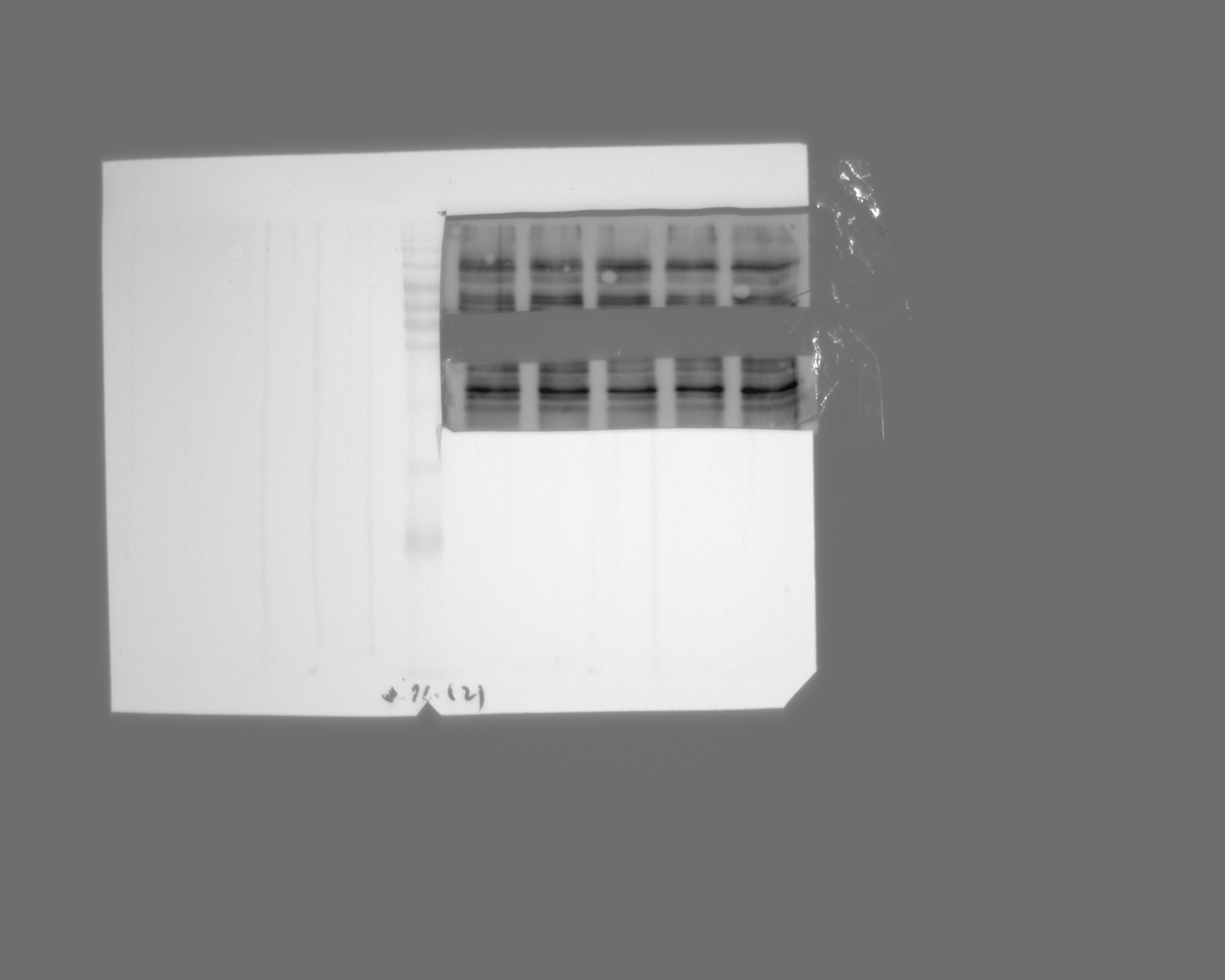
 NPM Input:
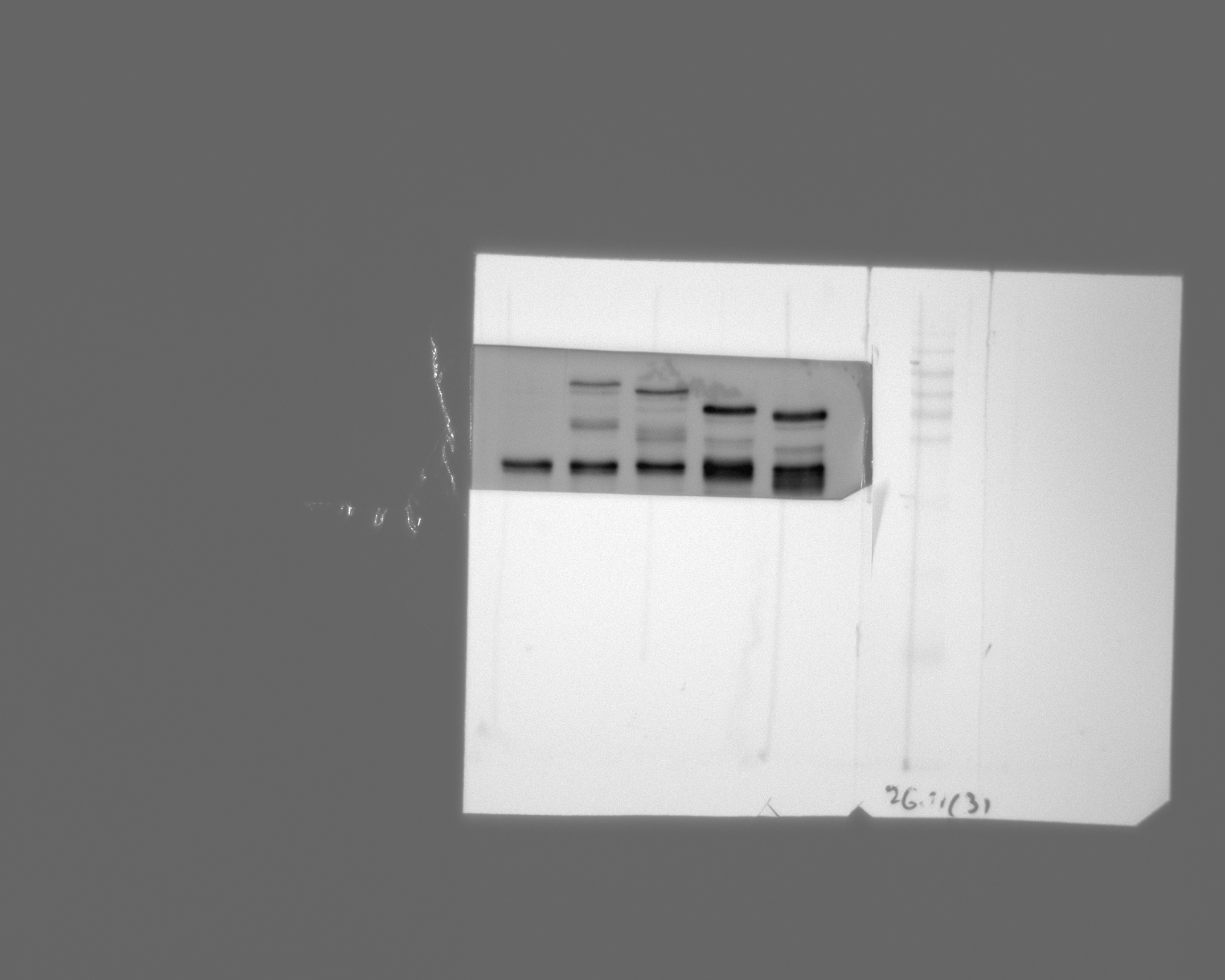


NPM exo

free

GFP

Δ117

Δ100

Δ25

wt

Δ100

free

GFP

Δ25

Δ117

NPM endo

FBL

NCL

wt

p14Arf Input:
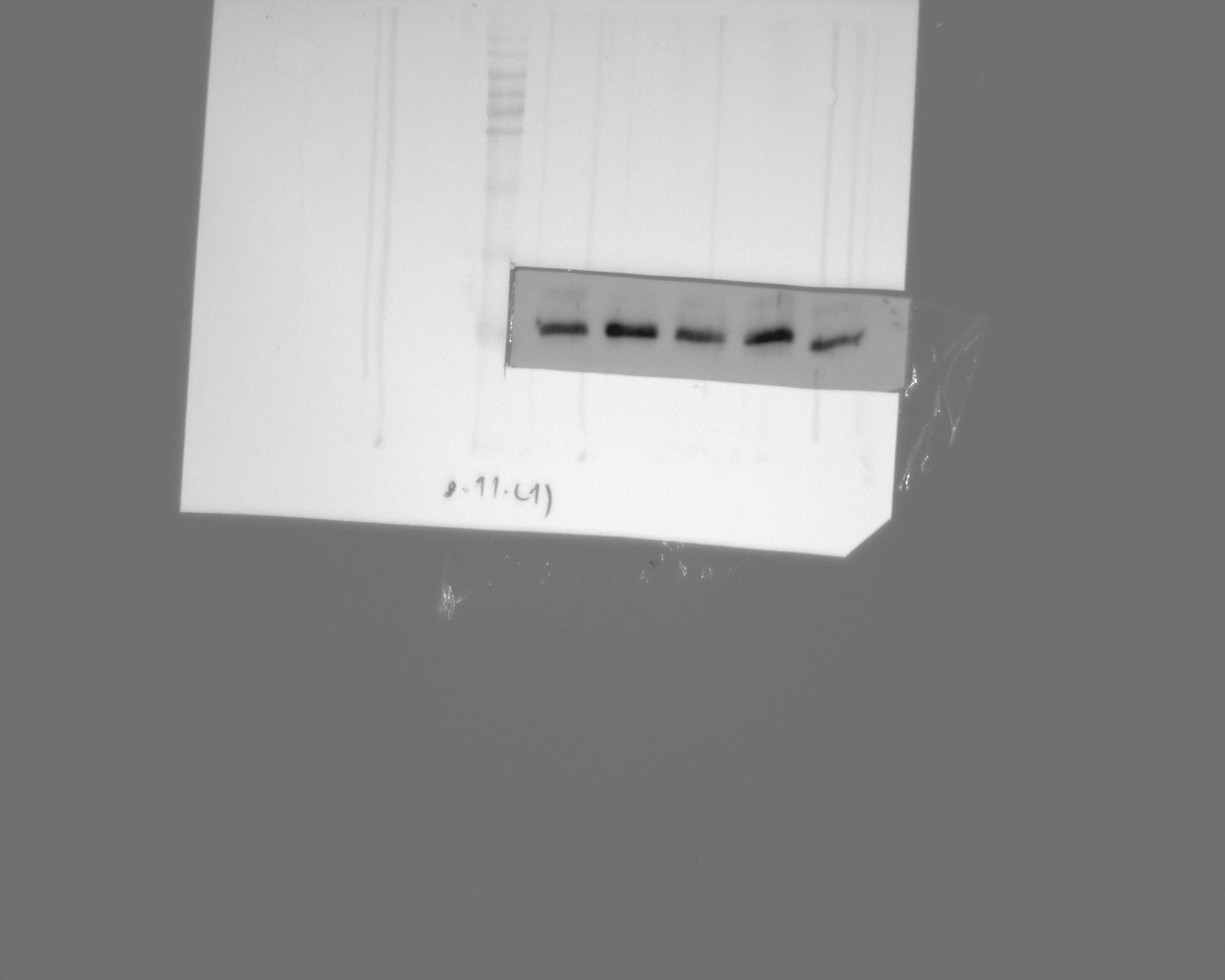


p14Arf

free

GFP

Δ117

Δ100

Δ25

wt

NCL IP:
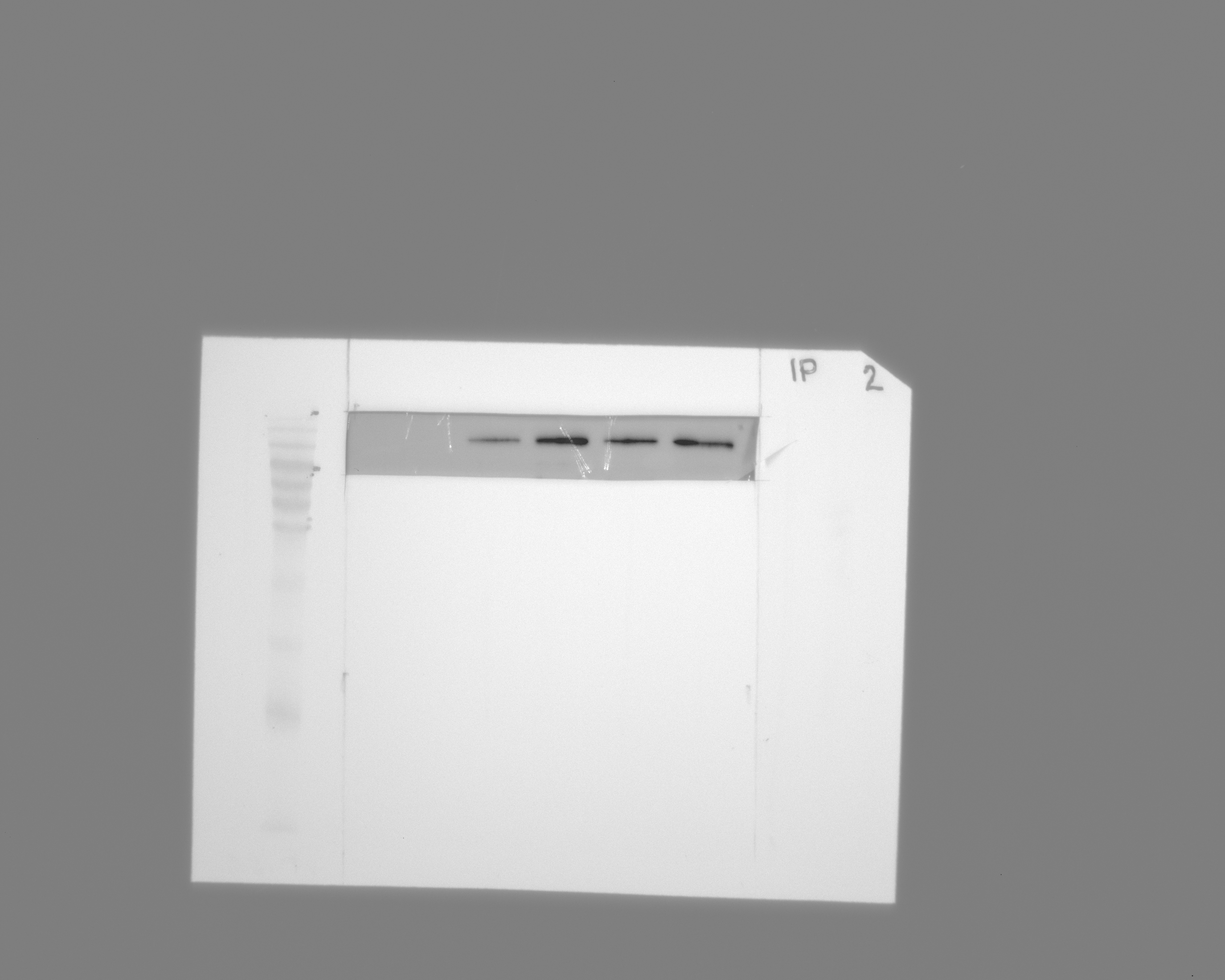
 FBL IP:
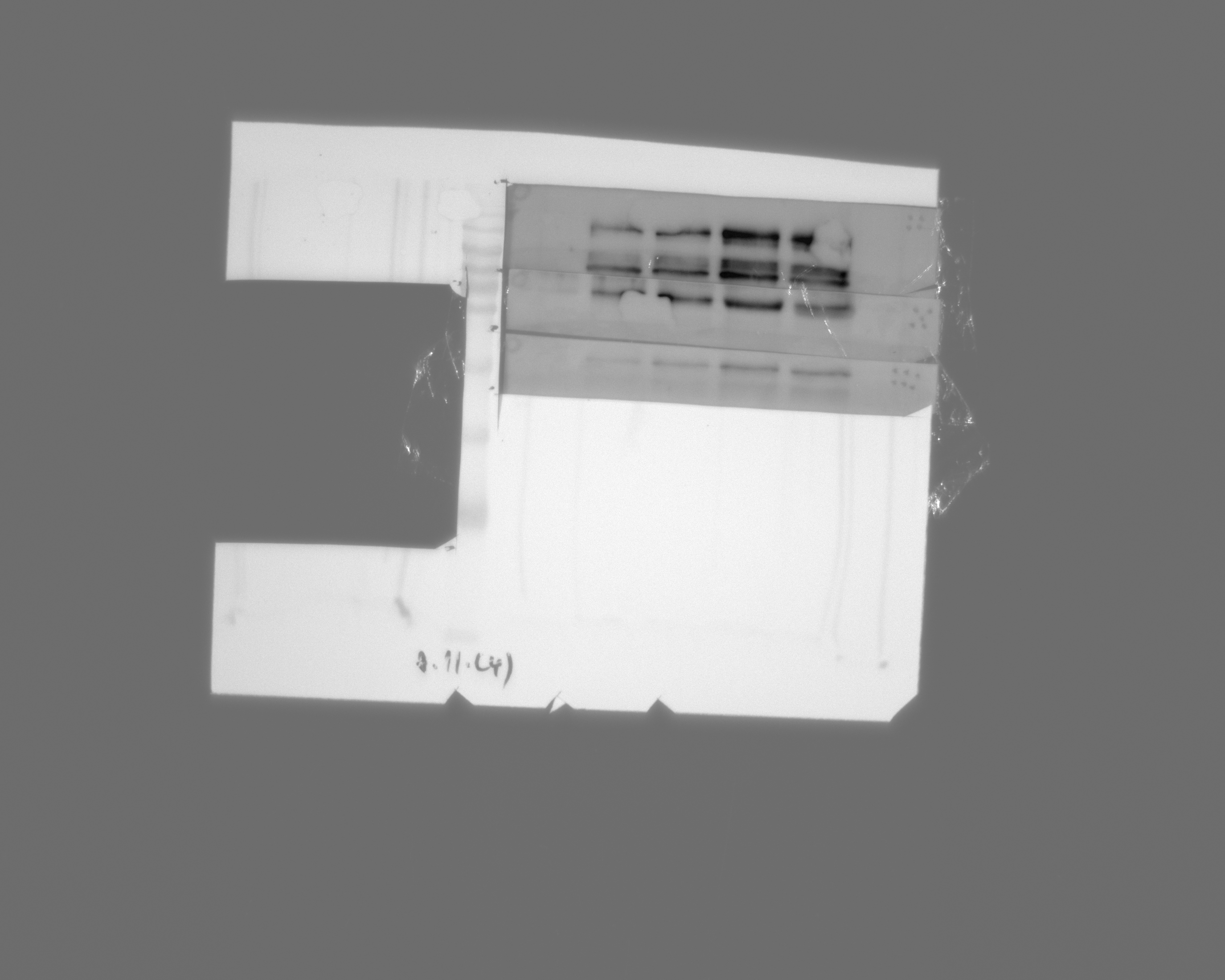


NCL

FBL

wt

Δ25

Δ100

Δ117

free

GFP

free

GFP

Δ117

Δ100

Δ25

wt

NPM IP:
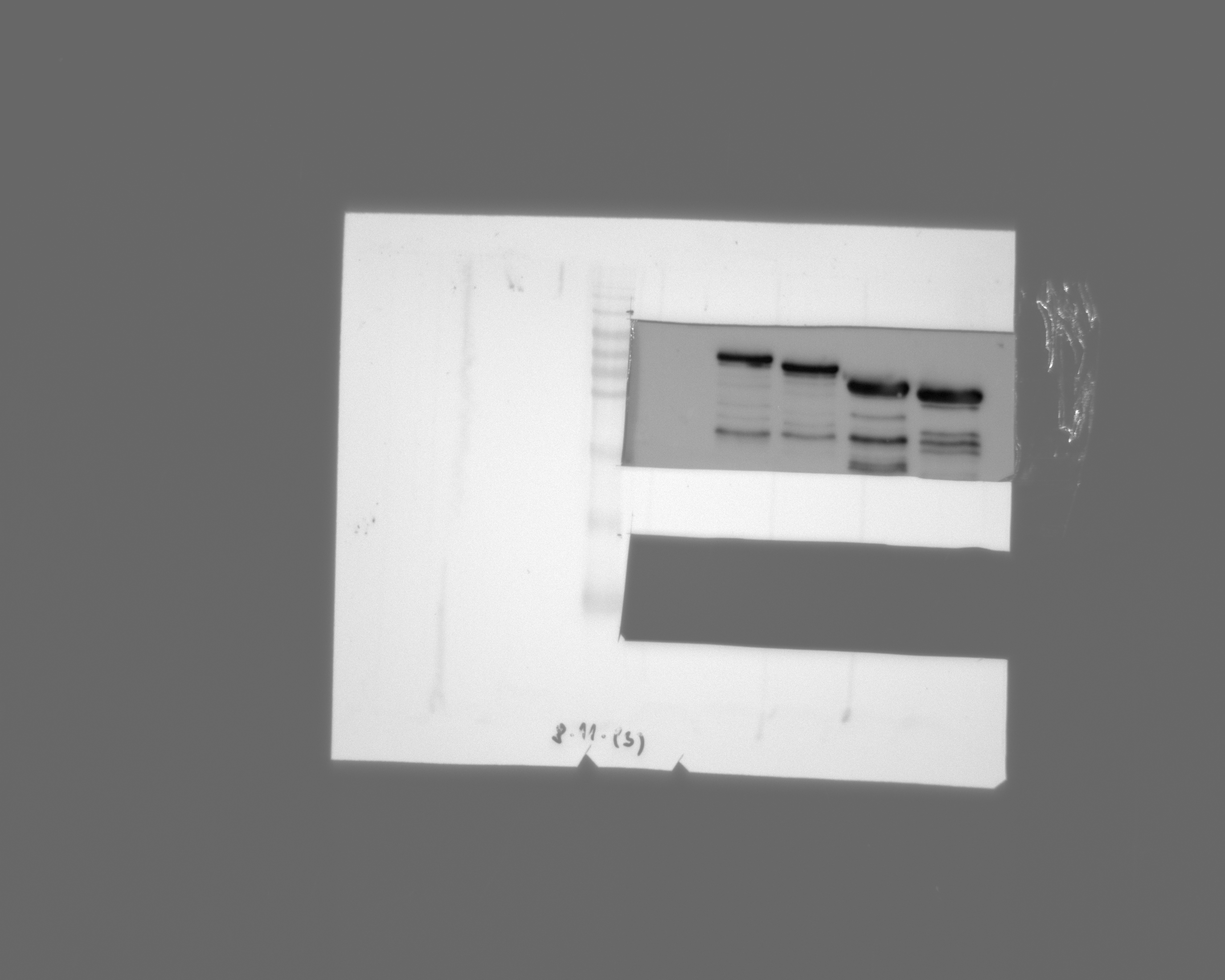
 p14Arf IP:
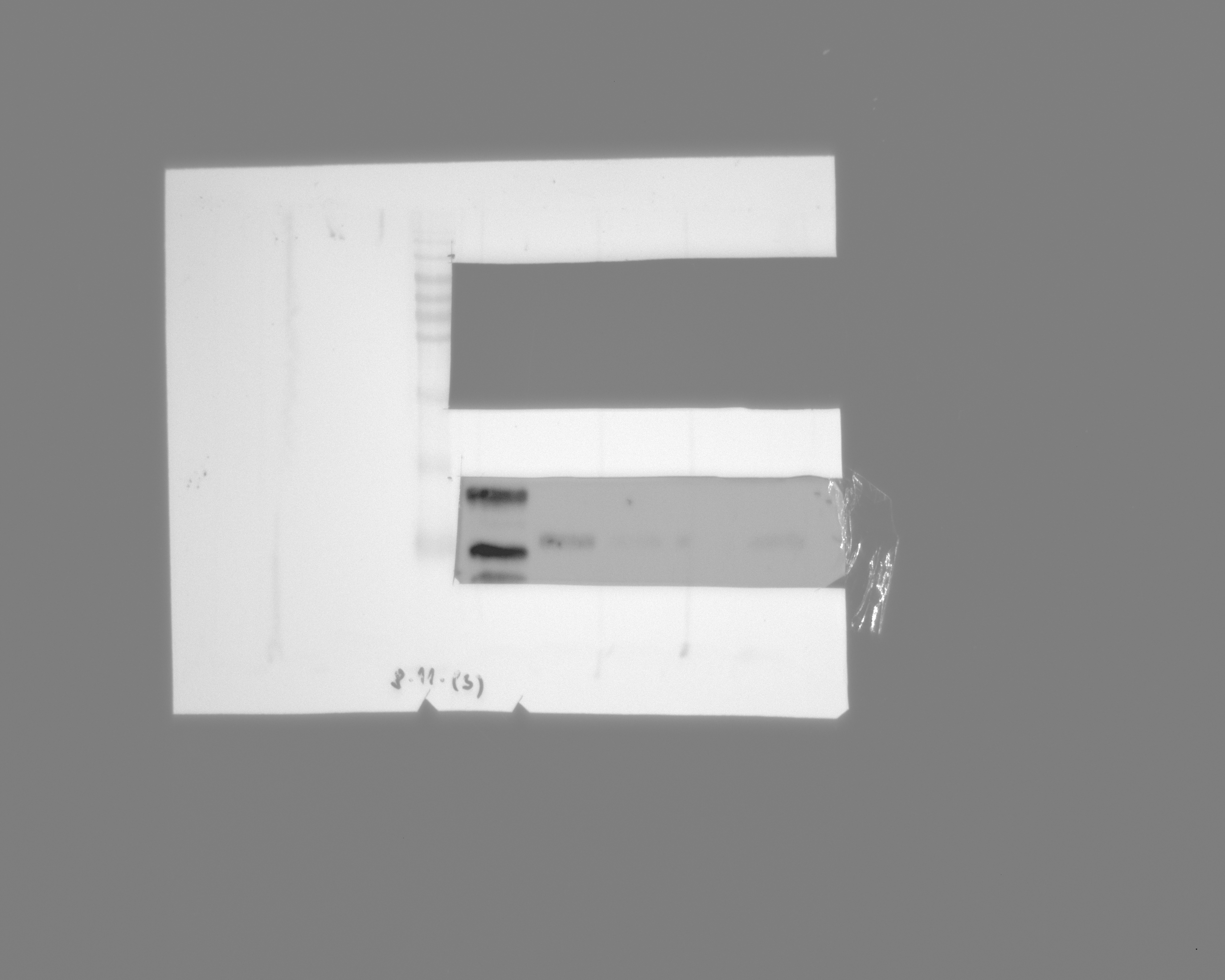


p14Arf

NPM exo

NPM endo

wt

Δ25

Δ100

Δ117

free

GFP

free

GFP

Δ117

Δ100

Δ25

wt

p14Arf IP (enhanced):
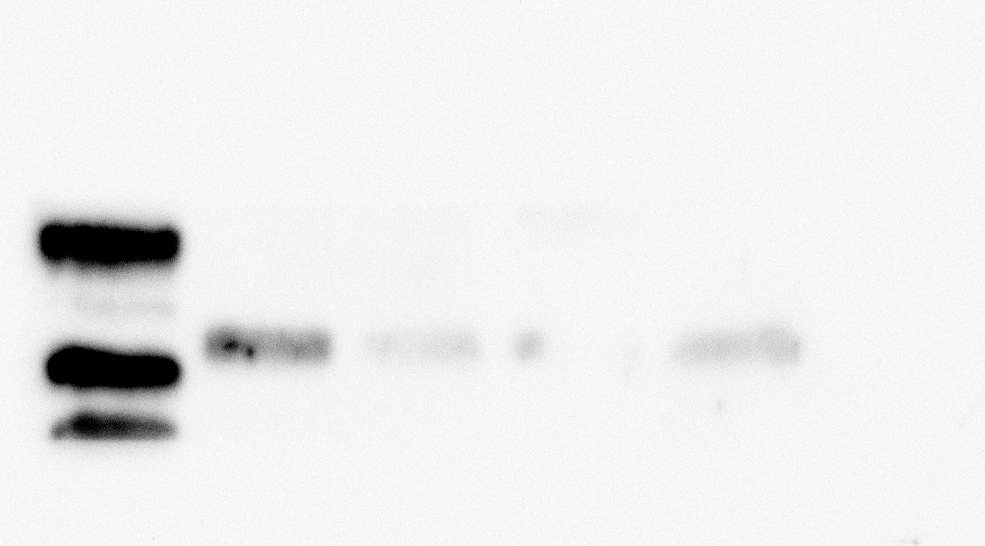


(d):

Input: RFP
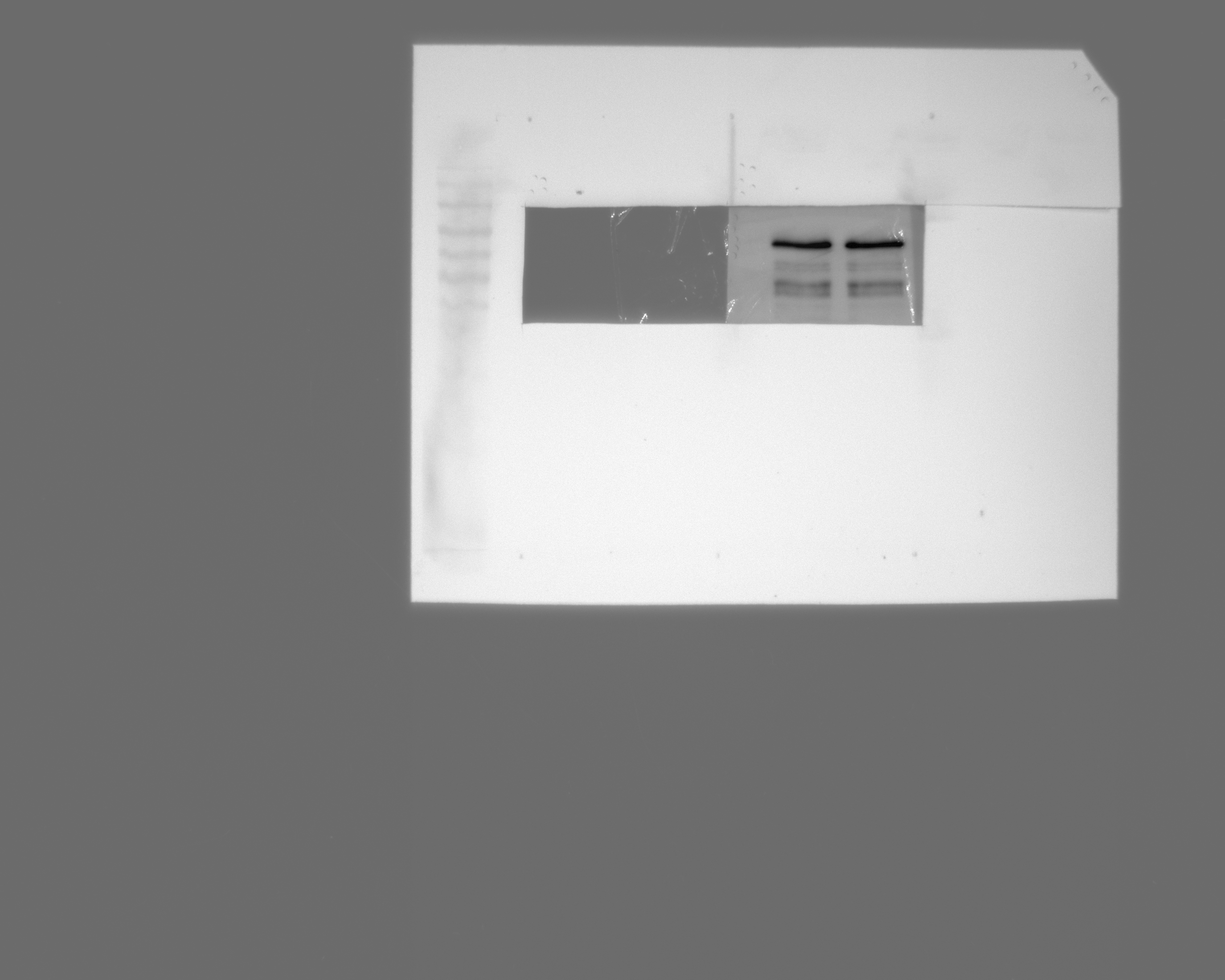
 GFP
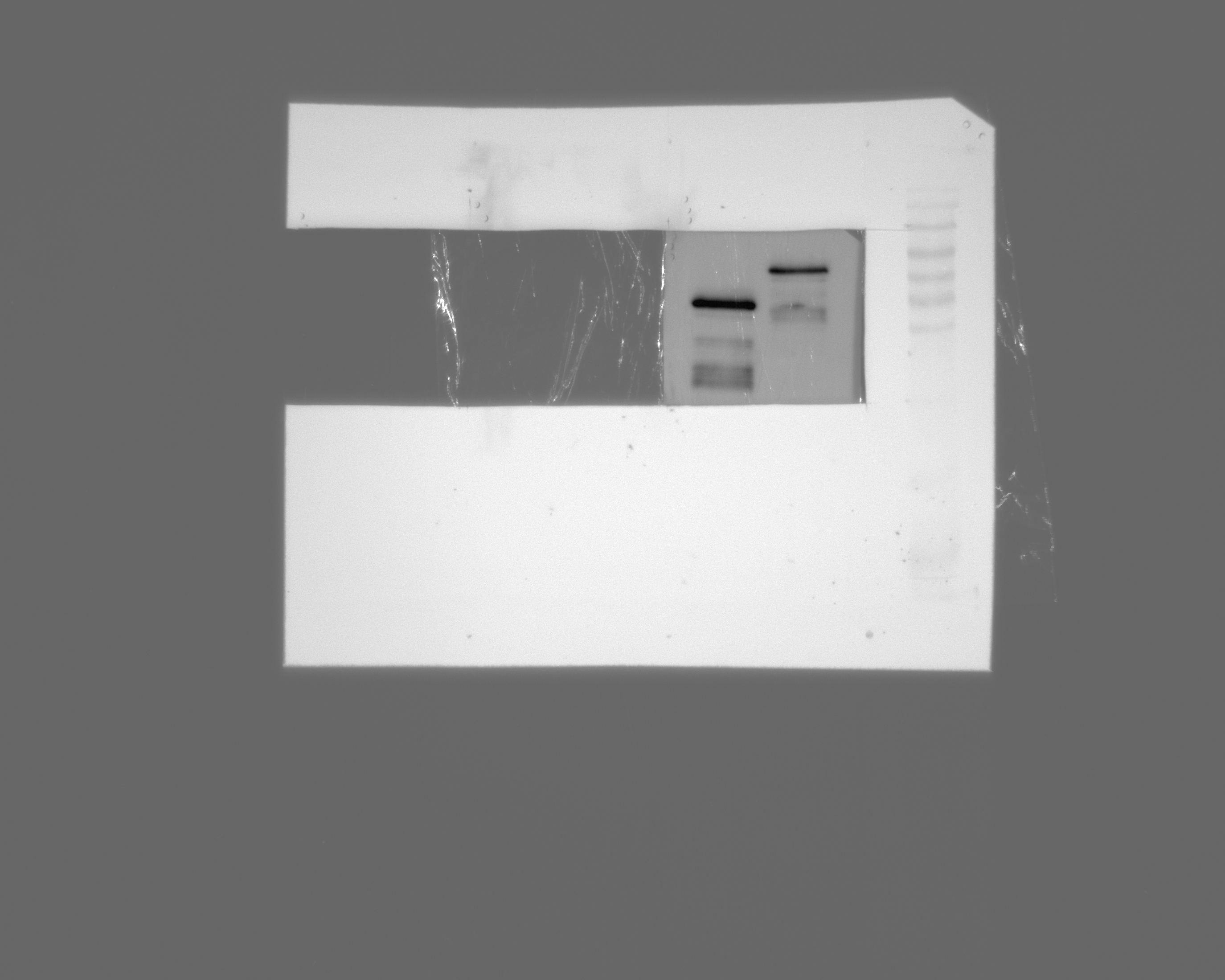
 NPM
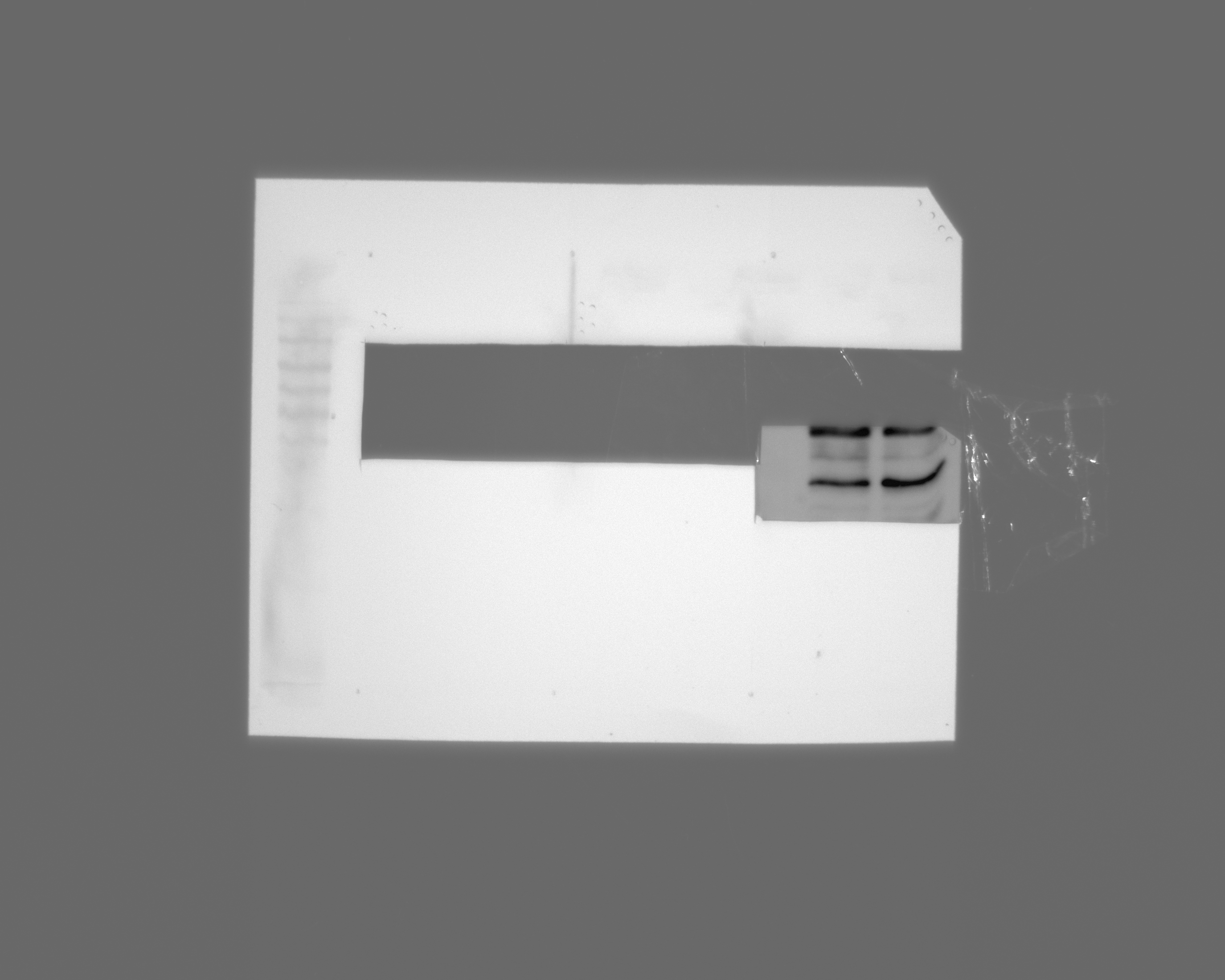


NPM endo

RFP

GFP

C21F

wt

Δ117

wt

C21F

wt

Δ117

wt

Δ117

wt

C21F

wt

IP_GFP: RFP
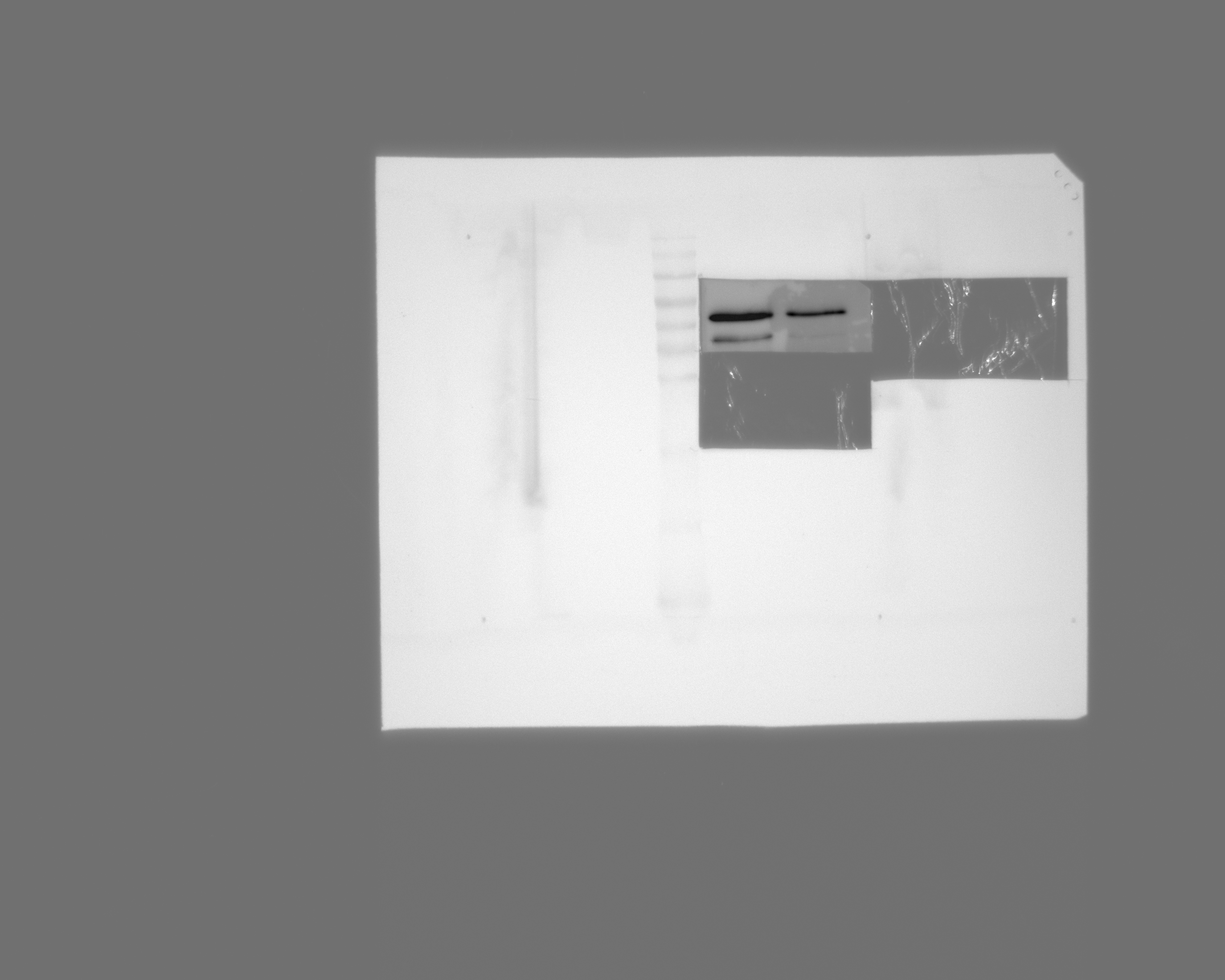
 GFP
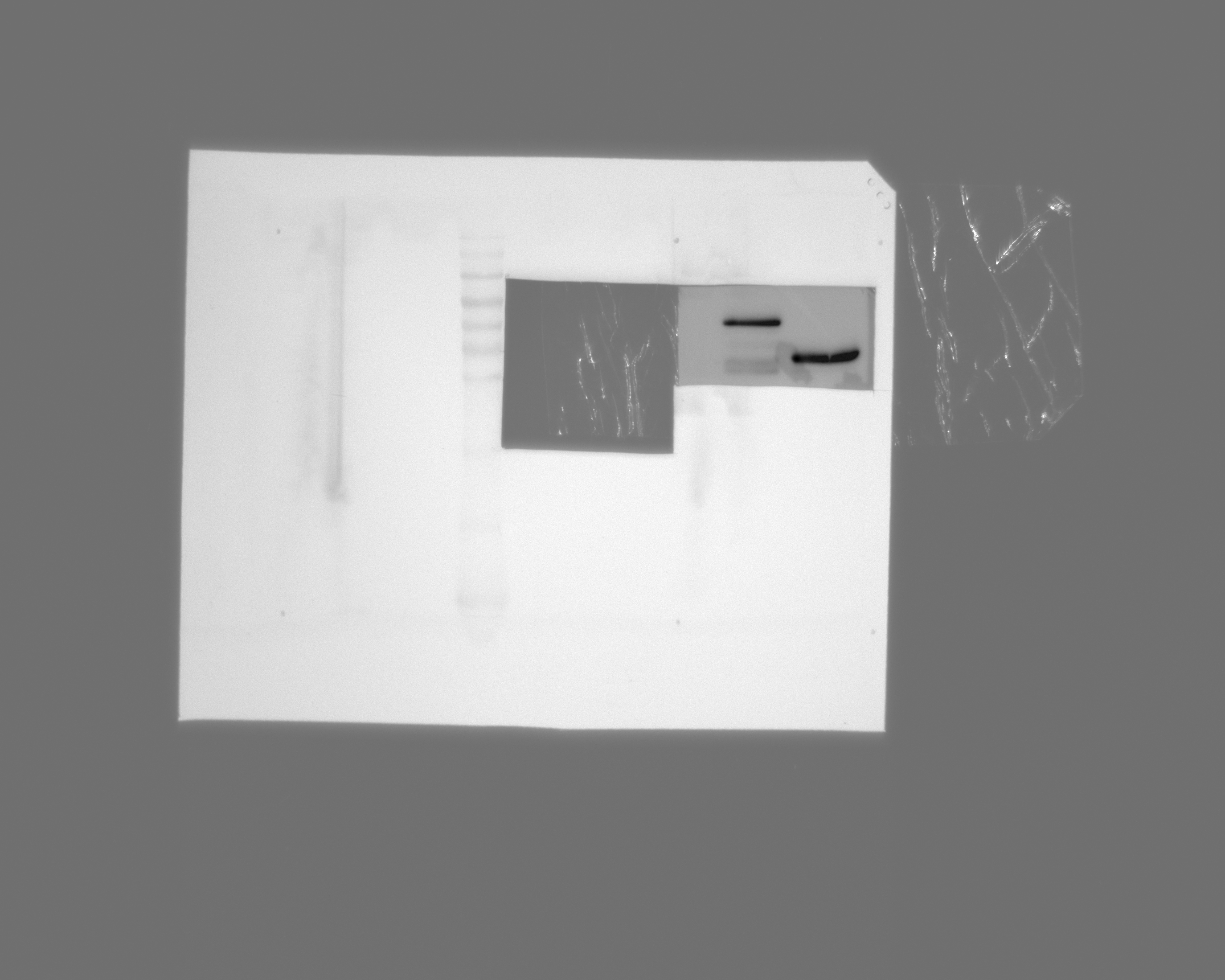
 NPM
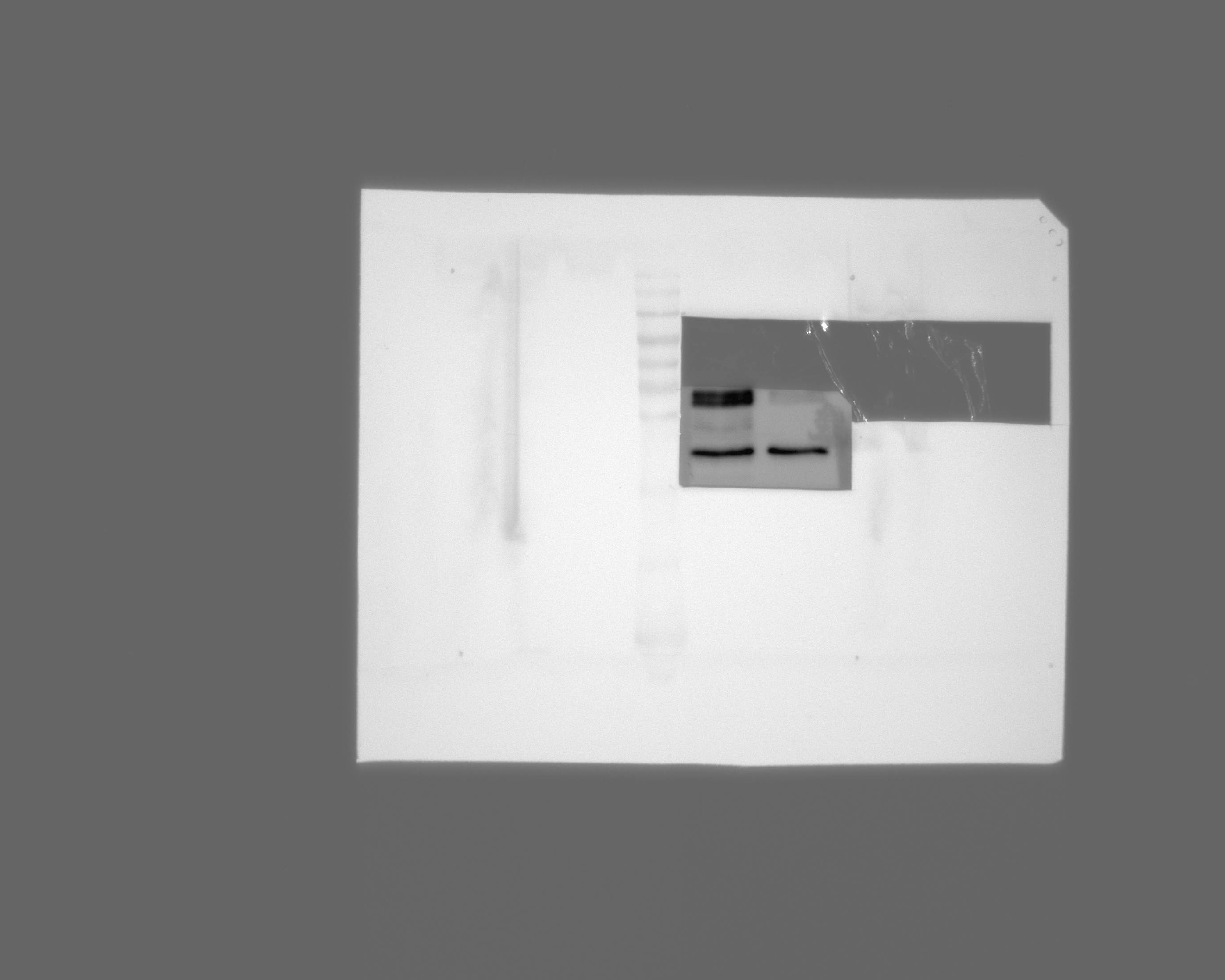


RFP

GFP

NPM endo

C21F

wt

Δ117

wt

C21F

wt

Δ117

wt

C21F

wt

Δ117

wt

IP_RFP: RFP
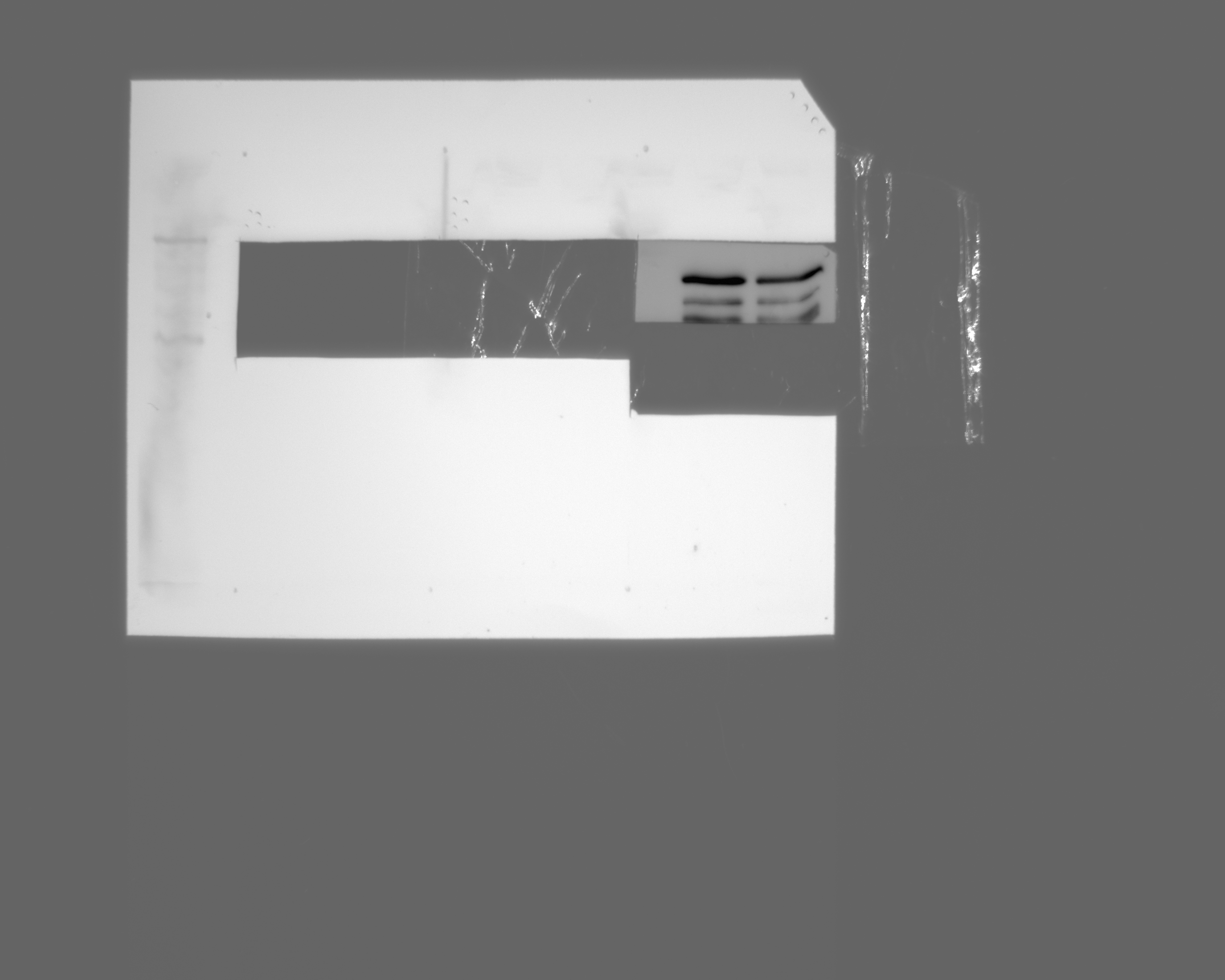
GFP
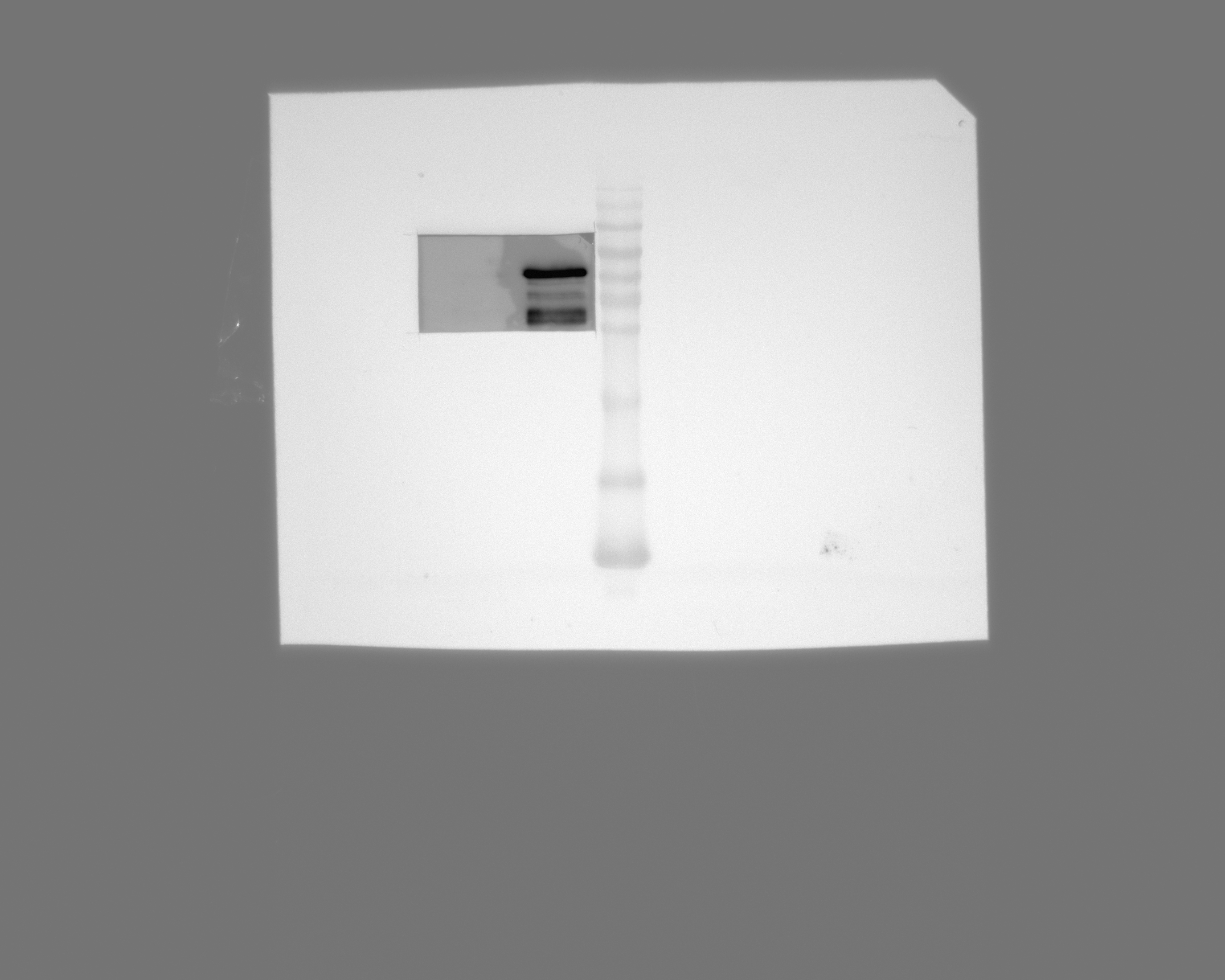
 NPM
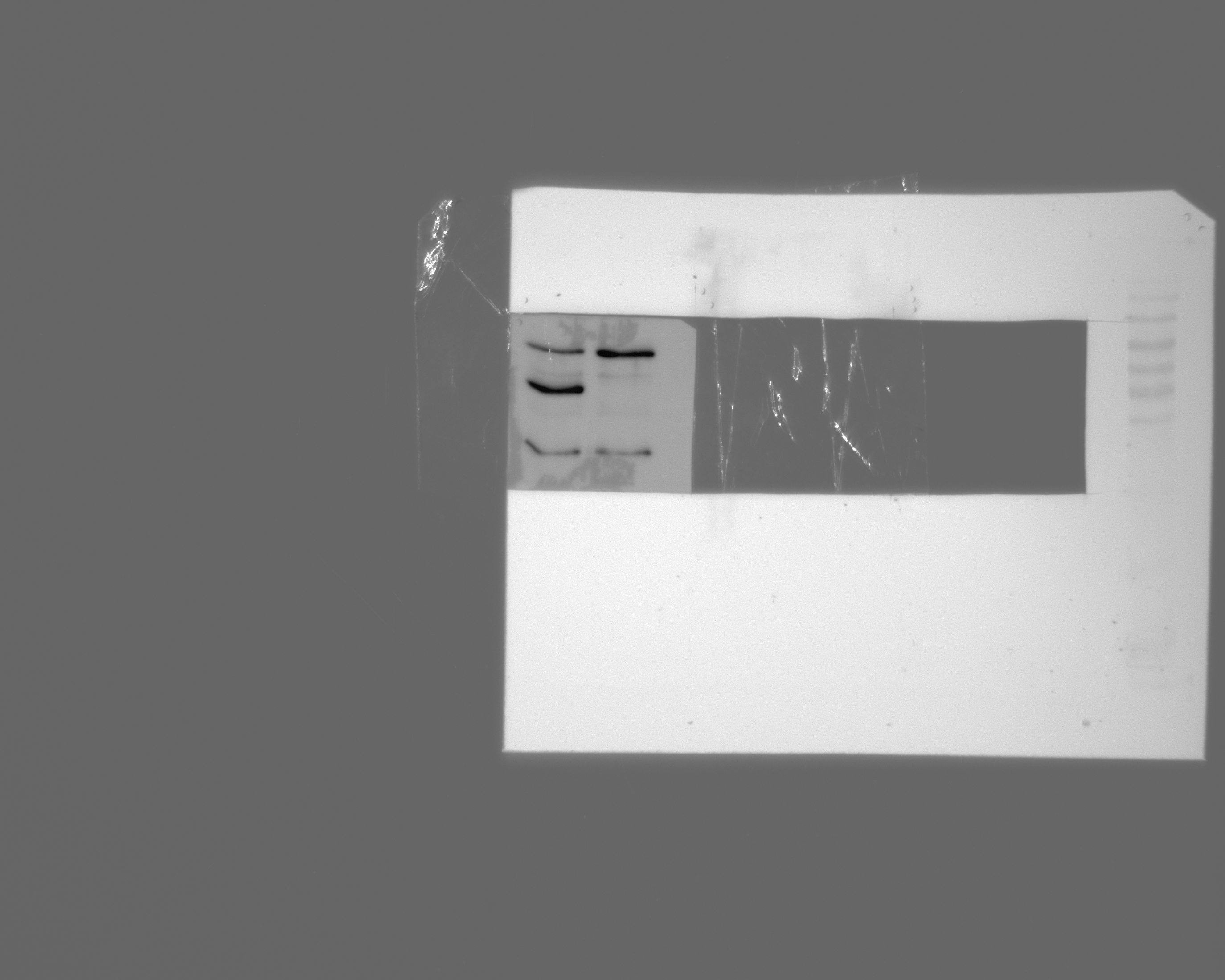


NPM endo

RFP

GFP

C21F

wt

Δ117

wt

C21F

wt

Δ117

wt

C21F

wt

Δ117

wt

**Fig. 7:**

(b):

MV4-11 p53:
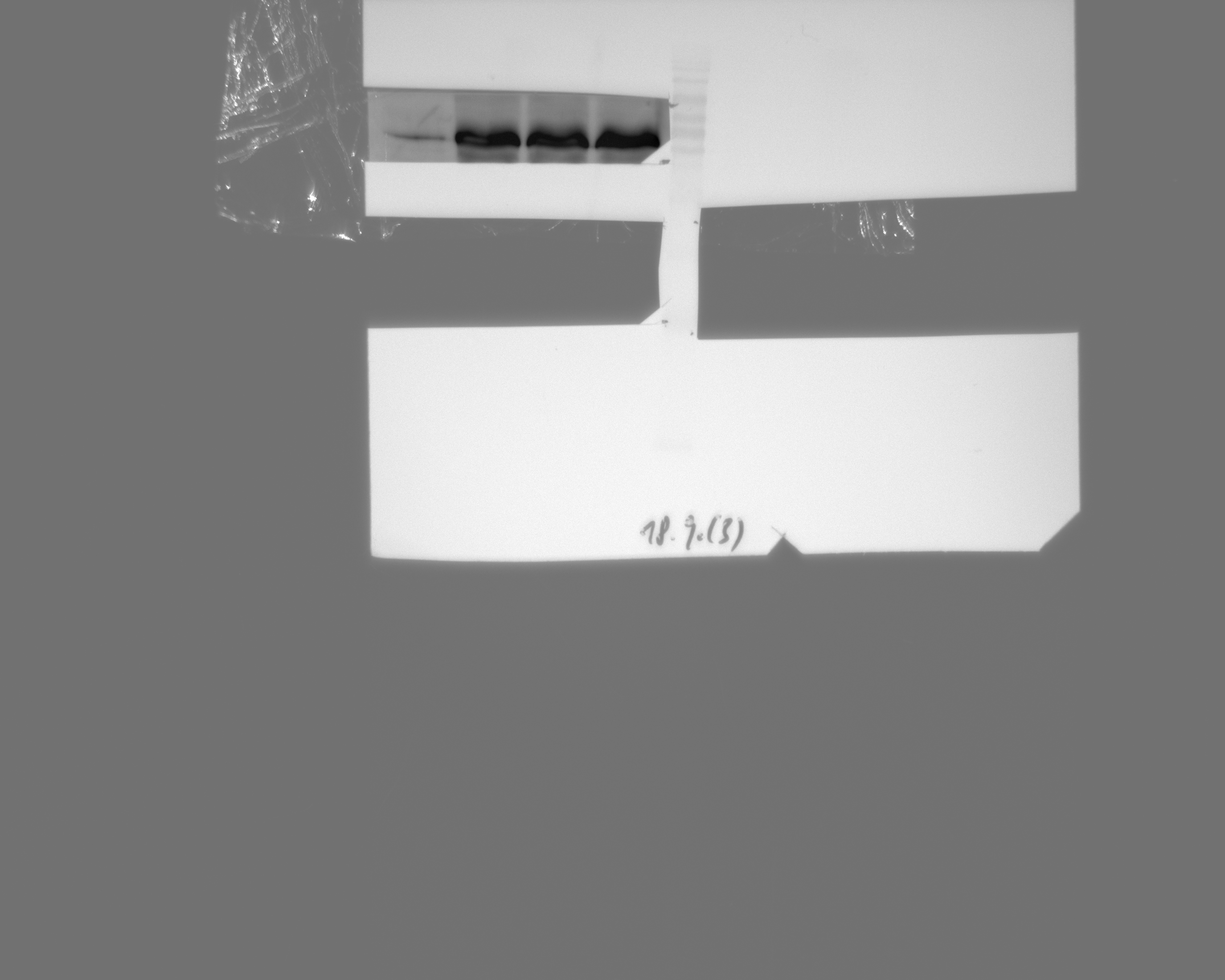
 MV4-11(left)&HL-60(right) β-Actin&casp3
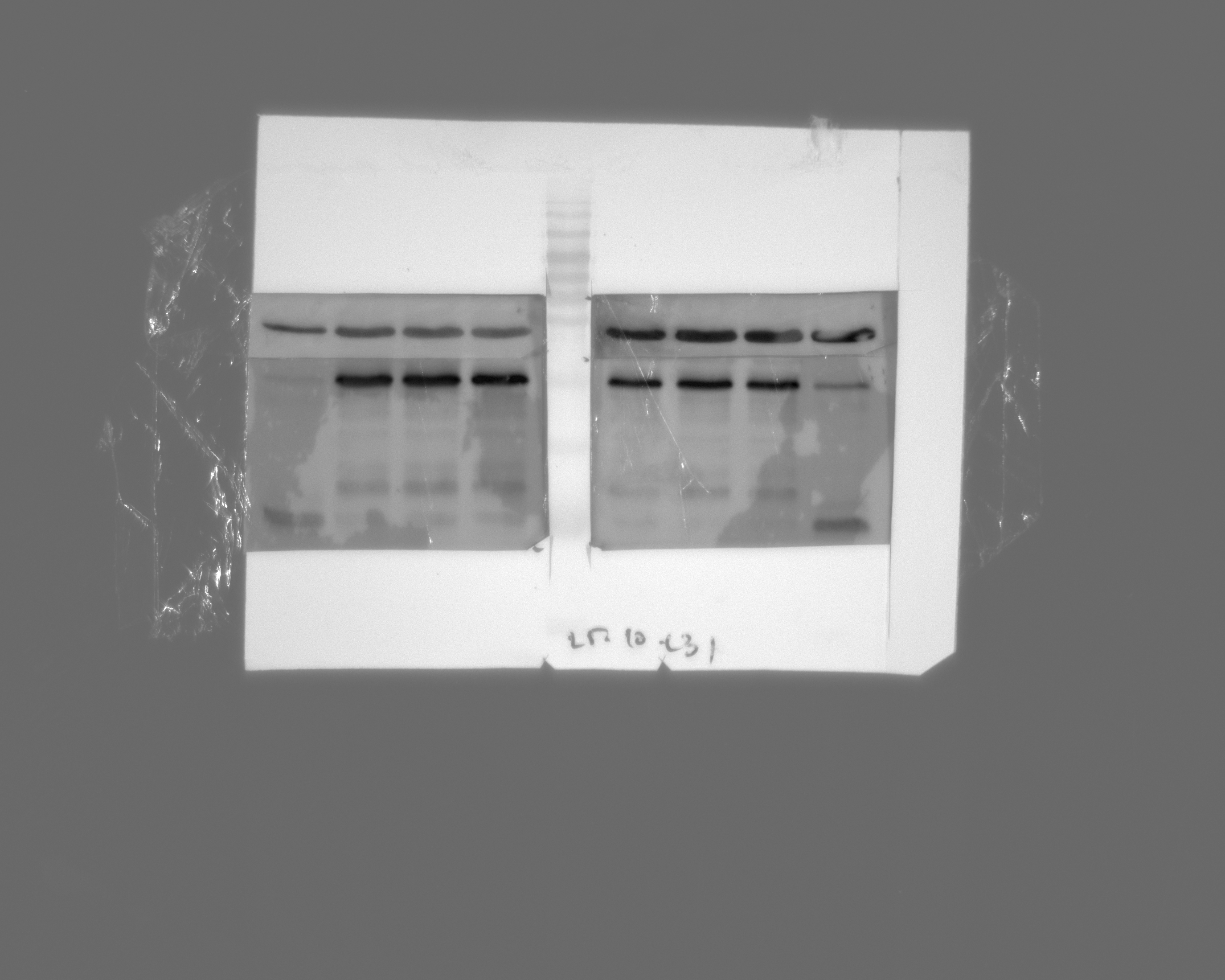


5 2 1 0

p53

0 1 2 5

5 2 1 0

Actin

procaspase3

caspase3

KG-1 casp3 (right):
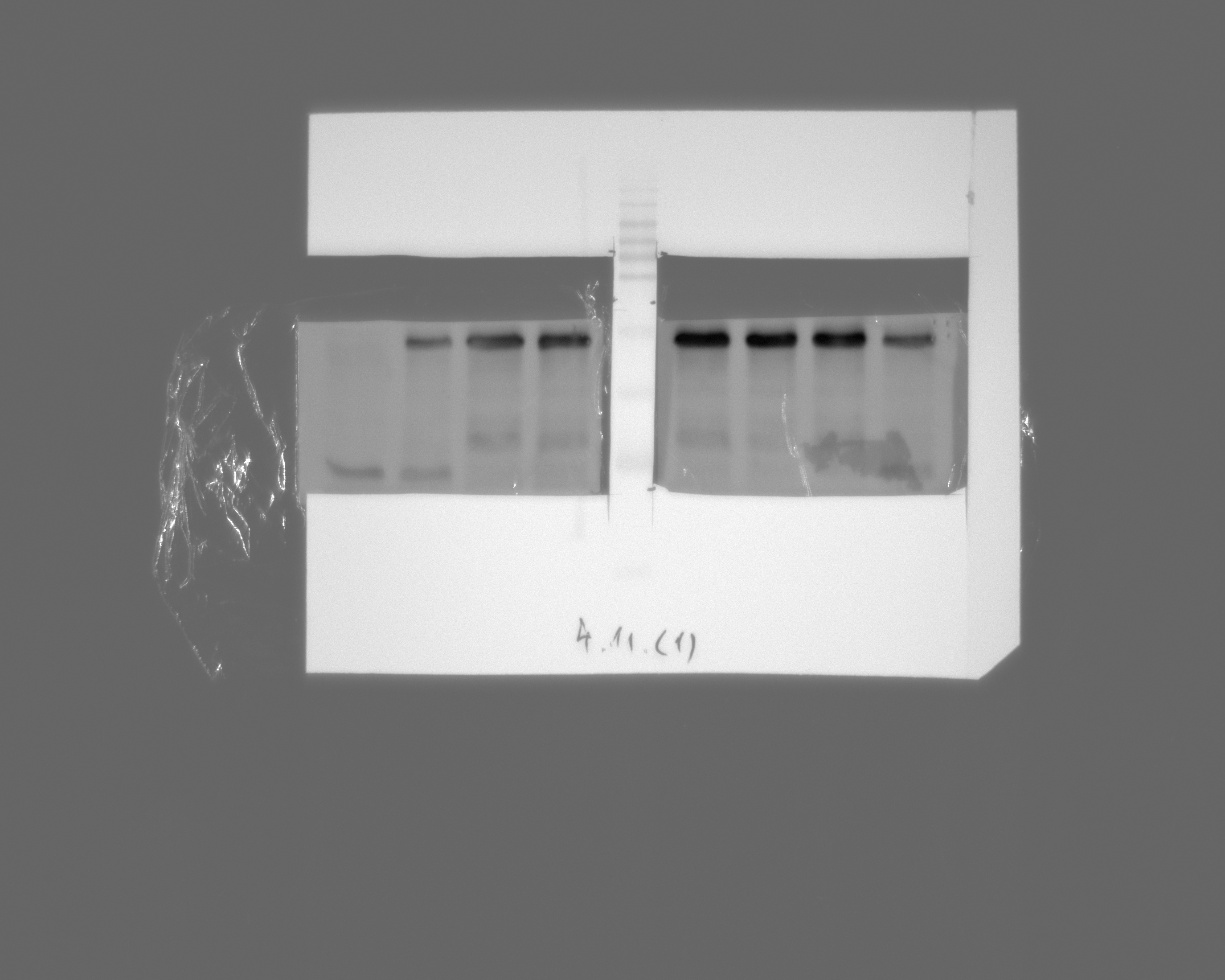
 KG-1 casp3 (enhanced):
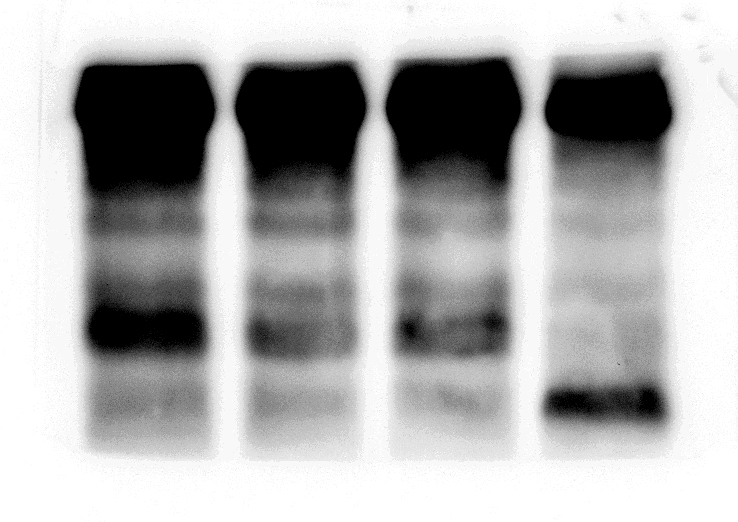


0 1 2 5

caspase3

procaspase3

KG-1 β-Actin (right):
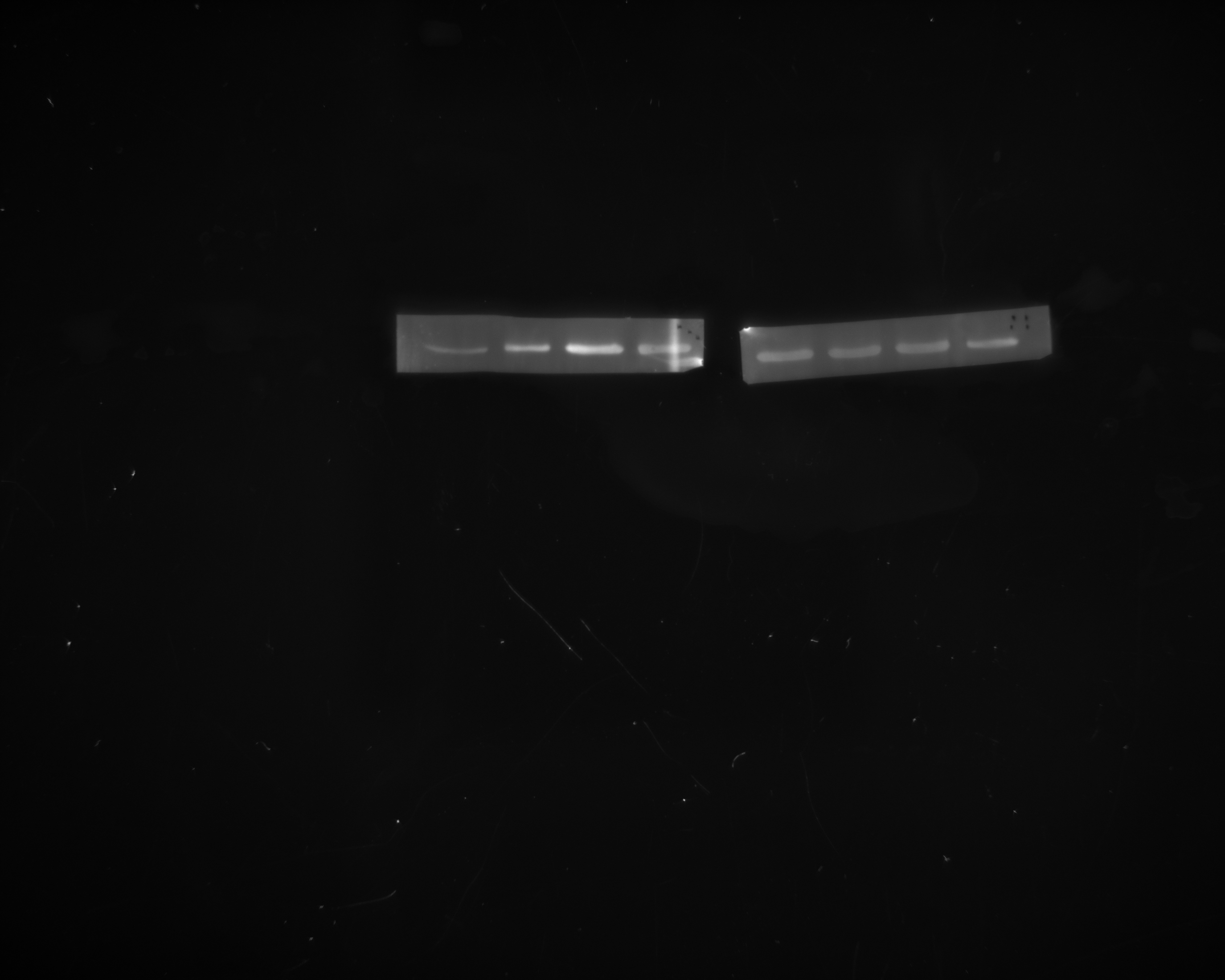
 OCI-AML2 p53:
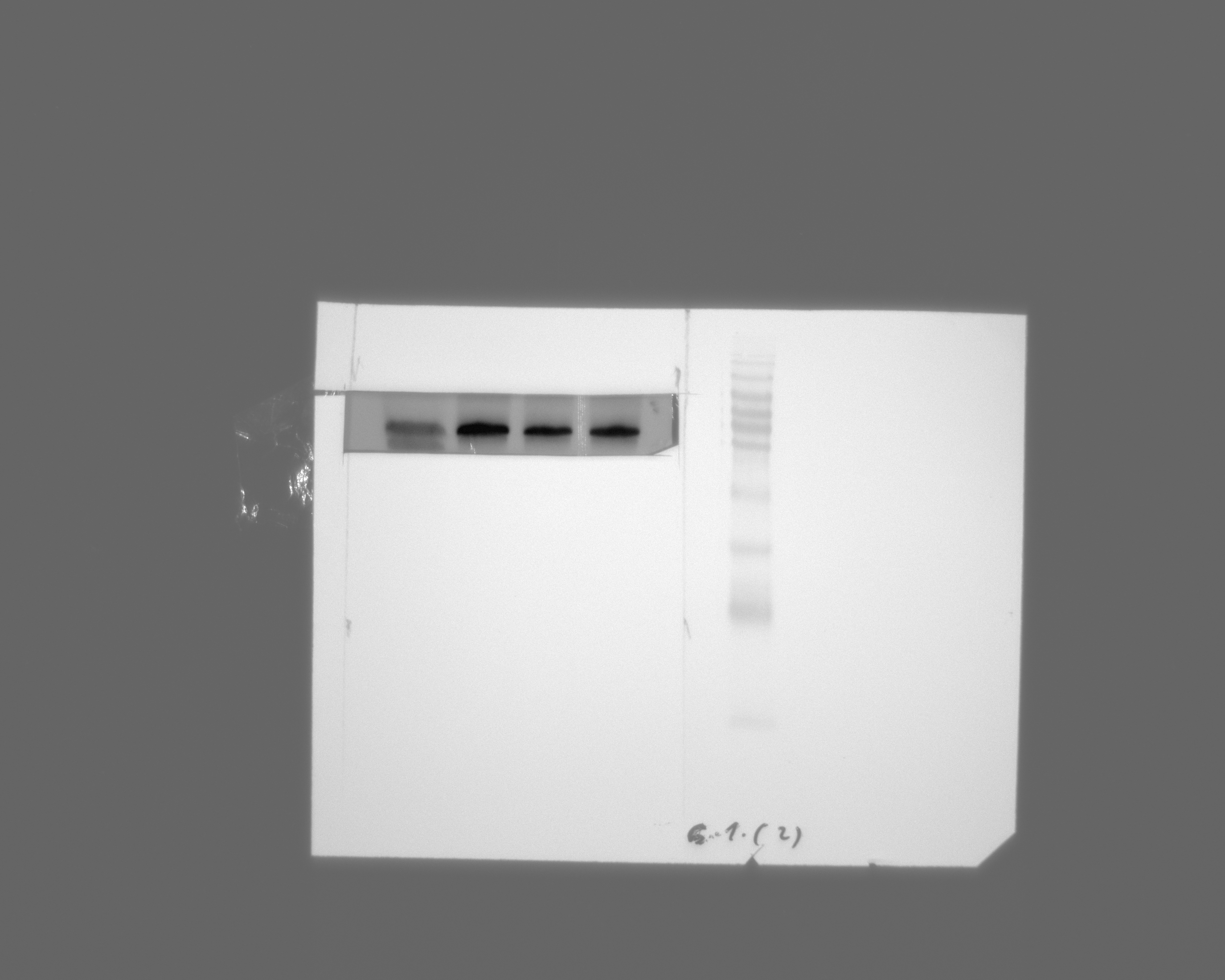


10 5 2 0

0 1 2 5

Actin

p53

OCI-AML2 β-Actin:
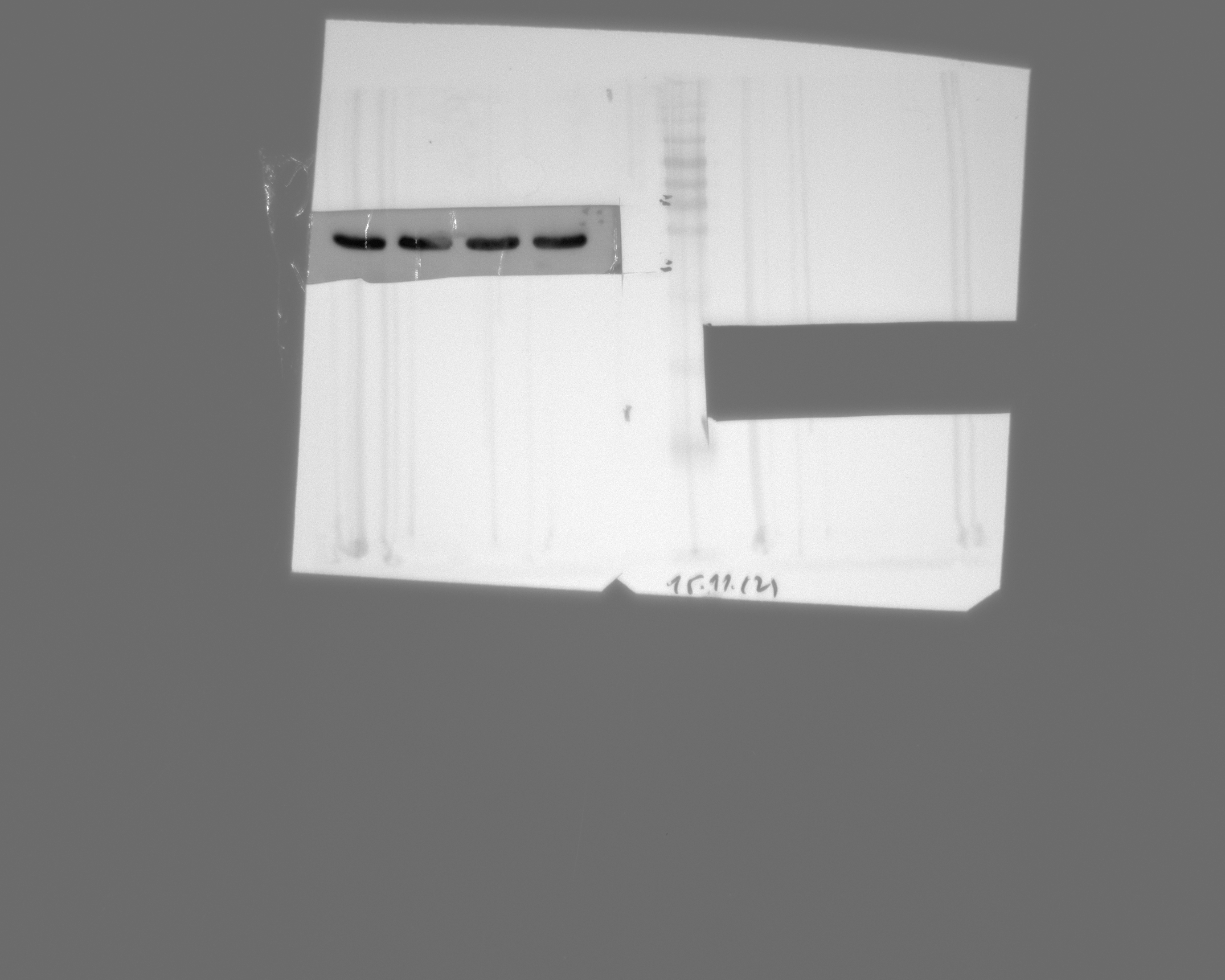
 OCI-AML3 p53:
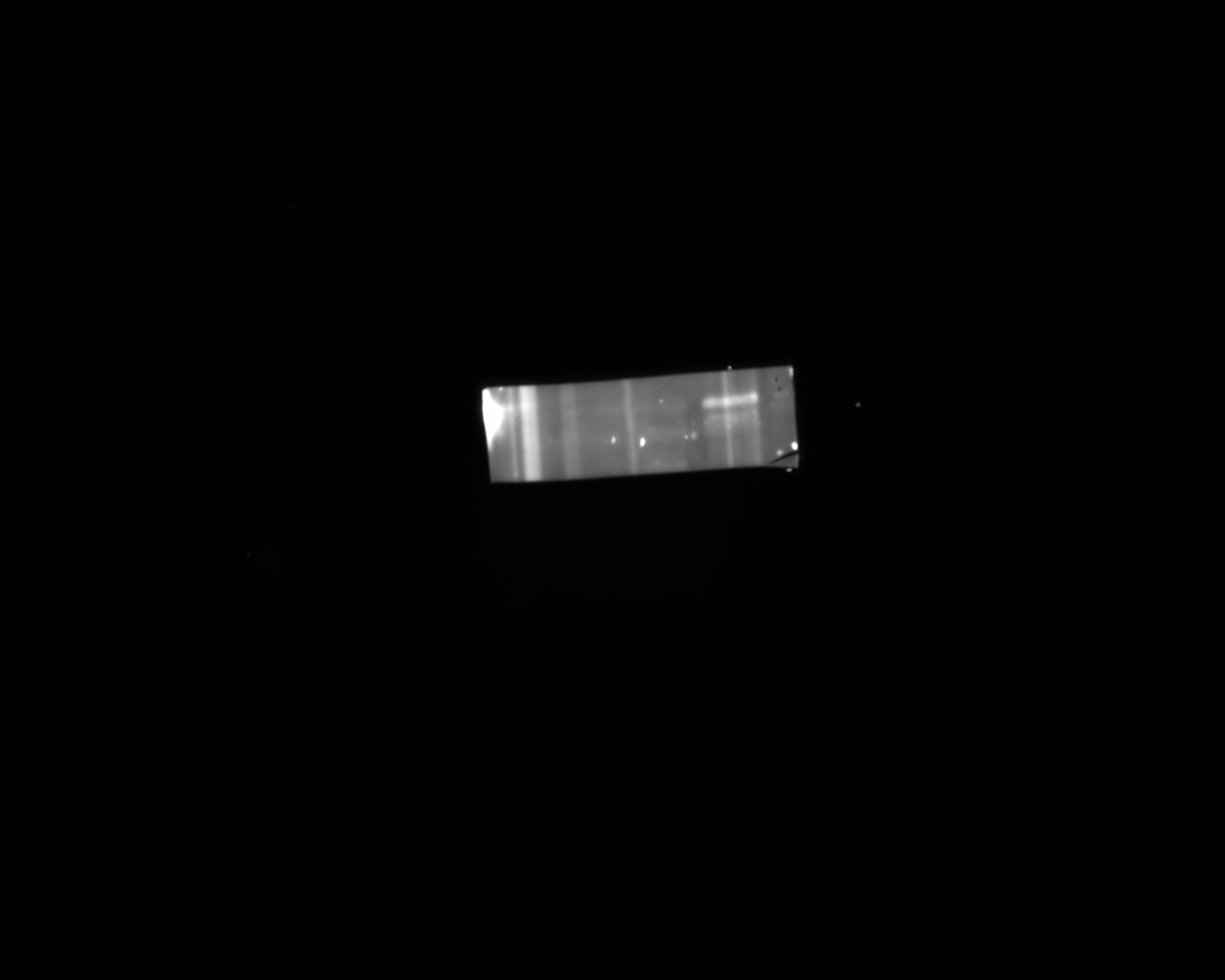


Actin

0 2 5 10

10 5 2 0

p53

OCI-AML2 (left) and 3 (right) casp3:
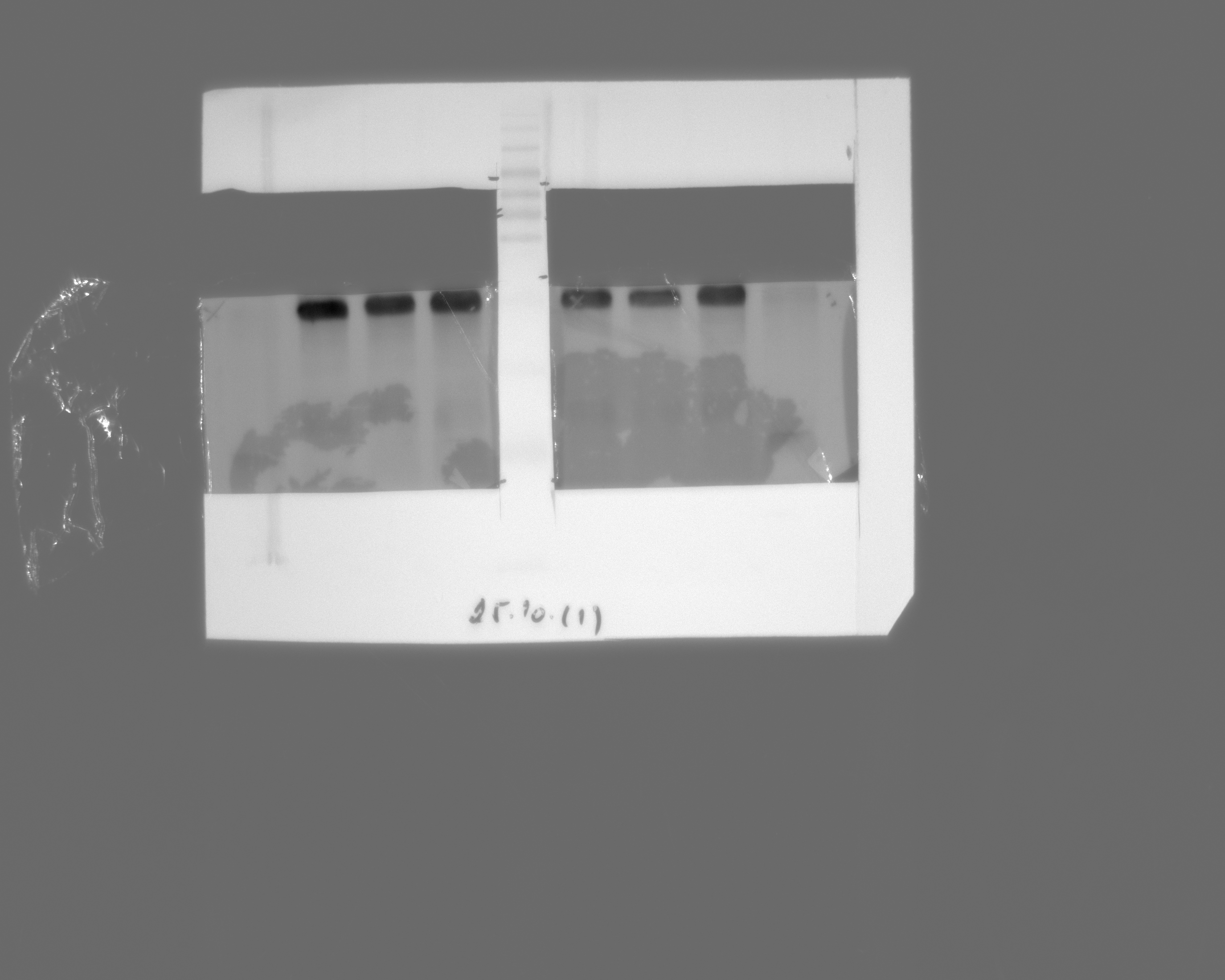
, enhanced:
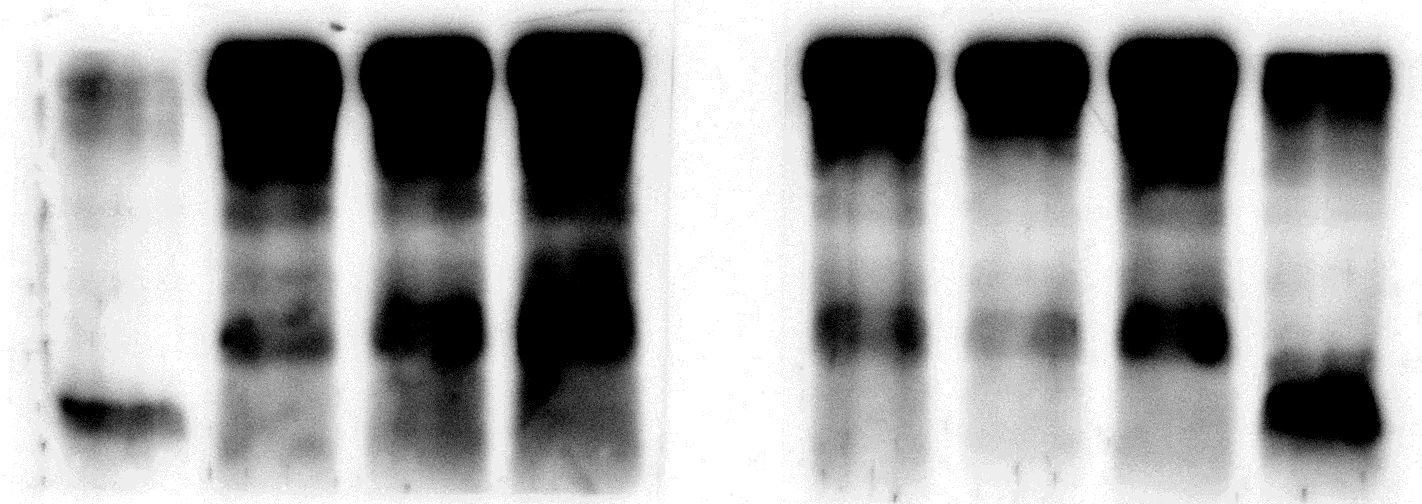
 OCI-AML3 β-Actin:
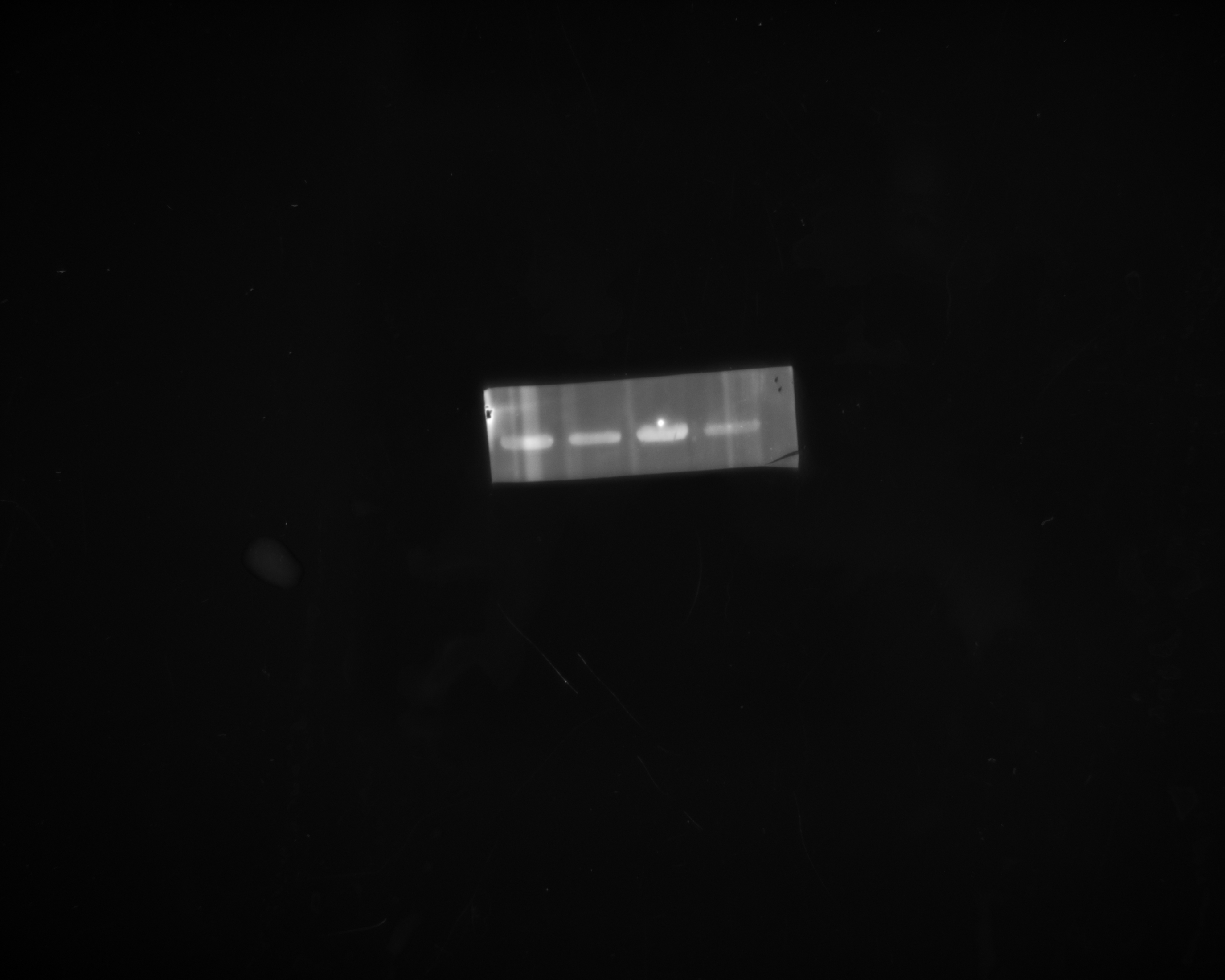
 HeLa β-Actin:
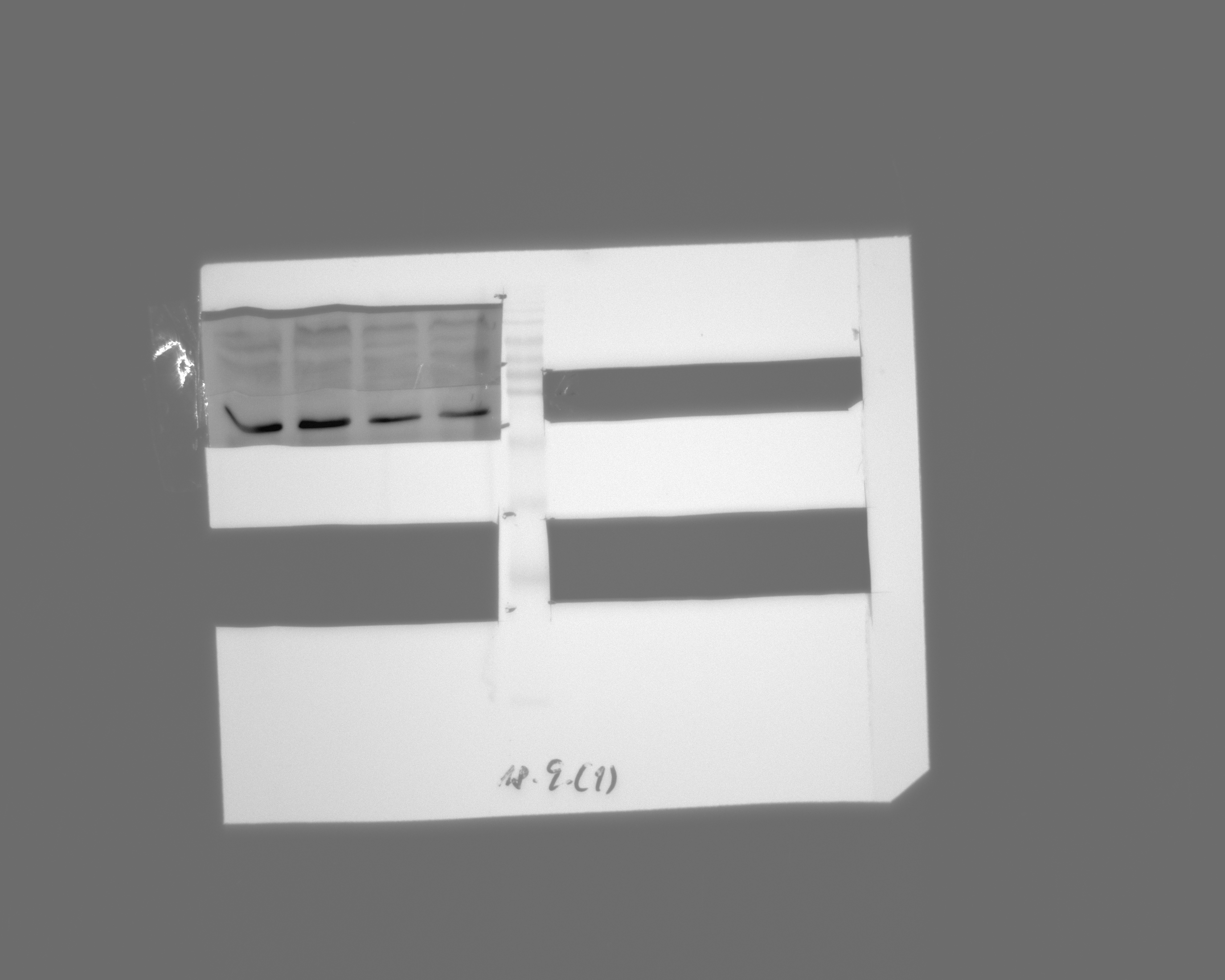


10 5 2 0

0 2 5 10

0 2 5 10

10 5 2 0

Actin

Actin

caspase3

procaspase3

HeLa p53&casp3 (left):
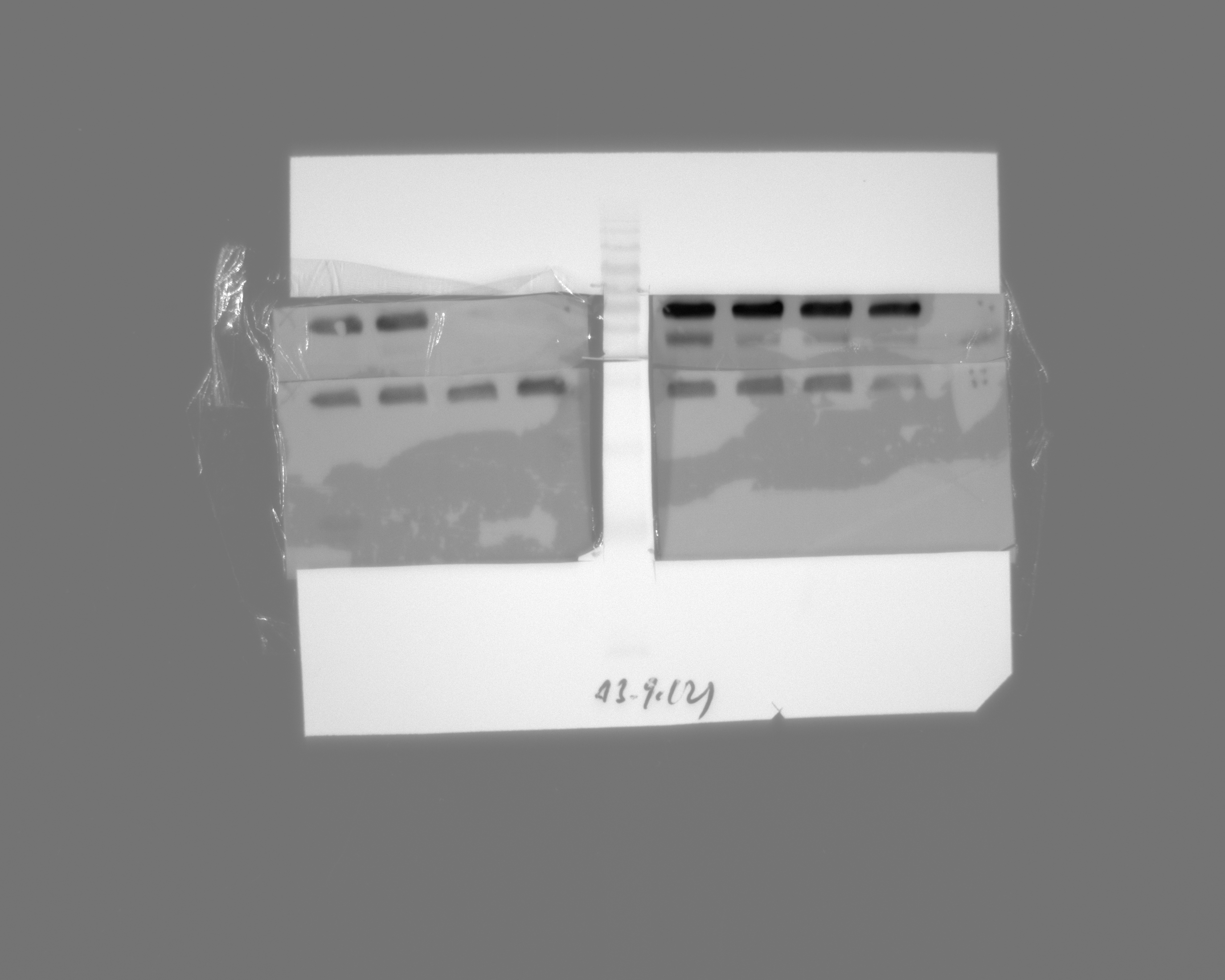
, enhanced:
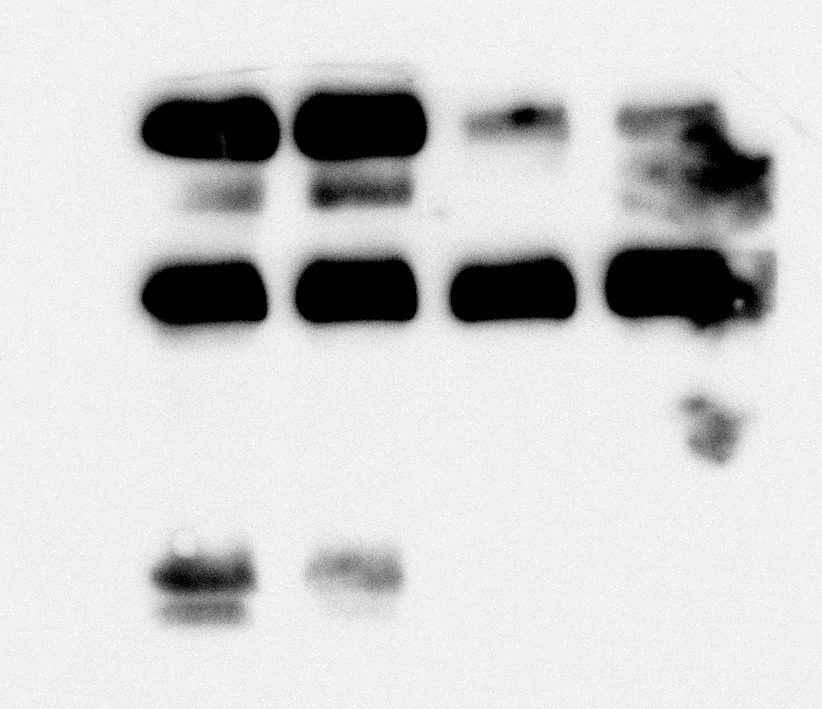


10 5 2 0

procaspase3

caspase3

p53

(c):

#41 (left)
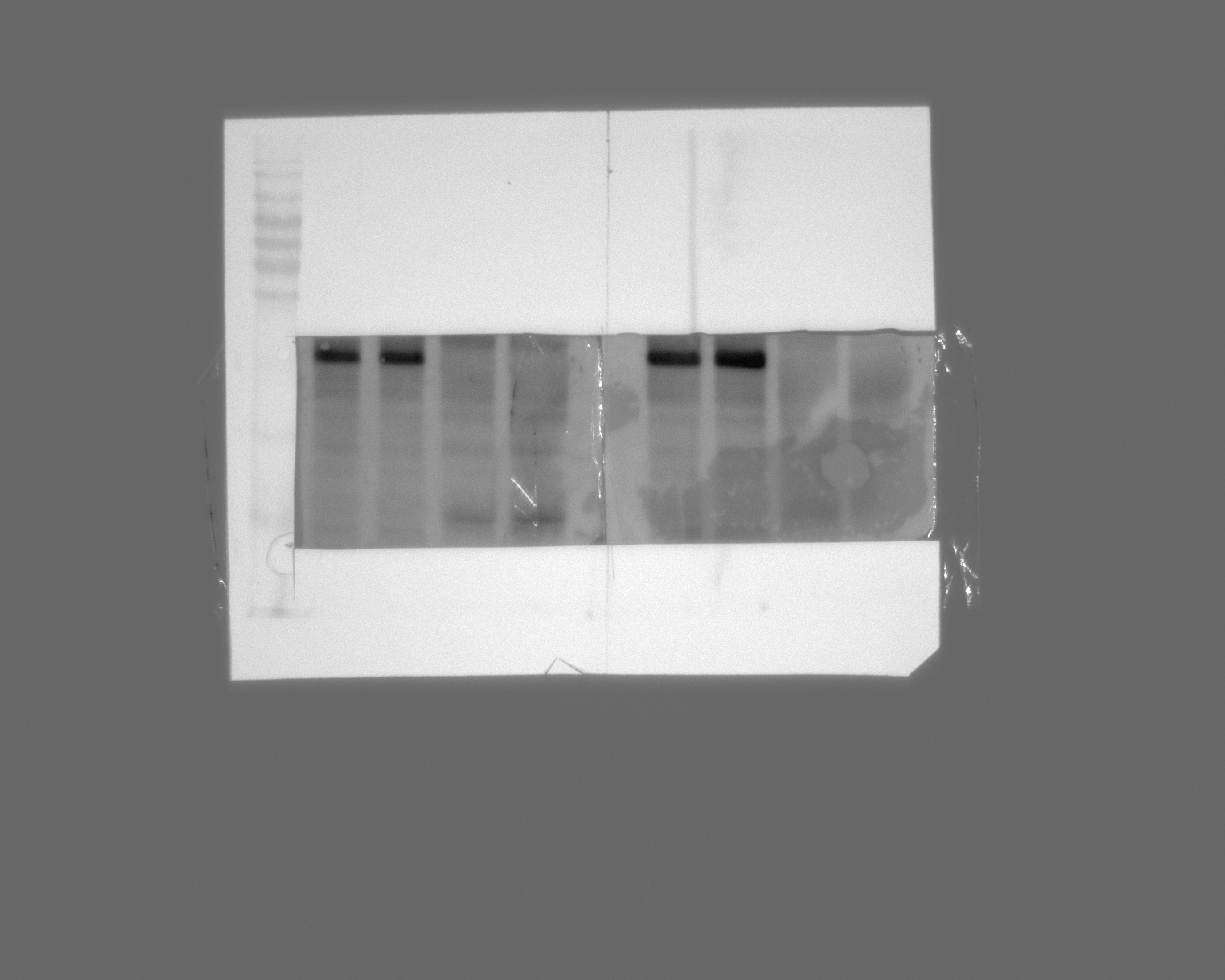
 #42&43
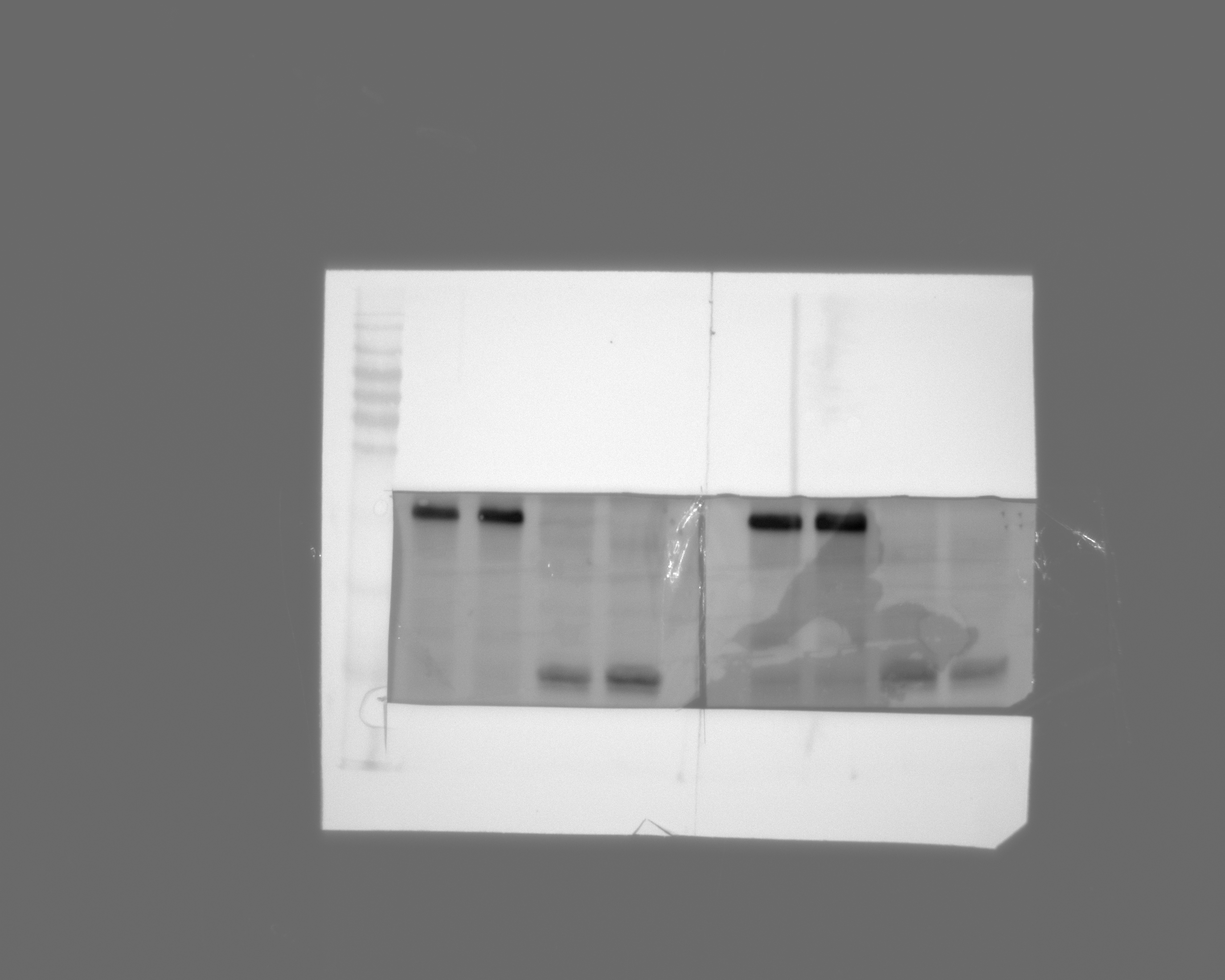
 #45
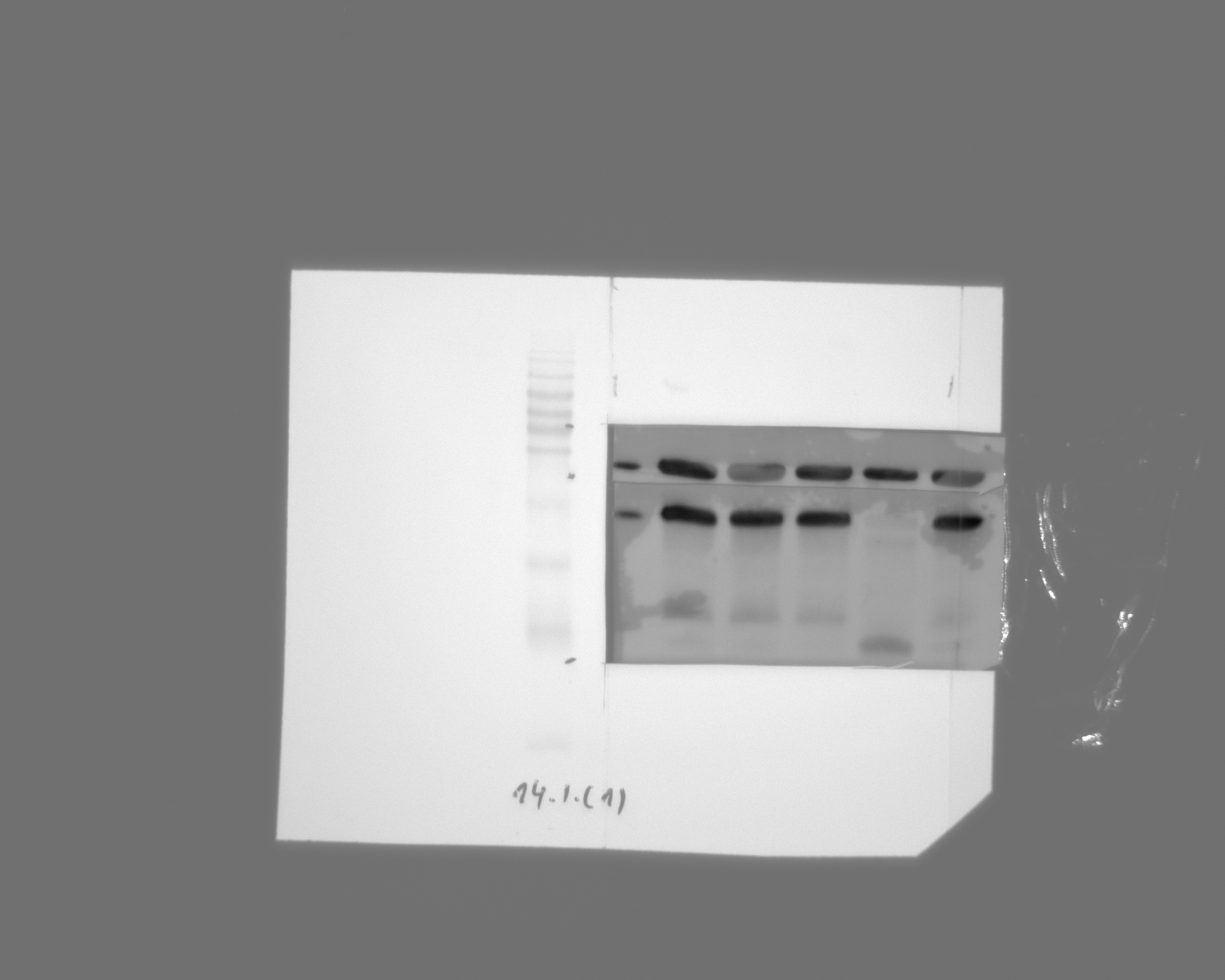


caspase3

procaspase3

procaspase3

caspase3

0 2 5 10

0 2 5 10

0 2 5 10

0 2 5 10

caspase3

procaspase3

**Fig. 11:**

NCL (Input):
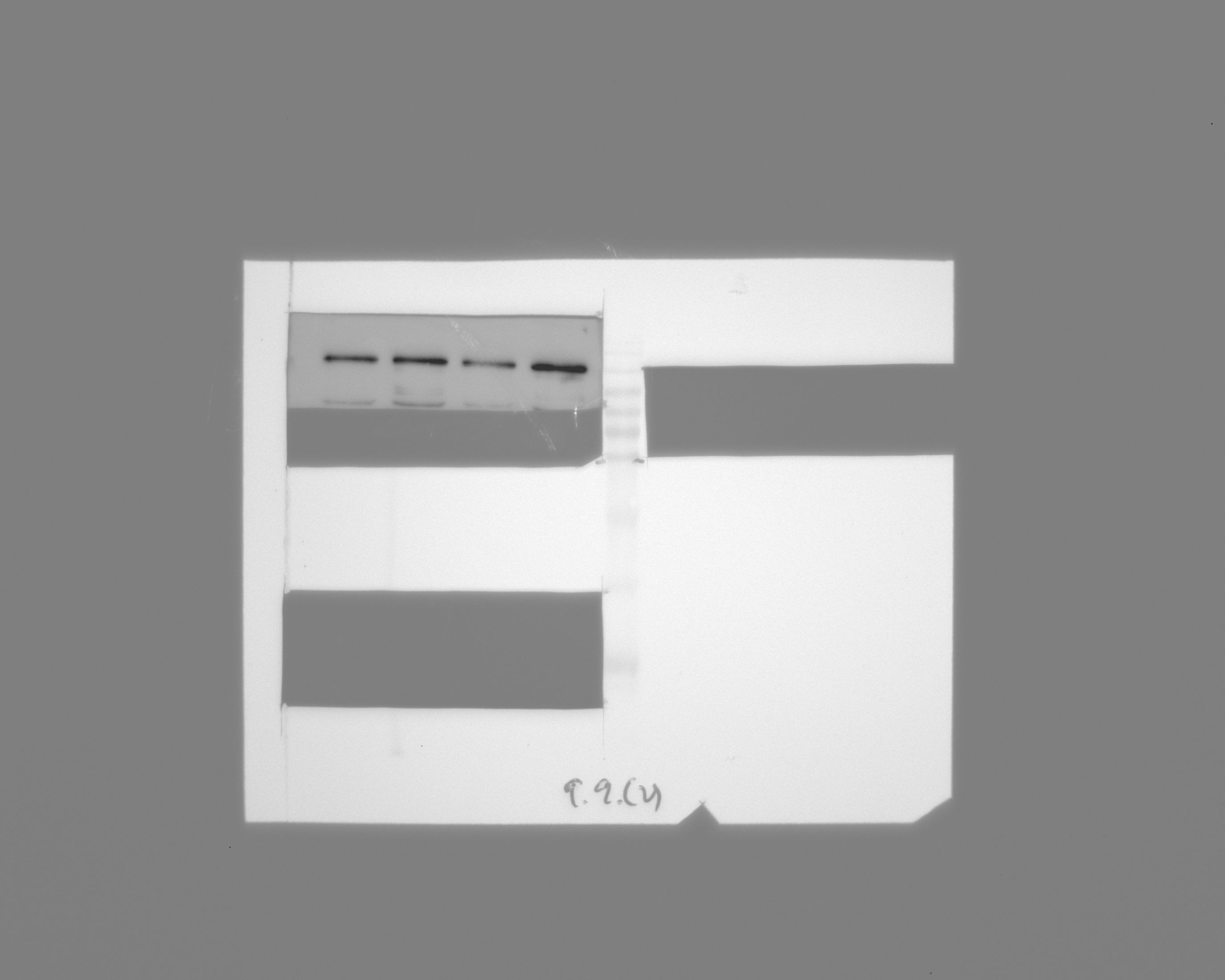
 GFP (Input):
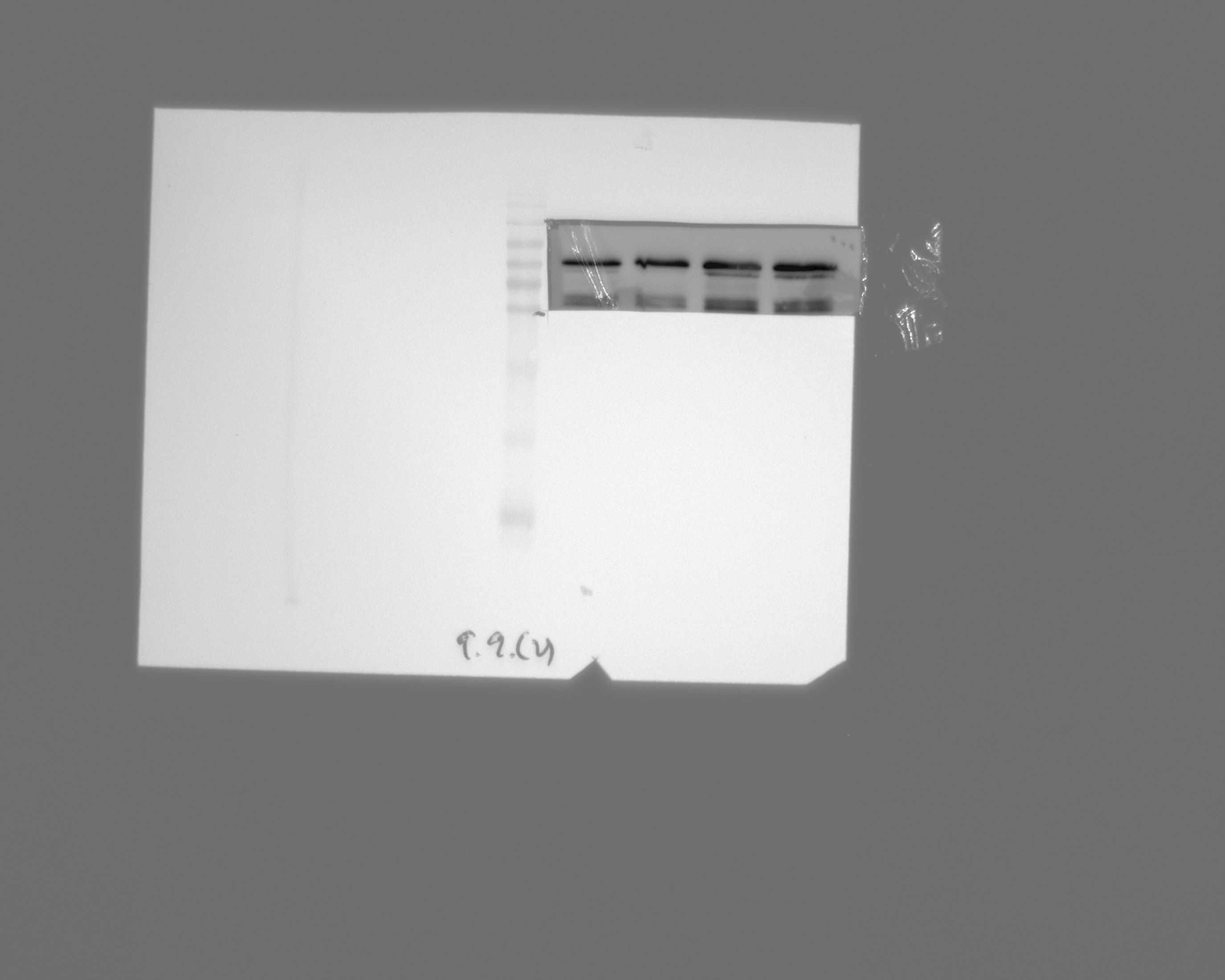


mut

wt

- +

- +

wt

mut

+ -

+ -

GFP

NCL

NPM&RFP (Input):
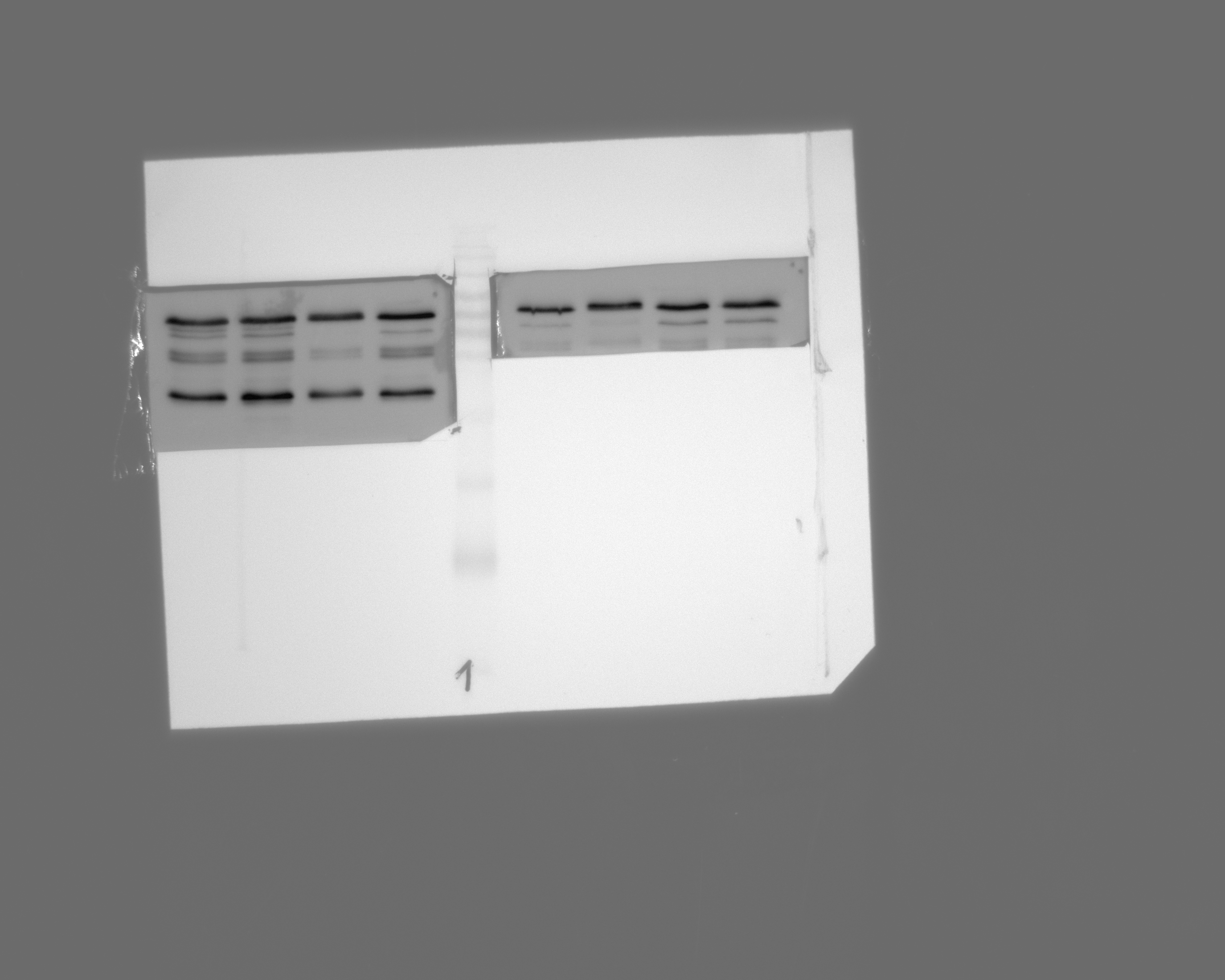
 NCL (IP_GFP&IP_RFP):
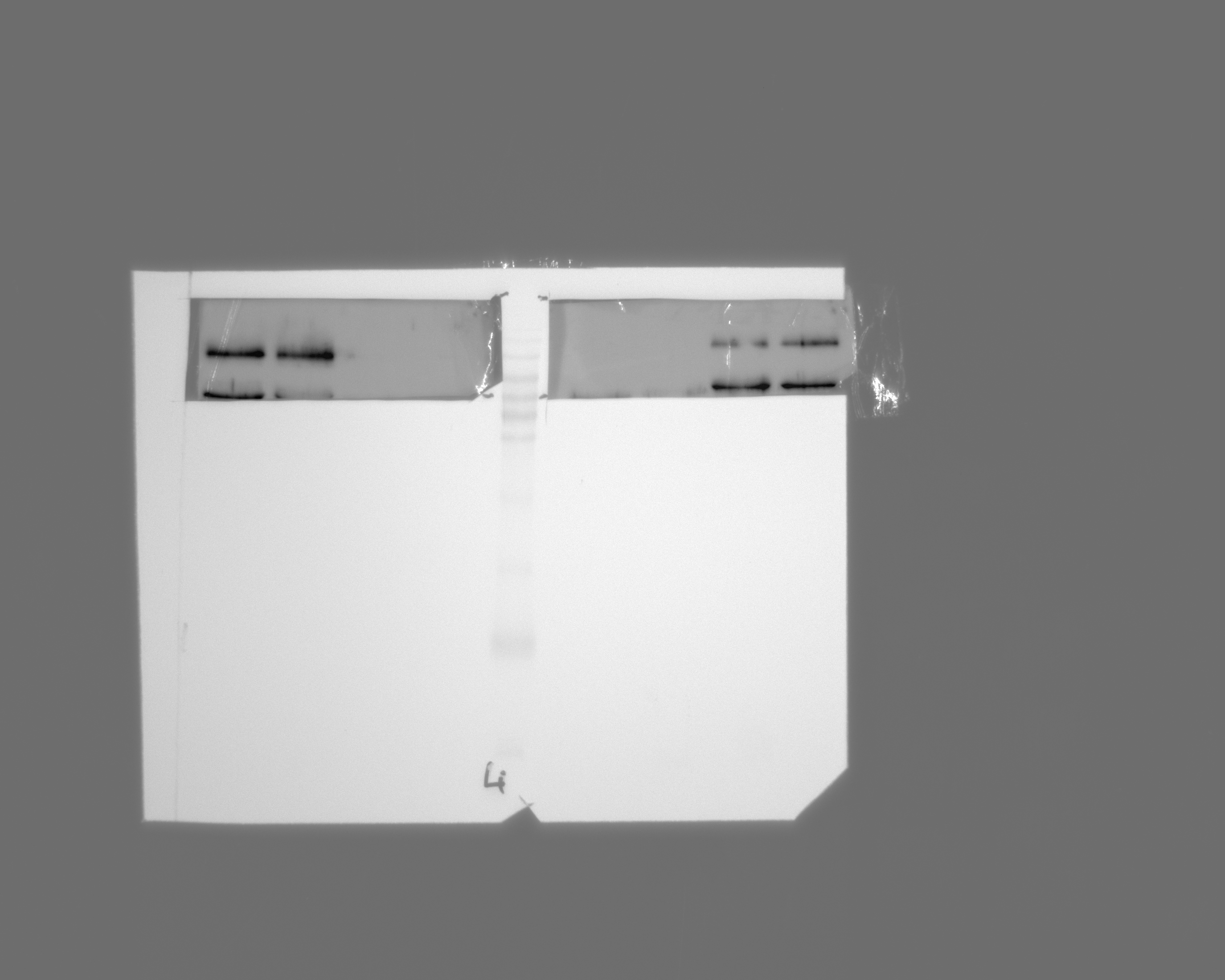


- +

- +

wt

mut

- +

- +

wt

mut

+ -

mut

wt

+ -

+ -

mut

wt

+ -

NCL

NPMexo

NPMendo

GFP

GFP (IP_RFP)&RFP (IP_GFP):
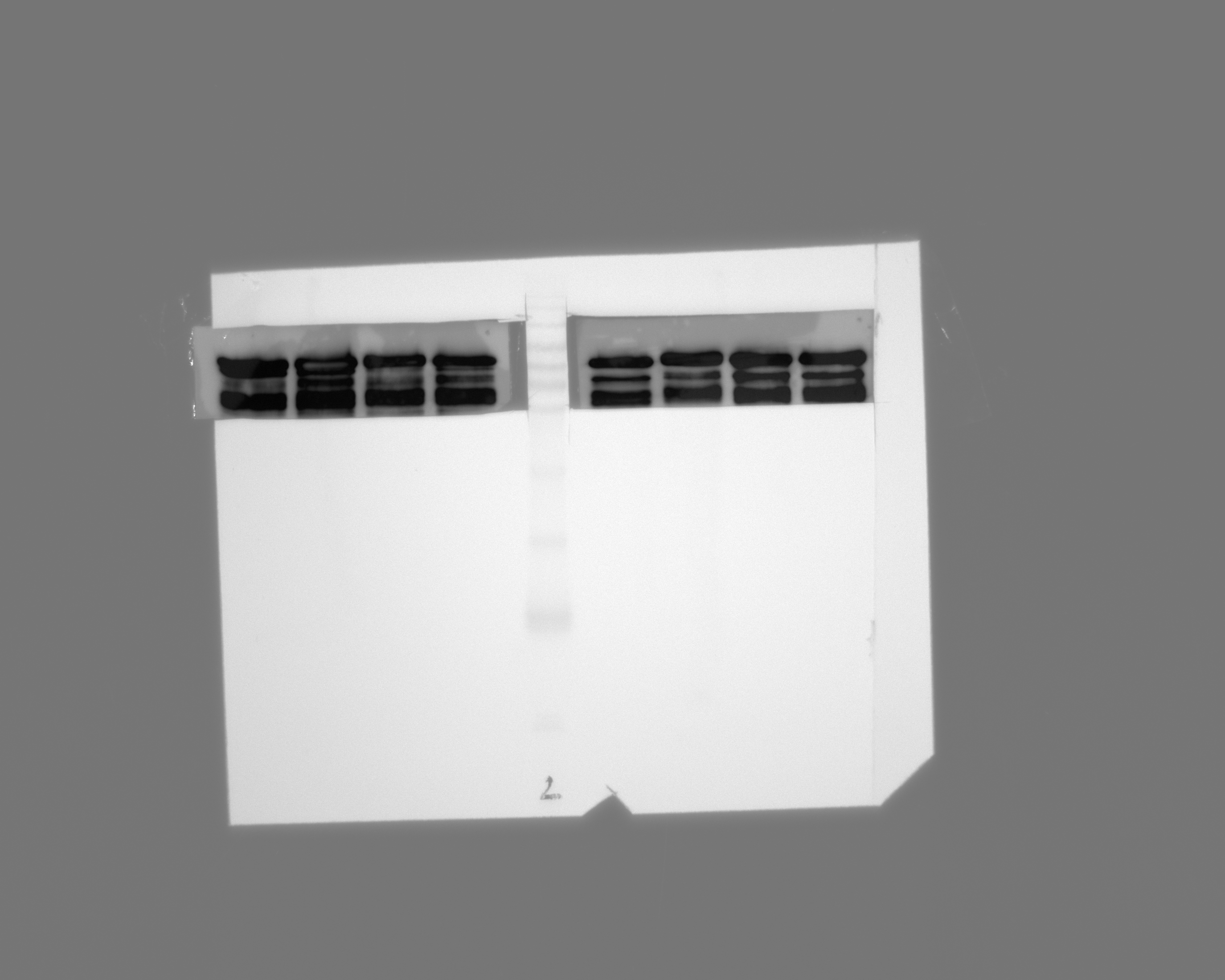


- +

wt

mut

- +

+ -

mut

wt

+ -

GFP

RFP

RFP&NPM (IP_RFP) & GFP&NPM (IP_GFP):
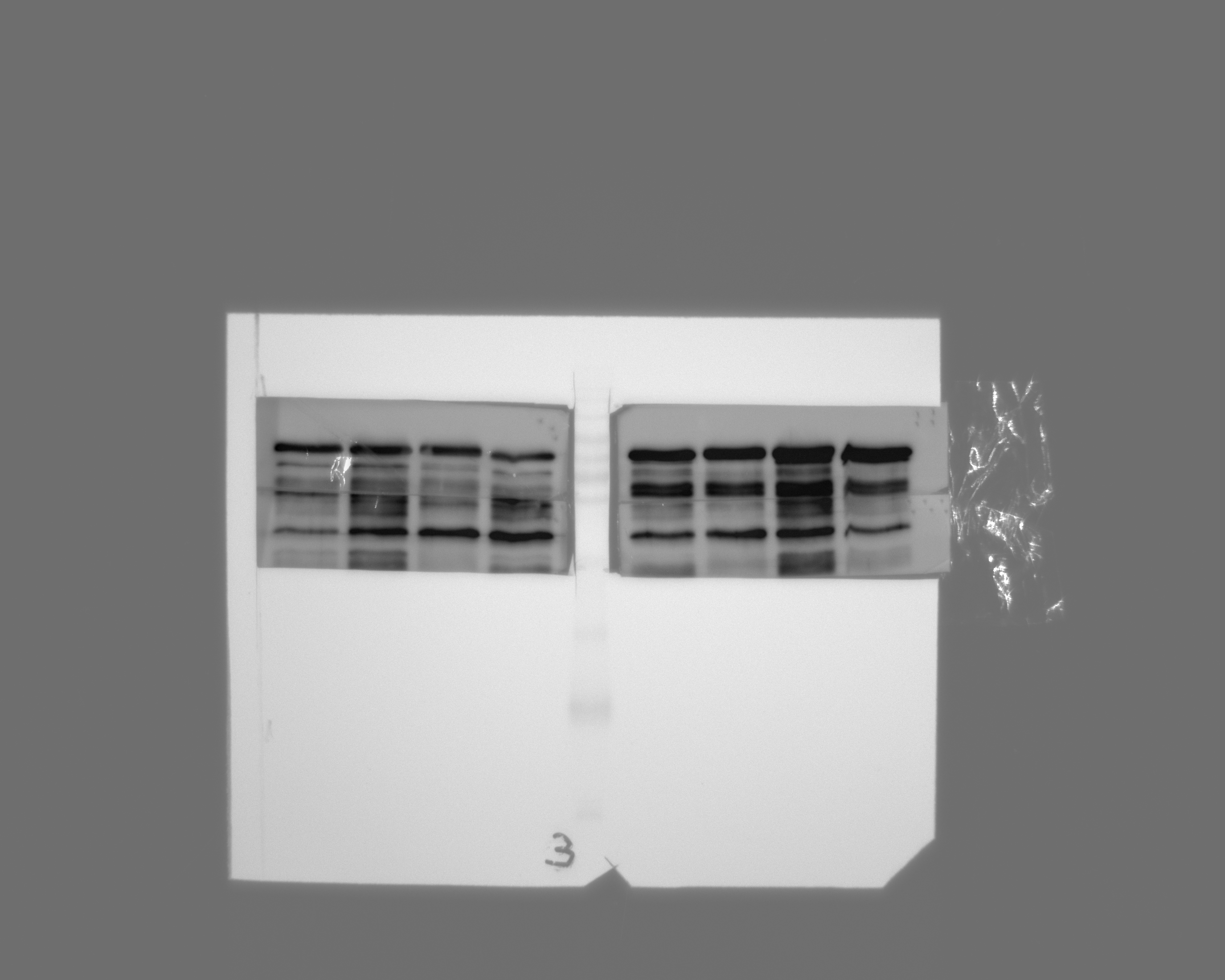


- +

- +

wt

mut

+ -

wt

mut

+ -

NPM endo

NPM endo

RFP

GFP

**Fig.14:**

pPAK1 Ser14
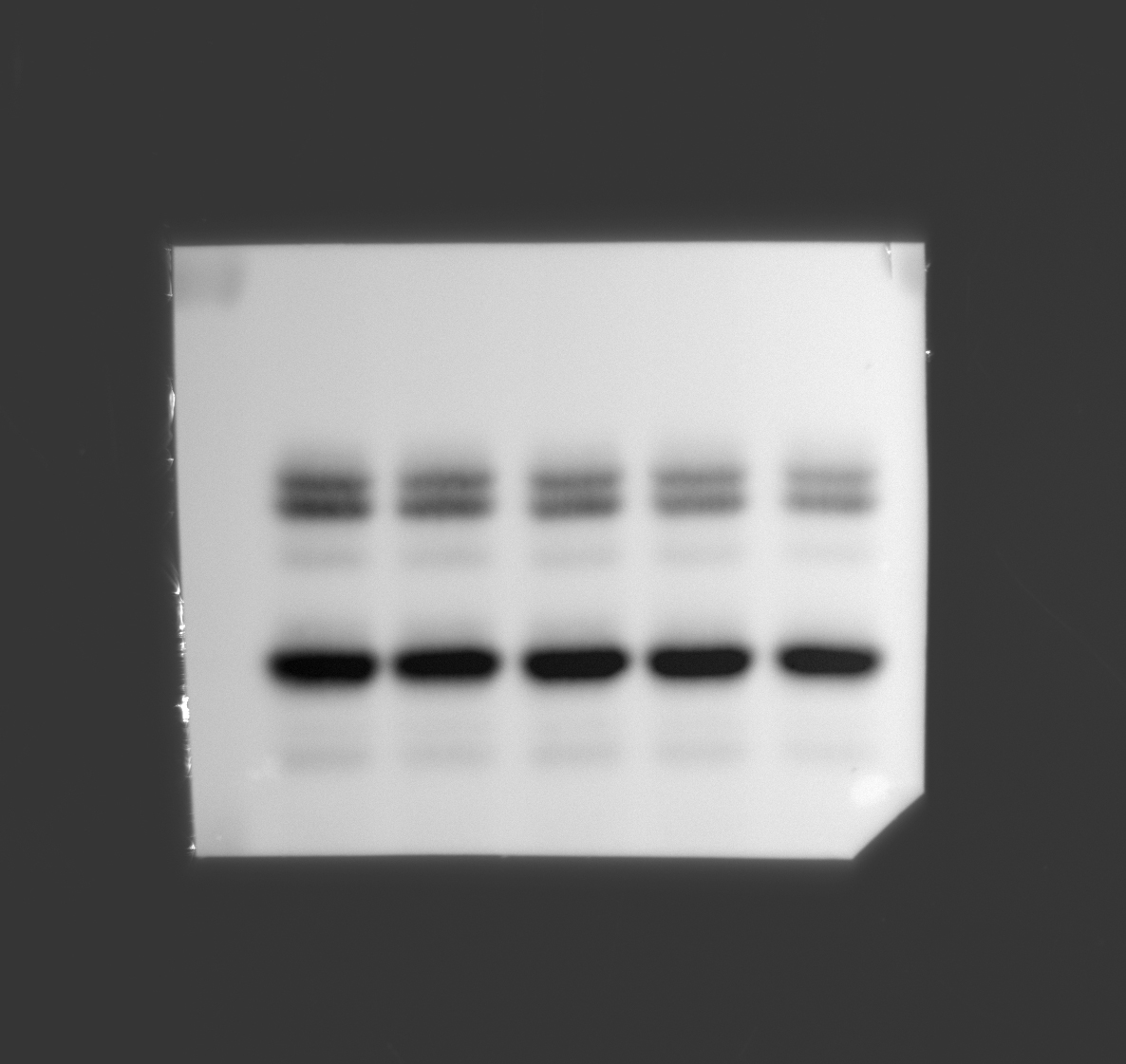
 PAK1
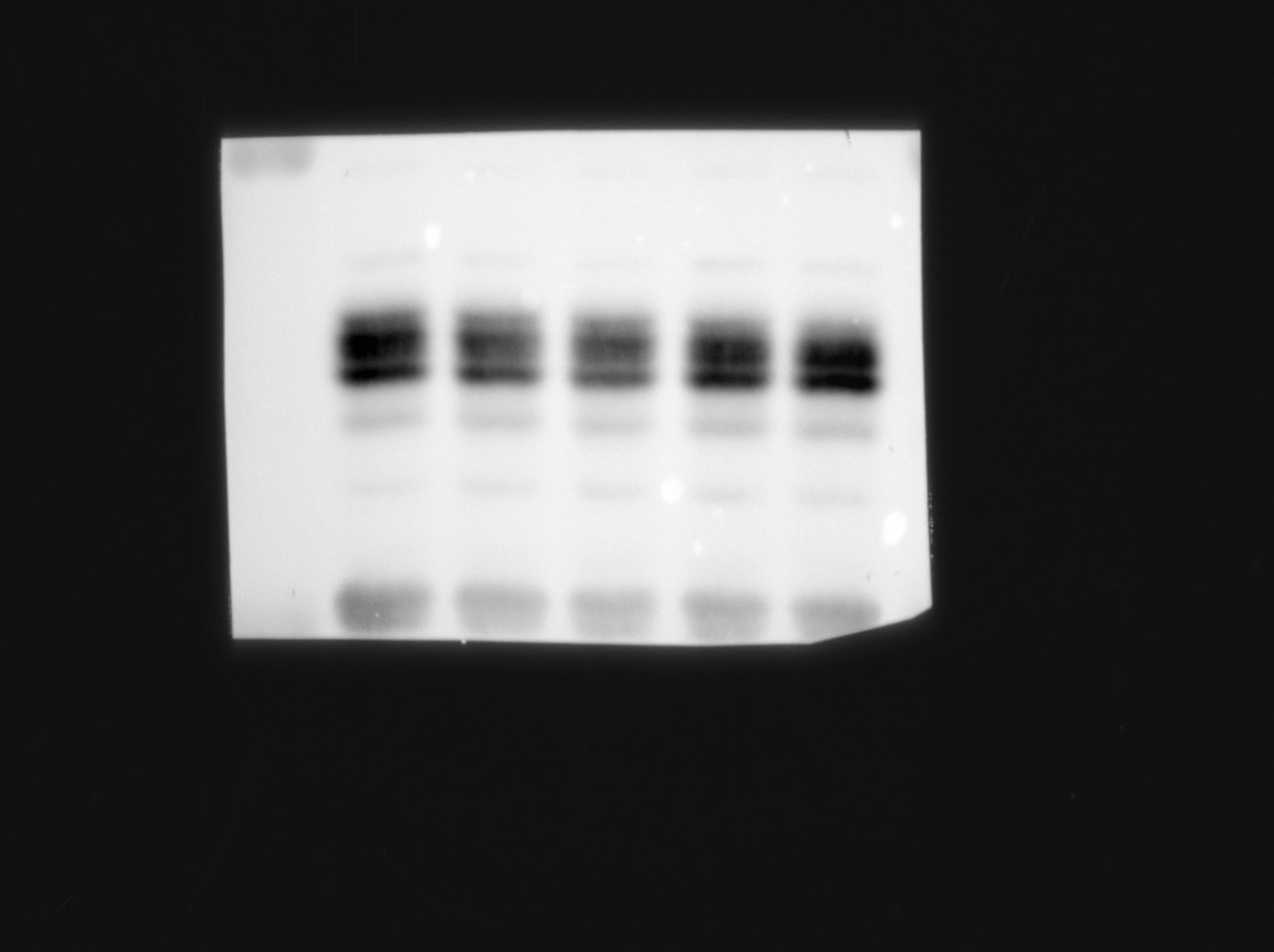


pCofilin Ser3
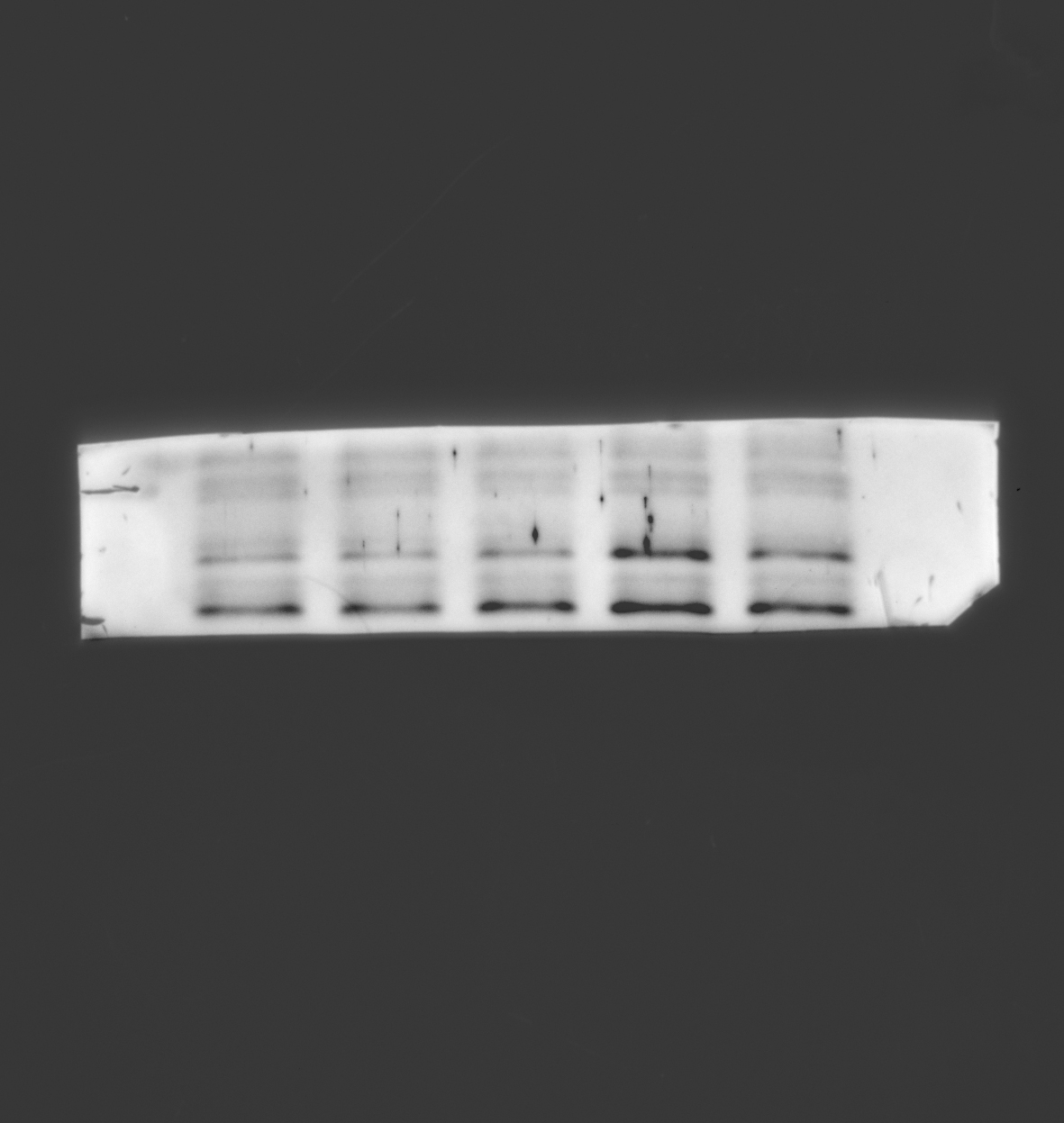
 Cofilin
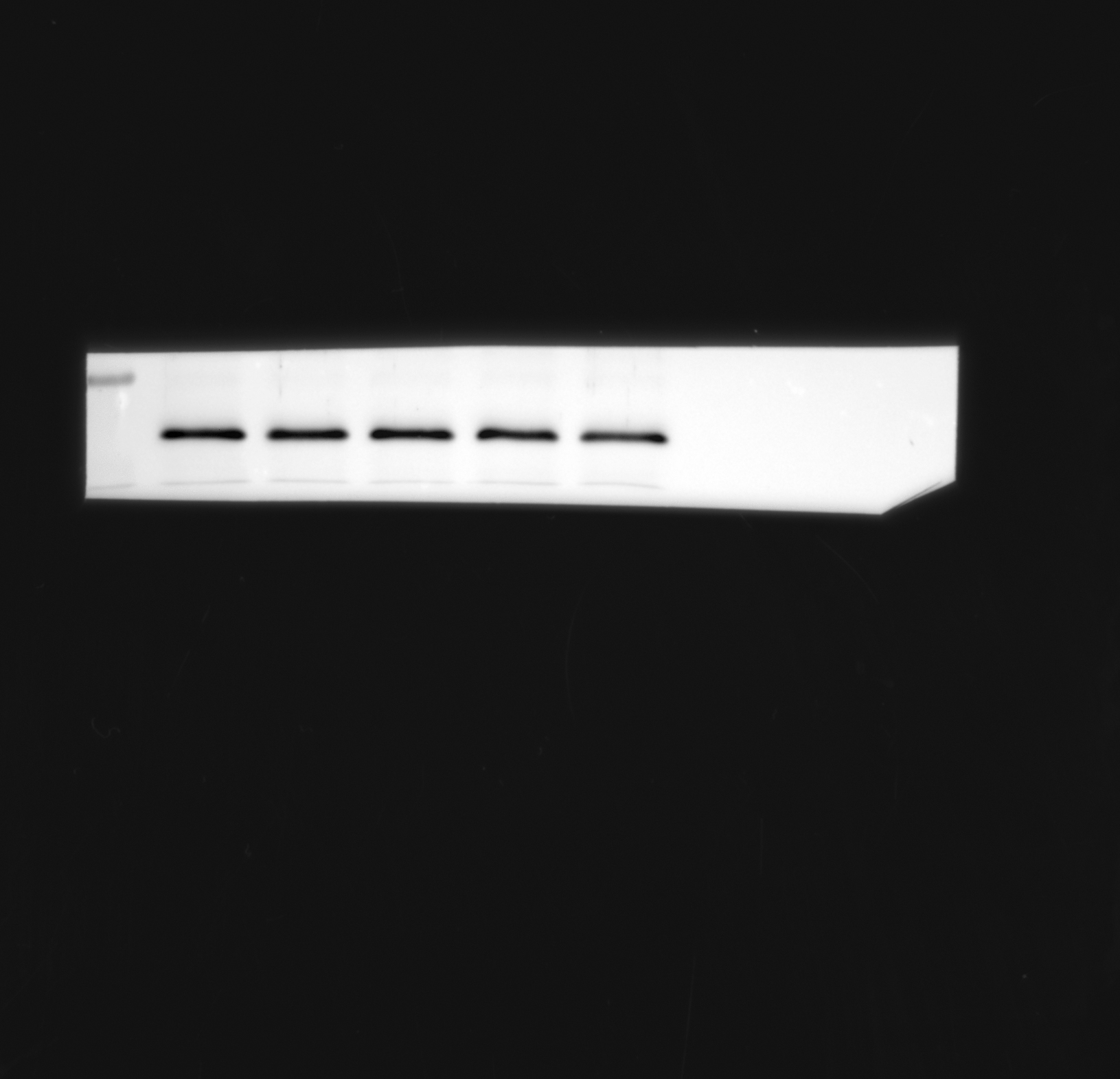


β-Actin
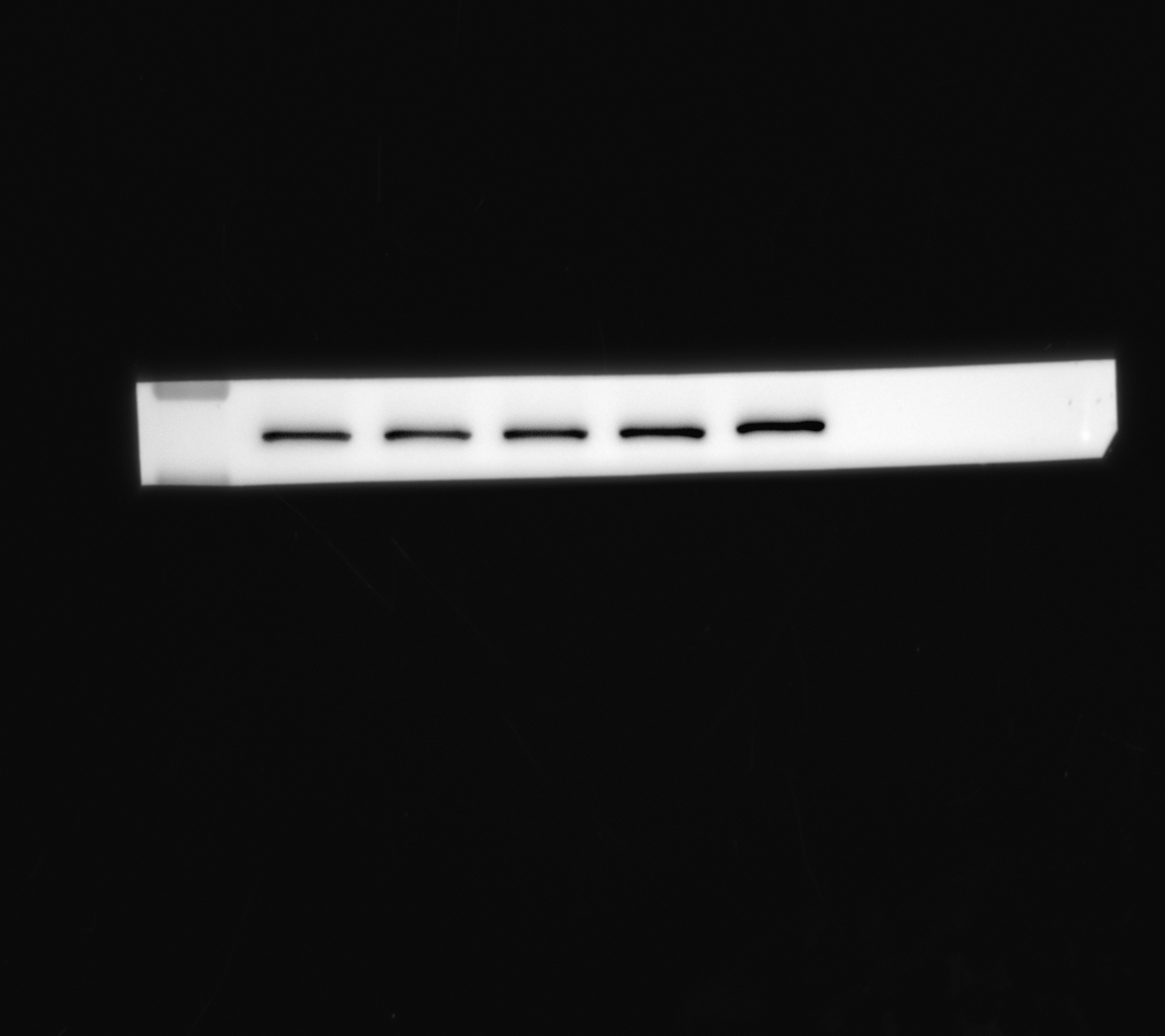


Fig. S2

NPM:
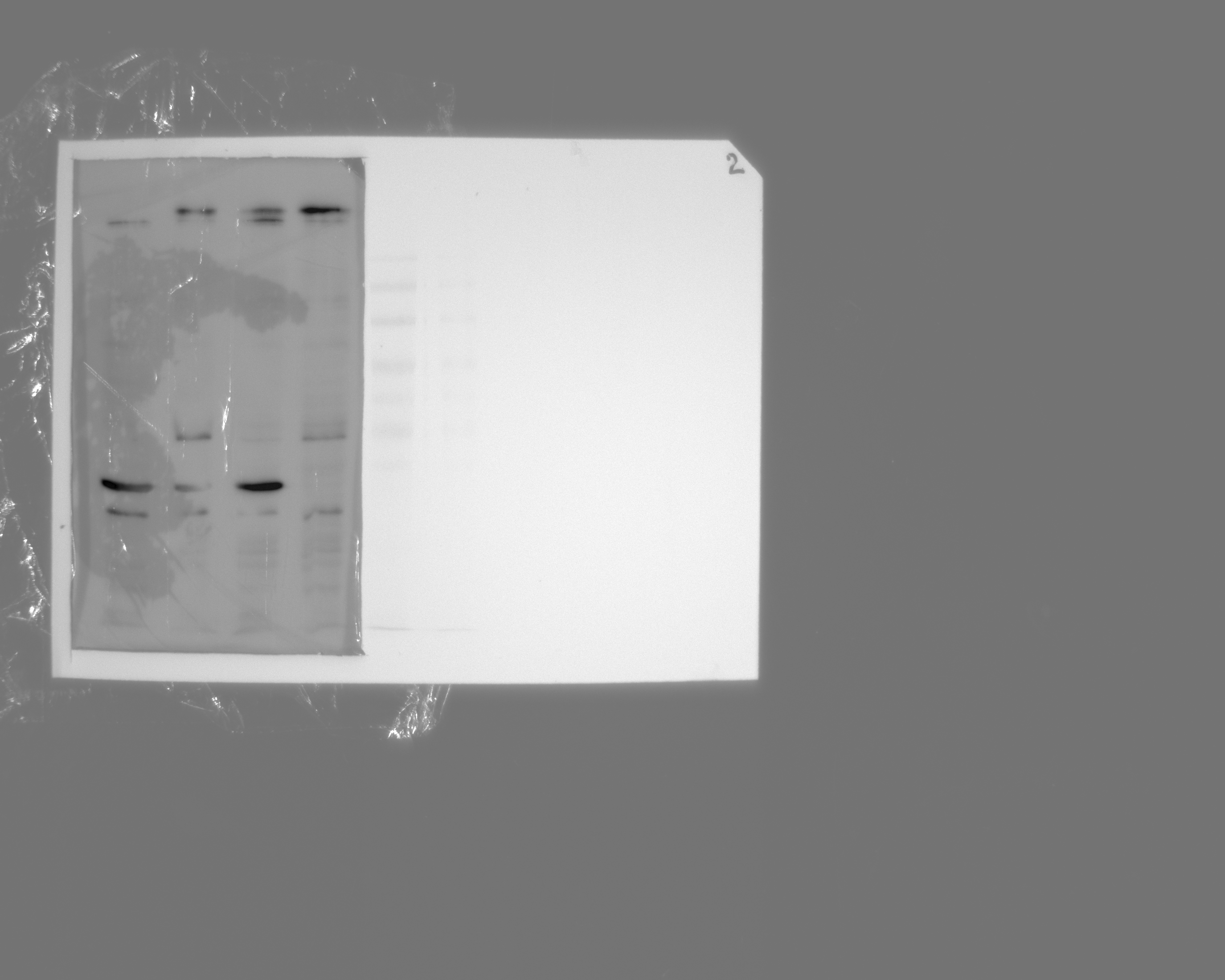


Δ117

wt

Δ117

Δ117

wt

Δ117

wt

wt

mono exo

mono endo

oligo exo

oligo endo

GFP and RFP:
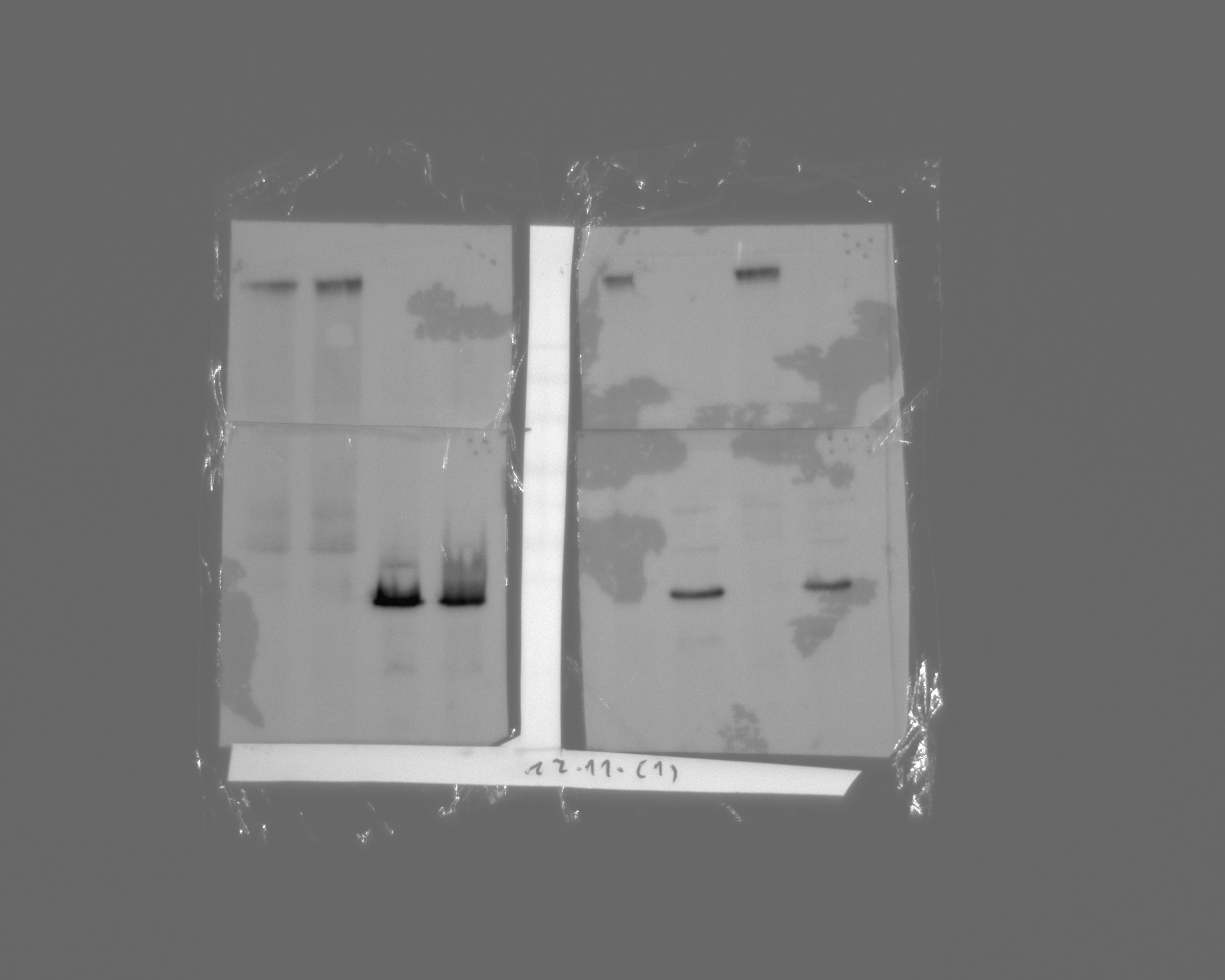


Δ117

wt

Δ117

Δ117

wt

Δ117

wt

wt

Δ117

Δ117

wt

wt

Δ117

wt

wt

Δ117

RFP

GFP

mono

oligo

Fig. S3:

RFP input:
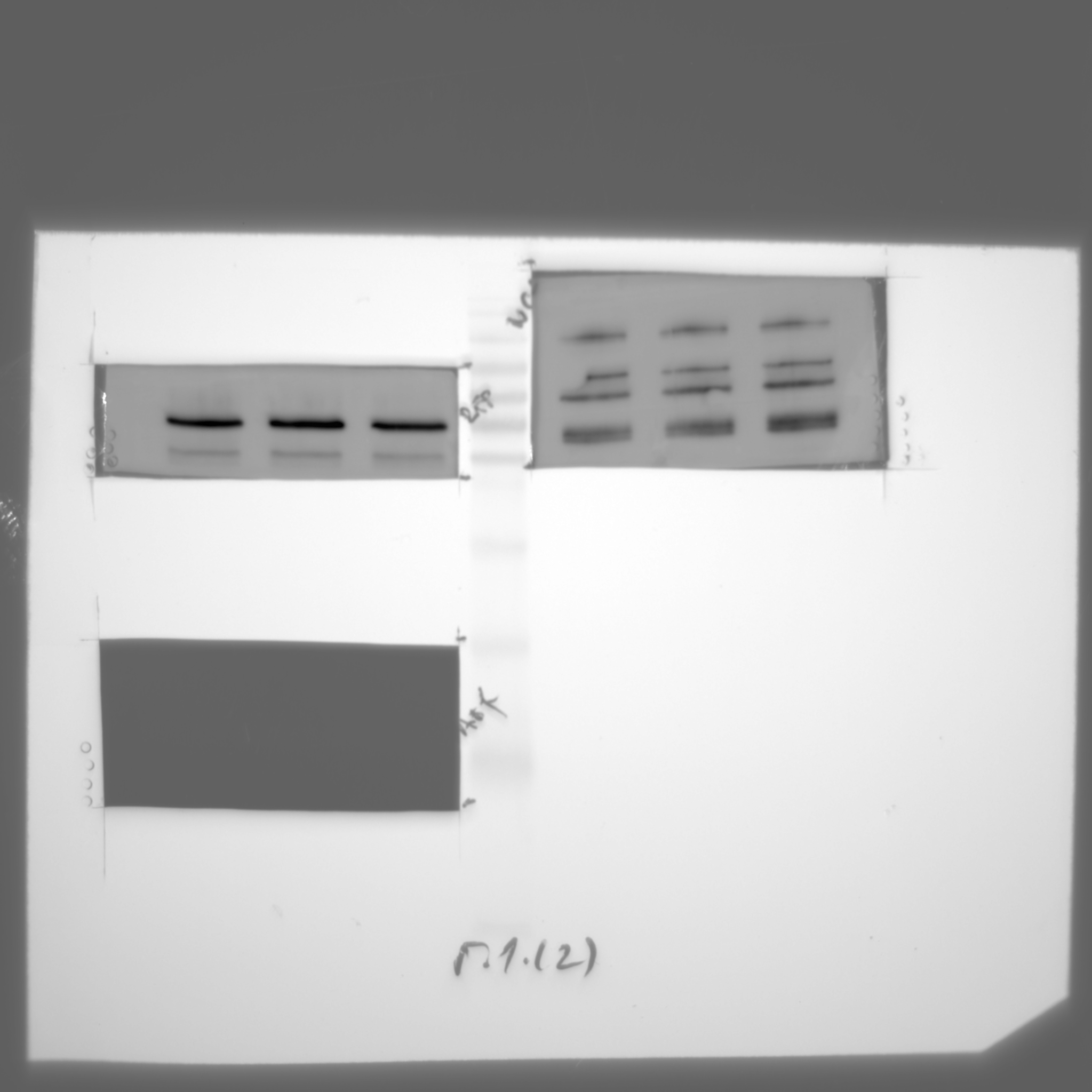
 GFP input:
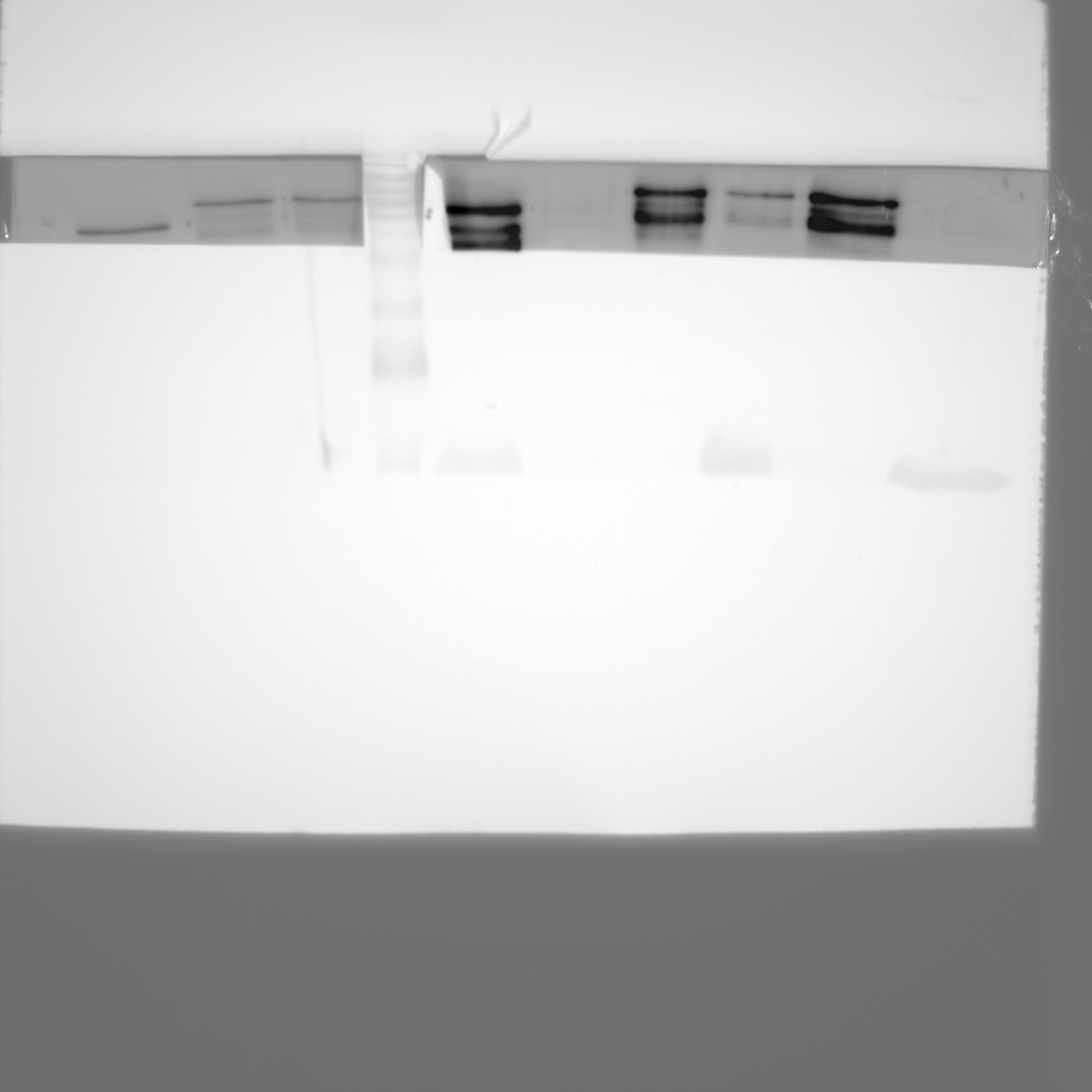


RFP

GFP

mut

Δ100

Δ100

Δ100

wt

Δ100

mut

Δ100

Δ100

Δ100

wt

Δ100

NPM input:
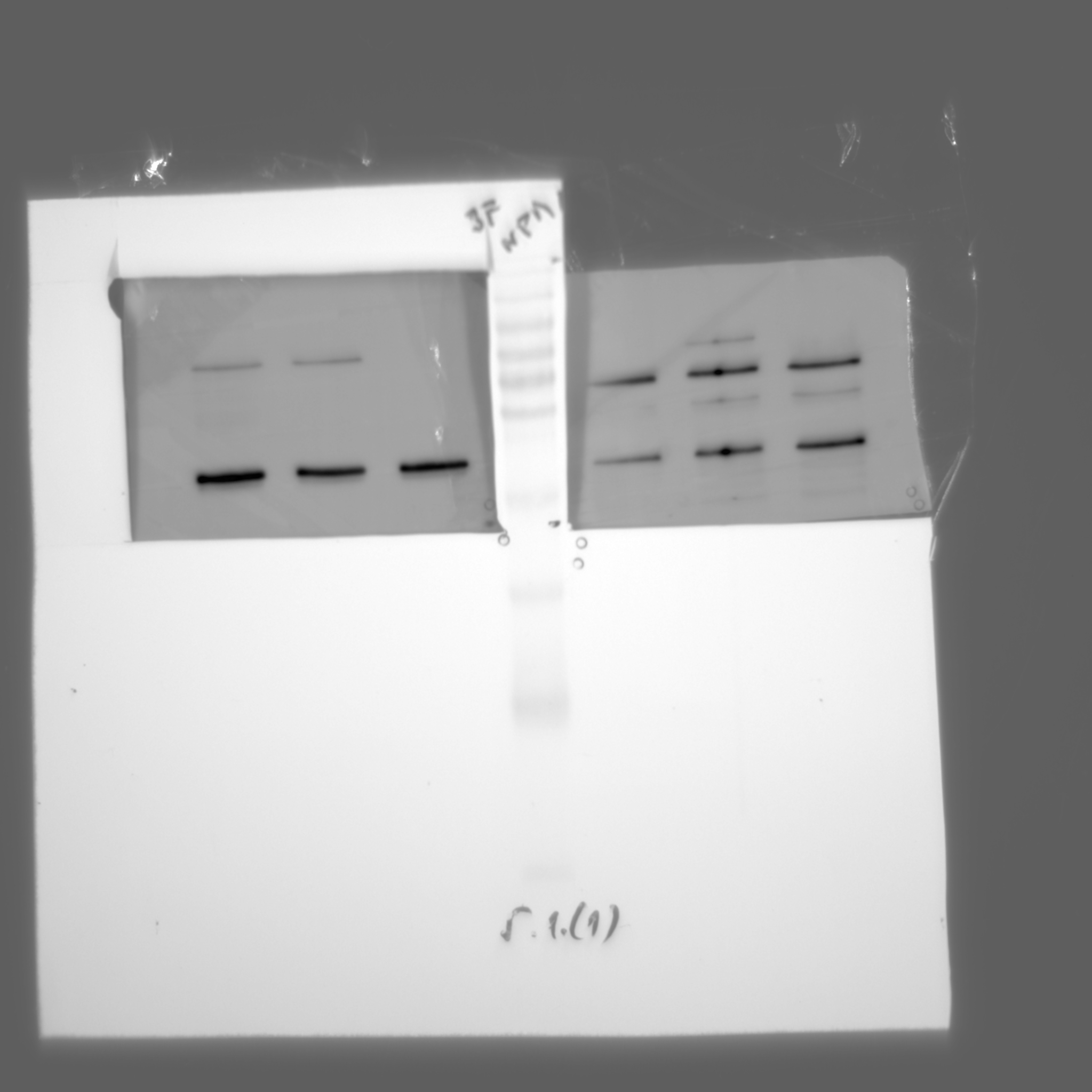
 Arf input:
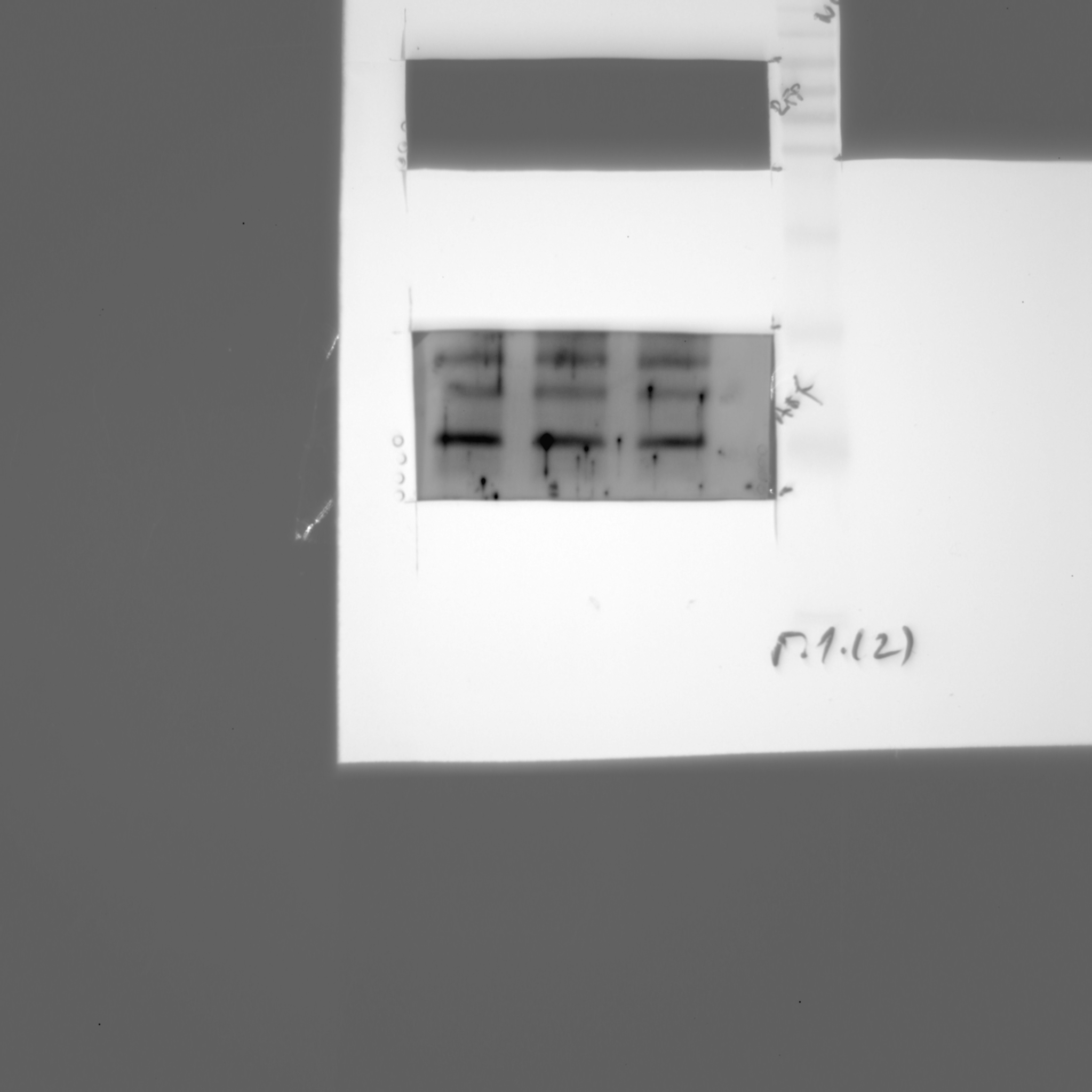


endo

Arf

NPM

Δ100

Δ100

wt

Δ100

mut

Δ100

mut

Δ100

Δ100

Δ100

wt

Δ100

GFP IPG & RFP IPR:
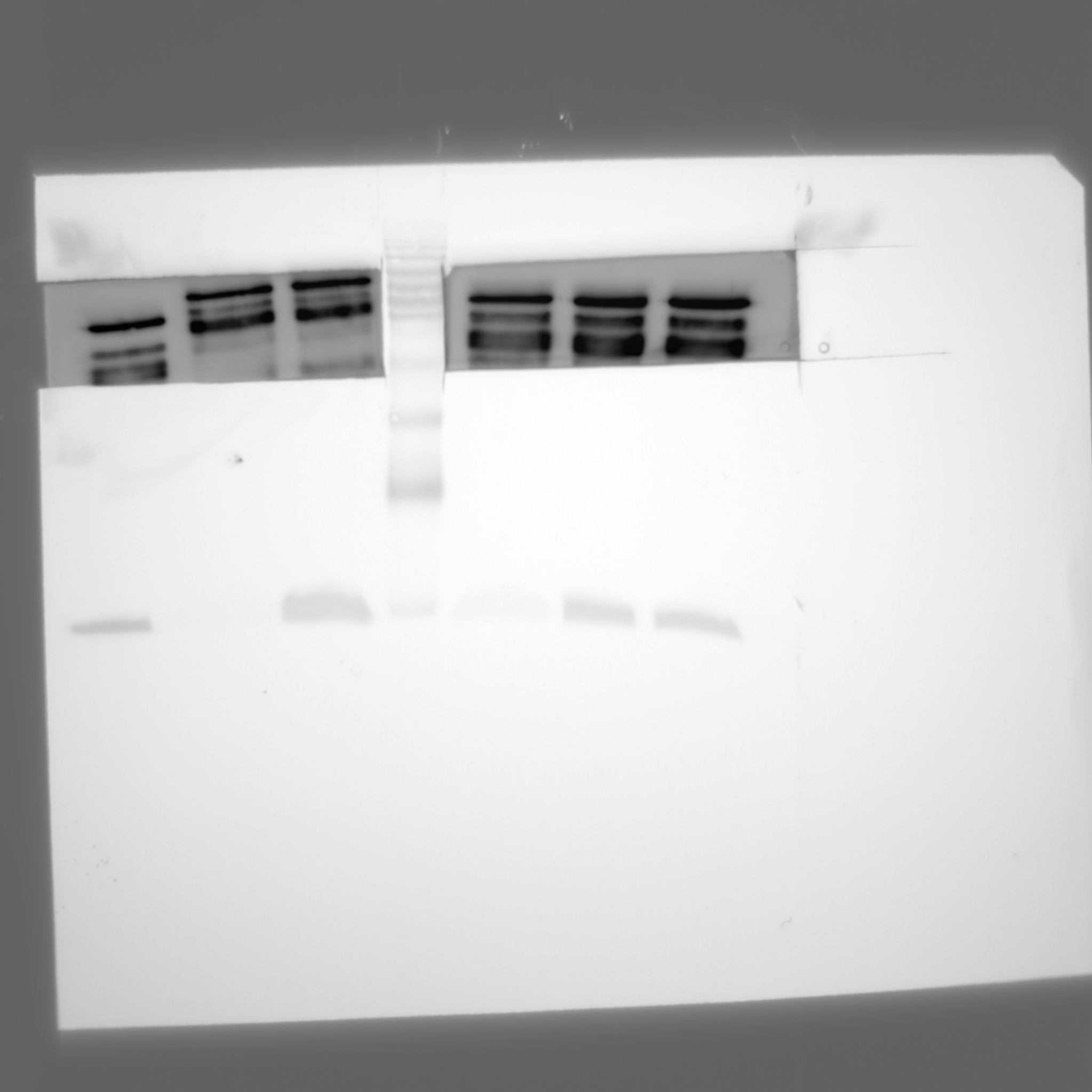


GFP

RFP

mut

Δ100

Δ100

Δ100

wt

Δ100

mut

Δ100

Δ100

Δ100

wt

Δ100

RFP IPG & GFP IPR:
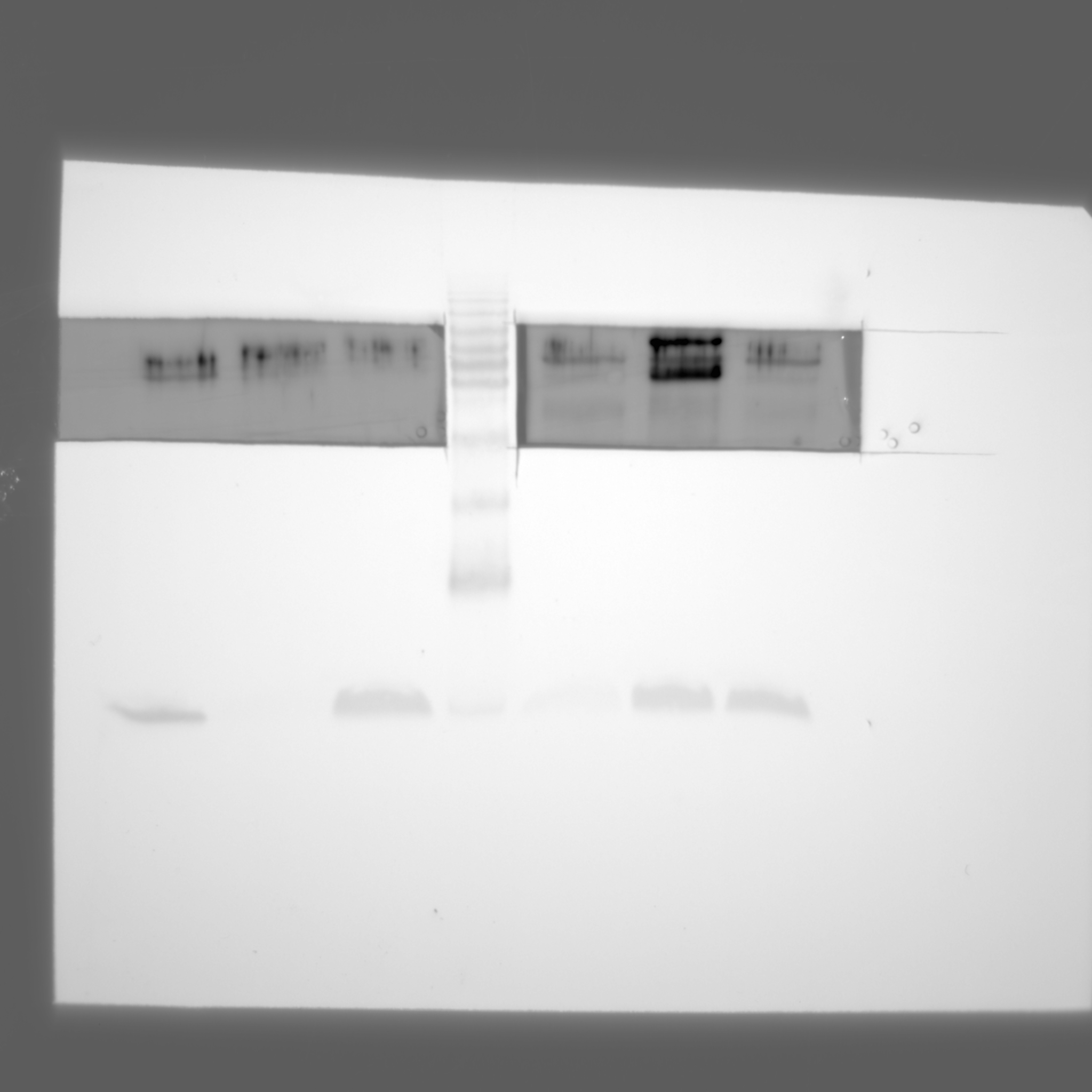


RFP

GFP

mut

Δ100

Δ100

Δ100

wt

Δ100

wt

Δ100

Δ100

Δ100

mut

Δ100

NPM IP:
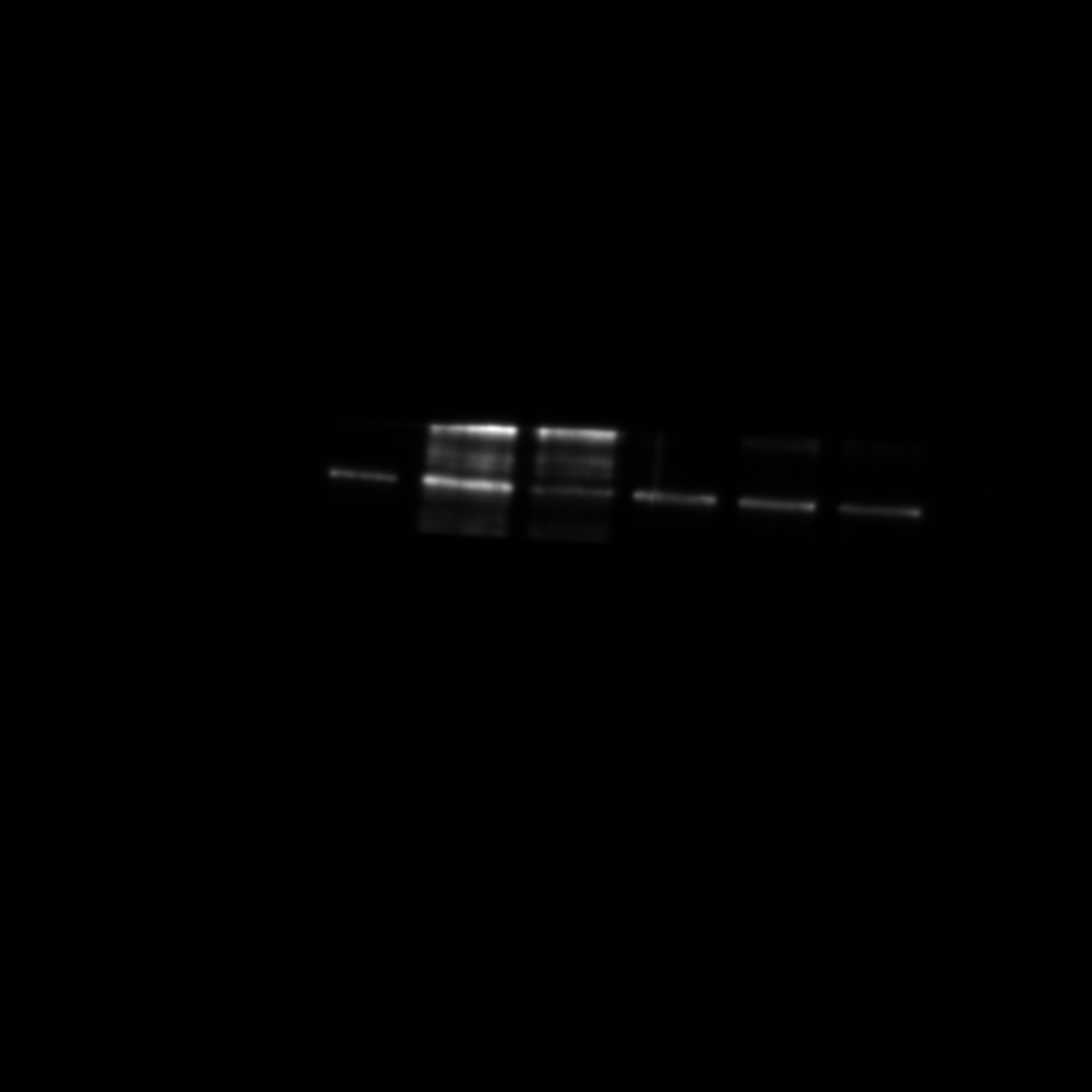
 Arf IP:
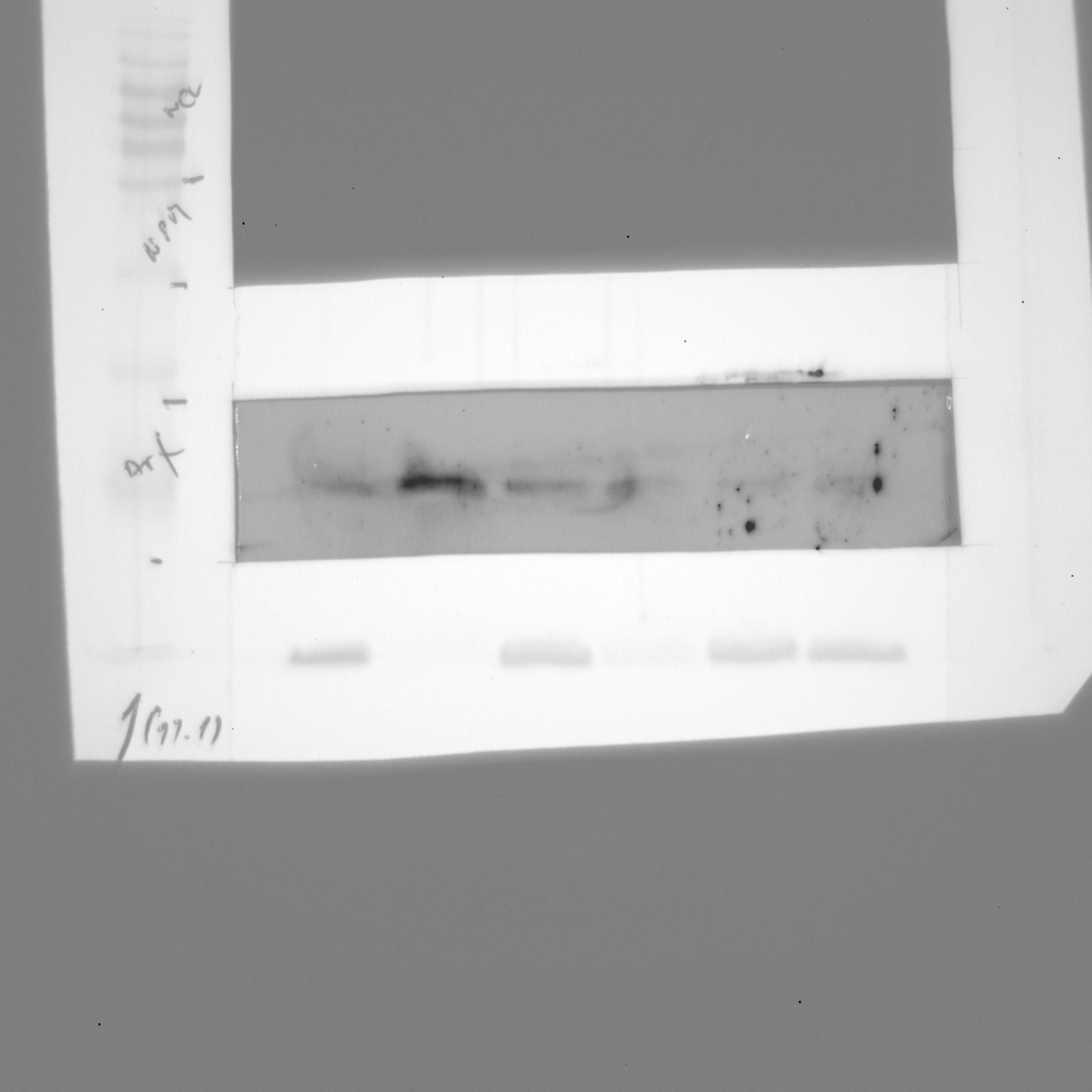


NPM

endo

endo

endo

Arf

mut

Δ100

Δ100

Δ100

wt

Δ100

mut

Δ100

Δ100

Δ100

wt

Δ100

wt

Δ100

Δ100

Δ100

mut

Δ100

mut

Δ100

Δ100

Δ100

wt

Δ100

Fig S4:

GFP native:
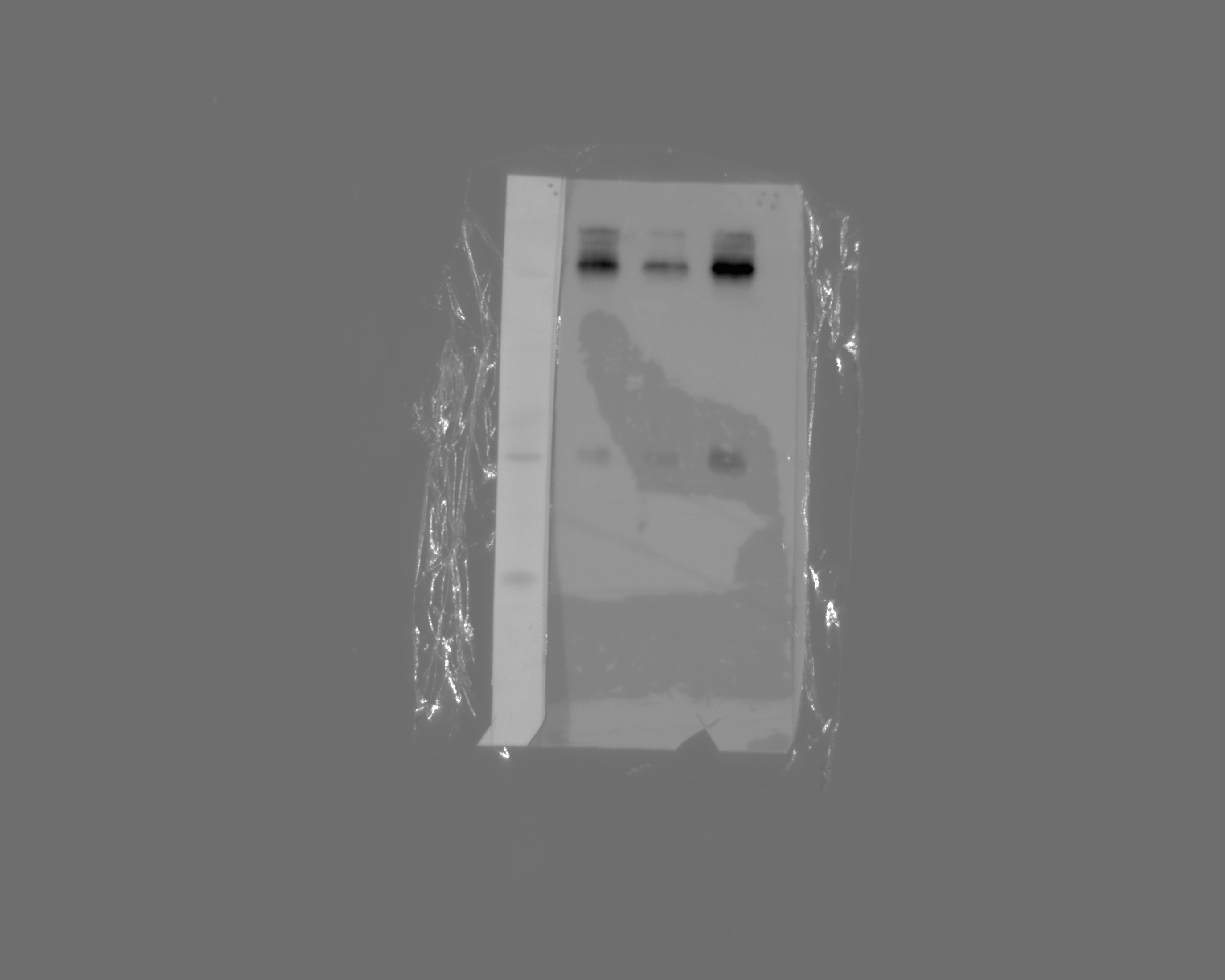


oligo

mono

mut

C21F

mut

wt

mut

wt

NSC

RFP native:
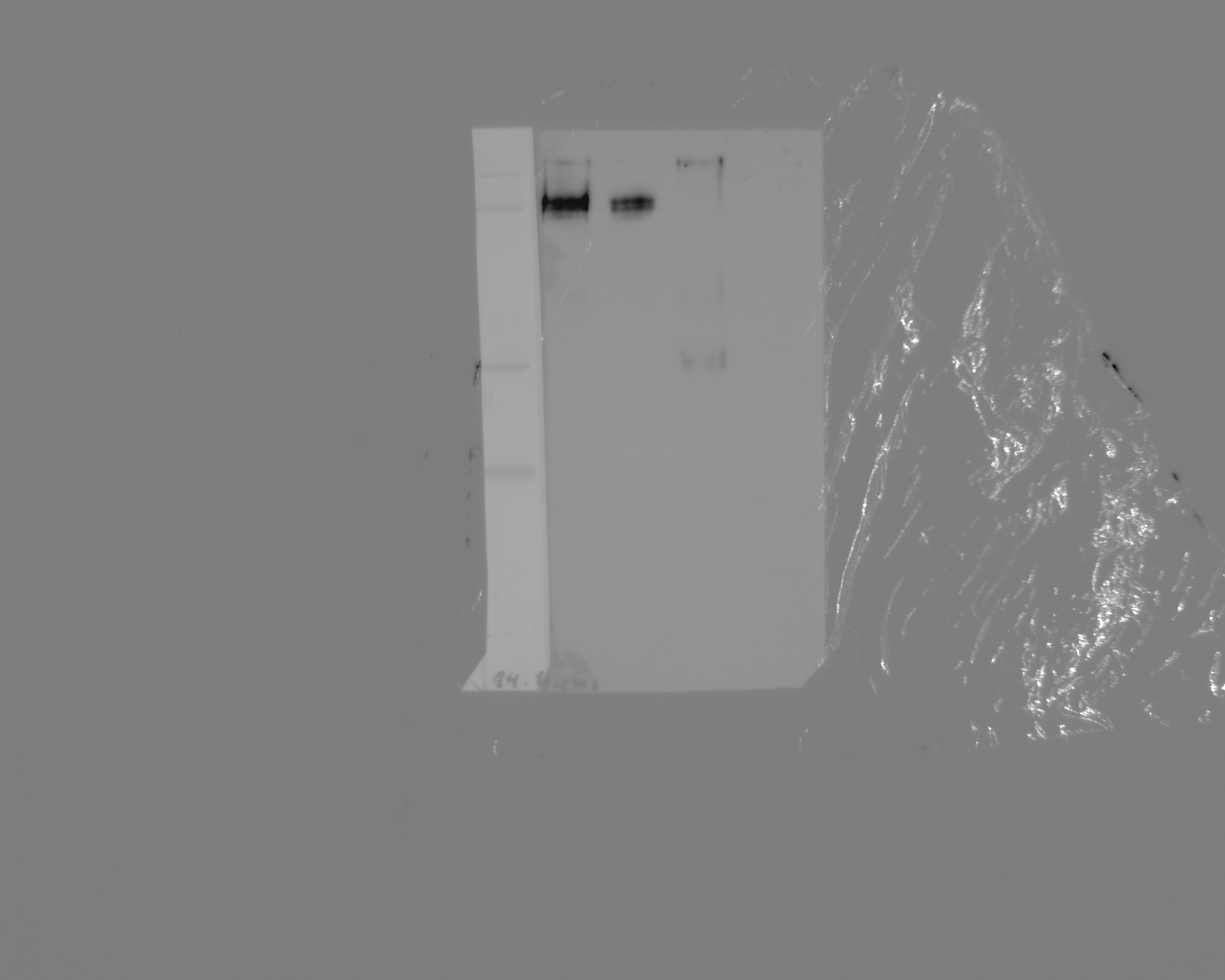


mono

oligo

mut

wt

mut

wt

NSC

mut

C21F

GFP seminative:
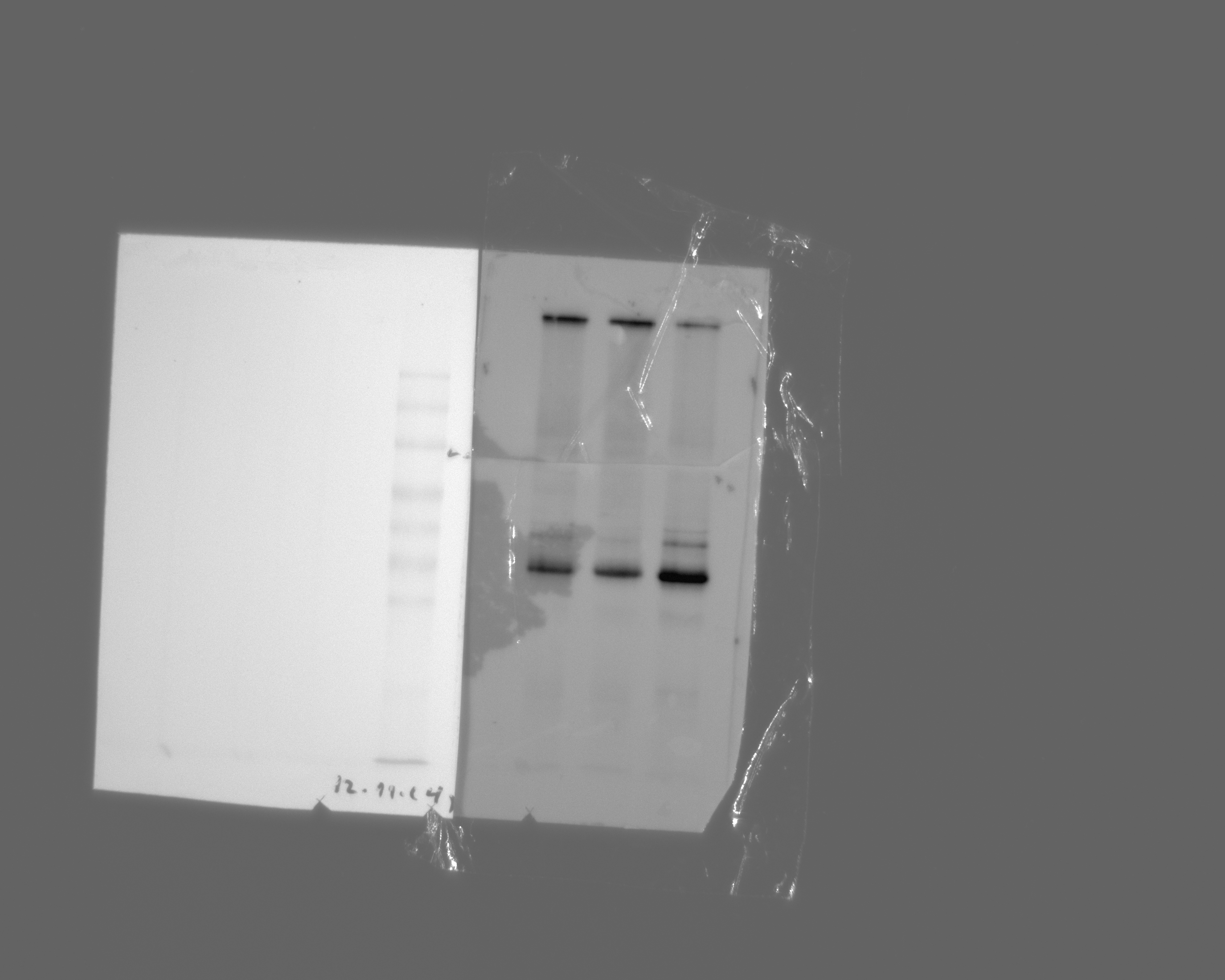


mono

oligo

mut

wt

NSC

mut

wt

mut

C21F

RFP seminative:
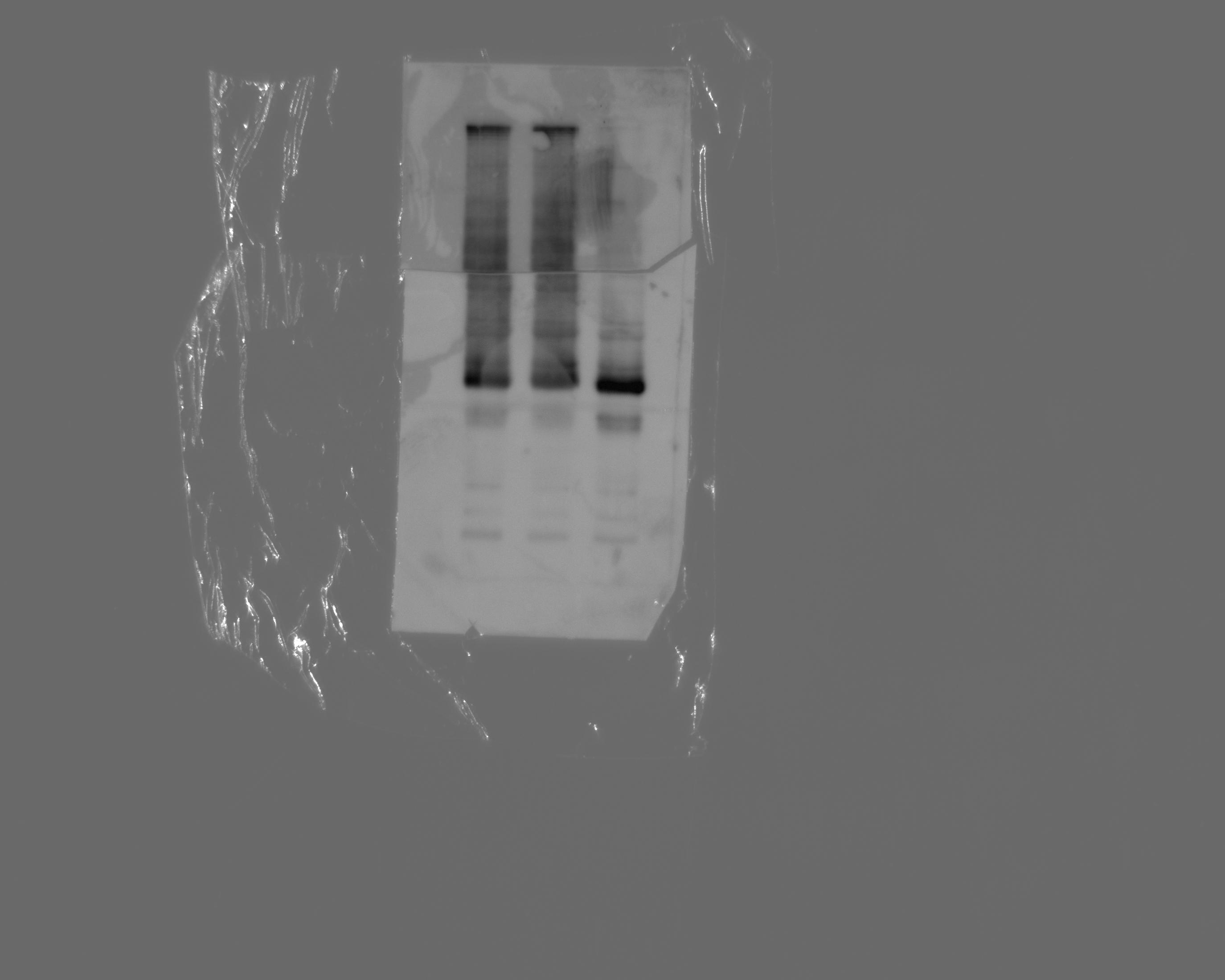


mono

oligo

mut

wt

NSC

mut

wt

mut

C21F
